# Safety and efficacy of trifluridine/tipiracil +/− bevacizumab plus XB2001 (anti-IL-1α antibody): a single-center phase 1 trial

**DOI:** 10.1038/s41392-024-02116-4

**Published:** 2025-01-17

**Authors:** Marion Thibaudin, Nicolas Roussot, Chloé Burlot, Antonin Schmitt, Julie Vincent, Zoé Tharin, Leila Bengrine, Hélène Bellio, Aurélie Bertaut, Léa Hampe, Susy Daumoine, Emilie Rederstorff, Morgane Peroz, Titouan Huppe, Valentin Derangère, David Rageot, John Simard, Caroline Truntzer, Jean David Fumet, Francois Ghiringhelli

**Affiliations:** 1 Centre de Recherche INSERM Center for Translational and Molecular Medicine, 21000 Dijon, France; 2 Cancer Biology Transfer Platform, Centre Georges-François Leclerc Equipe Labellisée Ligue Contre le Cancer, 21000 Dijon, France; 3 University of Bourgogne Franche-Comté, 21000 Dijon, France; 4 Genetic and Immunology Medical Institute, Dijon, France; 5 Department of Medical Oncology, Centre Georges-François Leclerc, 1 rue du Professeur Marion, 21000 Dijon, France; 6 Pharmacy Department, Centre Georges-François Leclerc, 1 rue Pr Marion, 21079 Dijon Cedex, France; 7 Methodolgy and biostatistics unit, GF Leclerc Center, Dijon, France; 8 Clinical research center, Centre Georges-François Leclerc, Dijon, France; 9 XBiotech, Austin, TX USA

**Keywords:** Clinical trials, Translational research, Immunotherapy

## Abstract

In the tumour microenvironment, IL-1α promotes neoangiogenesis, matrix remodelling, tumour proliferation, chemoresistance, and metastases. Highly expressed in human colorectal cancers, IL-1α is associated with poor prognosis. XB2001, a fully human monoclonal antibody neutralizing IL-1α, was evaluated for safety and preliminary efficacy with trifluridine/tipiracil (FTD/TPI) and bevacizumab in metastatic colorectal cancer patients previously treated with oxaliplatin- and irinotecan-based chemotherapies. This single institution, phase 1 study used a 3 + 3 design to assess XB2001 at doses of 250 mg, 500 mg and 1000 mg every 14 days, associated with FTD/TPI 35 mg/m² (days 1–5 and 8-12, every 28 days) (NCT05201352). The Maximum Tolerated Dose of XB2001 + FTD/TPI was then associated in combination with bevacizumab (5 mg/kg, days 1 and 15). Safety, efficacy, pharmacokinetics and pharmacodynamics were assessed. Seventeen patients (median age: 67.4 years) were enroled. No patient exhibited dose-limiting toxicity at any dose. The most common treatment-related adverse events (TRAE) of any grade (G) were diarrhoea (35.3%), nausea (47.1%) and anaemia (35.3%). G3-4 TRAE were neutropenia (17.6%) hypertension and infection (5.9% each). The RP2D (recommended phase 2 dose) of XB2001 was 1000 mg. The disease control rate was 76%, with 23% of patients achieving an objective response, including one complete response. Response and longer progression-free survival were associated with a decrease in serum IL-6 levels during therapy. High intratumoral IL-1α expression at baseline and CD8/PD-L1 infiltration are associated with a better progression-free survival. The combination of XB2001 with FTD/TPI and bevacizumab is feasible and safe, and showed encouraging clinical activity in chemotherapy-resistant mCRC.

## Introduction

Colorectal cancer (CRC) is the second most frequently diagnosed cancer in Europe, and a leading cause of mortality both in Europe and globally.^1^ Chemotherapy remains the main treatment for metastatic CRC (mCRC). Current regimens typically involves three cytotoxic agents, namely fluoropyrimidine, oxaliplatin and irinotecan – combined with targeted therapies, such as monoclonal antibodies against EGFR (e.g., panitumumab or cetuximab) or VEGF (e.g., bevacizumab or aflibercept).^2^ In patients with deficient mismatch repair status, first-line treatment relies on the use of pembrolizumab.^3^ However, when these treatments fail, few therapeutic options remain. Regorafenib, fruquintinib and trifluridine/tipiracil (FTD/TPI) became recently new players in the therapeutic arsenal as third-line therapies.^4–6^ Recently, association of trifluridine/tipiracil + bevacizumab became standard best third-line.^7^

Chronic inflammation is widely accepted as a key signal in promoting carcinogenesis and tumour progression.^8^ For CRC, chronic intestinal inflammation, such as inflammatory bowel disease, is associated with CRC carcinogenesis.^9^ In malignant disease, inflammatory signals play crucial roles in cancer growth by promoting angiogenesis, tumour stroma remodelling, tumour invasion, metastatic diffusion and cachexia. IL-1α and β are key pro-inflammatory proteins. Polymorphisms in *IL1A, IL1B* and *IL1RA* genes activate the IL-1 pathway and are associated with an increased risk of tumour relapse in localized CRC.^10^ IL-1β is mainly produced by myeloid lineages in the tumour tissue and could contribute significantly to sustaining chronic inflammation. In contrast, IL-1α is typically expressed by malignant cells.^11^ IL-1α pro-oncogenic property is related to its capacity to trigger NFKB and STAT3 pathway activation and to promote tumour aggressiveness.^12^ IL-1 is also a key regulator of IL-17 production, which is associated with poor prognosis in CRC^13^ and IL-17A blockade reduces tumour progression in preclinical models of CRC.^14^ Genetic deletion of *Il1r1* in colon epithelial cells alleviated tumorigenesis in a mouse model of CRC.^15^ Our group has also demonstrated the ability of Anakinra, a molecule that blunts IL-1α and IL-1β, to enhance the efficacy of 5-Fluorouracil in mice, as well as the combination of 5-Fluorouracil and bevacizumab in patients.^16,17^ Finally, CRC is characterized by significantly higher IL-1α expression compared to normal colon tissue.^15^

Based on this rationale, it has been proposed that neutralization of IL-1α could have the potential to reduce tumour growth and reverse or improve antitumoral immune response in CRC. The monoclonal antibody MABp1 which neutralizes IL-1α was tested in human mCRC and was shown to improve patient symptoms and quality of life compared with placebo, reinforcing the idea that targeting the IL-1 pathway is relevant for the treatment of mCRC.^18^ Bermekimab (MABp1) and XB2001 were generated from two different healthy human donors. MABp1 is an IgG1, while XB2001 is an IgG4. Although the variable fragment sequence differs between the two mAbs, the IC50 and KD of XB2001 are comparable to those of MABp1, albeit slightly higher for XB2001.

In this study, we tested the safety and efficacy of a combination of the new IL-1A-targeting monoclonal antibody XB2001, with FTD/TPI and bevacizumab.

## Results

### Patient characteristics

A total of 17 patients were included in this single-centre study between October 2022 (first patient included) and November 2023 (last patient included). By 24 January 2024, all patients had completed the DLT period and by 5 March 2024 the second efficacy evaluation imaging. The median follow-up for clinical activity was 14.1 months (range: 1.4–18 months) and the median treatment duration was 7 months (range: 1–17). The safety follow-up data presented cover the 2 first cycle of treatment (2 months). The baseline patients’ characteristics are presented in Table 1. Median age was 67.4 years (range: 49–81), with 9/17 (53%) were male, and 9/17 (53%) had an ECOG performance-status of 1. Thirteen (76.5%) patients had RAS mutated status and more than half of patients had left side tumours (13/17 (76.5%)). The median number of previous lines of treatments was 3 (range: 1–5), median number of metastatic sites was 2 (range: 1–4), and 10 (59%) patients presented liver metastases.

**Table 1:** Patient characteristics

*Characteristics*	*(N* = *17 patients)*
Sex	
Male - no. (%)	9 (53)
Female - no. (%)	8 (47)
Age	
*N*	17
Mean (Standard deviation)	67,4 (8.5)
Median [min - max]	69 [49–81]
Primary site	
Colon - no. (%)	13 (76.5)
Rectal - no. (%)	4 (23.5)
Sidedness	
Left side (colon and rectum) - no. (%)	13 (76.5)
Right side - no. (%)	4 (23.5)
Median duration of disease before inclusion — mo (range)	38 (8-50)
No. of metastatic sites	
1 or 2 - no. (%)	11 (64.7)
≥ 3 - no. (%)	6 (35.3)
RAS Mutational status	
Mutated - no. (%)	13 (76.5)
Wild-type - no. (%)	4 (23.5)
MMR status	
dMMR - no. (%)	0 (0)
pMMR - no. (%)	17 (100)
No. of previous treatments for metastatic disease	
1 - no. (%)	1 (5.9)
2 - no. (%)	7 (41.2)
≥3 - no. (%)	9 (52.9)
ECOG performance-status score	
0 - no. (%)	8 (47)
1 - no. (%)	9 (53)
Previous treatment received for metastatic disease	
Fluoropyrimidine - no. (%)	17 (100)
Irinotecan - no. (%)	17 (100)
Oxaliplatin - no. (%)	17 (100)
Anti-VEGF moonoclonal antibody - no. (%)	17 (100)
Anti-EGFR monoclonal antibody - no. (%)	4 (23.5)
Regorafenib - no. (%)	3 (17.6)

### Safety

There were no XB2001 related infusion reactions. In the safety analysis, the maximum tolerated dose was not reached. The highest dose administered was considered as the RP2D (1000 mg every 2 weeks with bevacizumab).

We observed 3 serious adverse events (SAEs), reported in only 3 patients (17.6%). Based on the investigator’s report, no SAEs were related to XB2001, two were reported as disease-related and one was trifluridine/tipiracil-related. Two patients discontinued the study before the end of cycle 2 due to a SAE related to grade 3 (G3) intestinal obstruction related to disease progression. Most treatment-related AEs were grade 1 or 2 (Table 2). G3 and G4 AEs accounted for 7% of total AEs, and the most common AE at all dose levels were nausea/vomiting, anaemia, thrombopenia. The incidence of SAEs and AEs did not increase with increasing dose of XB2001 (Supplementary Table 1 summarized SAEs and AE reported during the first months of treatment, while Supplementary Table 2 provided data on all SAEs and AE observed during the follow-up period, categorized by toxicity grade). Supplementary Table 3 indicated treatment discontinuations and the reasons for postponed treatment.

**Table 2:** Adverse events related to drug administration upon investigator’s report

*System organ disorder*	*All Grades*	*Grade 1/2*	*Grade 3/4*
	*No. (%)*	*No. (%)*	*No. (%)*
Gastrointestinal disorders			
Diarrhoea	6 (35.3%)	6 (35.3%)	0
Nausea	8 (47.1%)	8 (47.1%)	0
Vomiting	4 (23.5%)	4 (23.5%)	0
Abdominal pain	3 (17.6%)	3 (17.6%)	0
Constipation	3 (17.6%)	3 (17.6%)	0
General disorders			
Asthenia	4 (23.5%)	4 (23.5%)	0
Decreasing appetite	4 (23.5%)	4 (23.5%)	0
Vascular disorders			
Hypertension	1 (5.9%)	0	1 (5.9%)
Hypotension	1 (5.9%)	1 (5.9%)	0
Immune system disorders			
Infection	2 (11.8%)	1 (5.9%)	1 (5.9%)
Skin and subcutaneous tissue disorders			
Stomatitis	2 (11.8%)	2 (11.8%)	0
Hand and foot syndrome	1 (5.9%)	1 (5.9%)	0
Blood and lymphatic system disorders			
Anaemia	6 (35.3%)	6 (35.3%)	0
Neutropenia	4 (23.5%)	1 (5.9%)	3 (17.6%)
Thrombopenia	6 (35.3%)	6 (35.3%)	0
Liver function test disorders			
Gamma GT increased	2 (11.8%)	2 (11.8%)	0
Alanine aminotransferase increased	1 (5.9%)	1 (5.9%)	0
PAL increased	4 (23.5%)	4 (23.5%)	0
Aspartate aminotransferase increased	1 (5.9%)	1 (5.9%)	0
Hyperbilirubinemia	2 (11.8%)	2 (11.8%)	0
Neuromuscular disorders			
Myalgia	2 (11.8%)	2 (11.8%)	0

Additionally, as previous studies have shown that IL-1 inhibition may influence lean body mass parameter,^18,19^ we assessed the evolution of this parameter alongside weight during treatment. No significant changes were observed in either lean body mass or weight over the course of treatment, regardless of the administered XB2001 dose (Supplementary Fig. 1A–D).

### Clinical activity

**Fig. 1:**
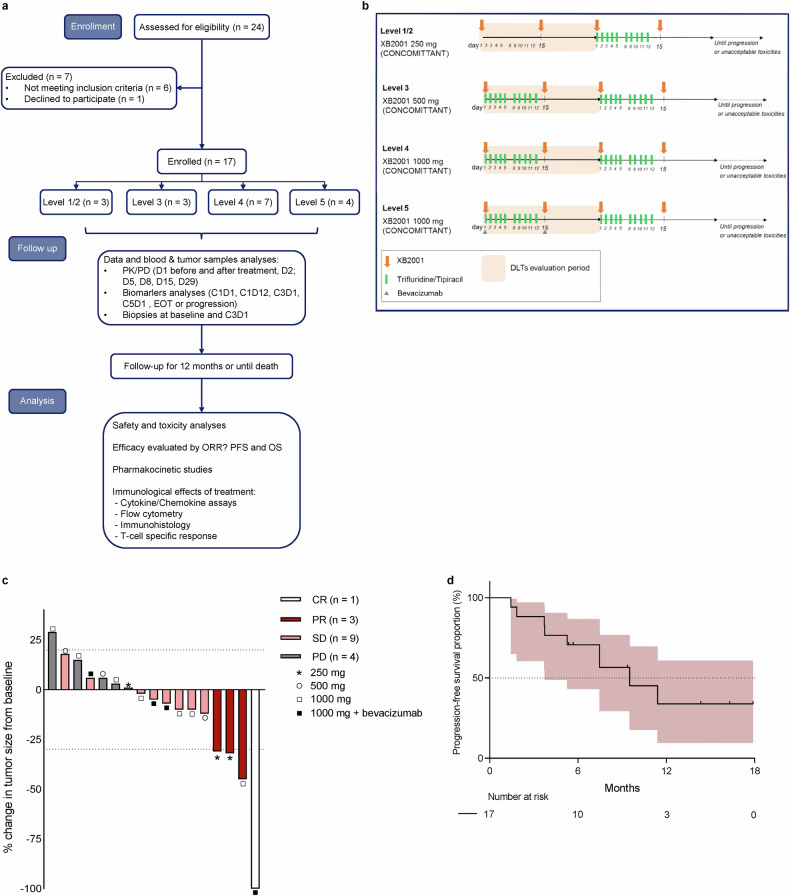
Clinical activity of XB2001. **a** Flow chart of the study. **b** Schematic diagram showing each dose level and associated treatments. **c** Waterfall plot of best sum of diameters percentage change for target lesions (*n* = 17) coloured according to RECIST criterion. The different dose levels are indicated above each bar with a symbol (* = 250 mg; ○ = 500 mg; □ = 1000 mg; ■ = 1000 mg + bevacizumab) (CR complete response, PR partial response, SD stable disease, PD progression disease). **d** Kaplan–Meier curves of progression-free survival of the study population with the median duration of response (*n* = 17). Survival distributions were compared using the log-rank test (**d**)

Clinical efficacy was assessed in the 17 patients who completed treatment and had at least one initial CT scan of evaluation (Fig. 1a, b). A total of 4 patients achieved partial response (PR), 9 patients reached stable disease (SD) and 4 patients progressive disease (PD), giving a disease control rate of 76%. A waterfall plot shows tumour regression after CT-scan assessment (Fig. 1c). At the 250 mg level, 2 out of 3 patients are still on treatment for up to 16 months; at the 500 mg level, 1 out of 3 patients is still on treatment for up to 12 months; at the 1000 mg level, 3 out of 7 patients are still on treatment for up to 10 months; and at the 1000 mg plus bevacizumab level, all 4 patients included are still on treatment for up to 6 months. Median PFS was 9.4 months for all patients included (95% CI, 3.6-Not reached; Fig. 1d). The CarcinoEmbryonic Antigen (CEA) level was evaluated at baseline and after 4 months of treatment in 13 patients and decreased in 8 out of 13 patients (median follow up: 14.1 months).

### Pharmacokinetic studies

**Fig. 2:**
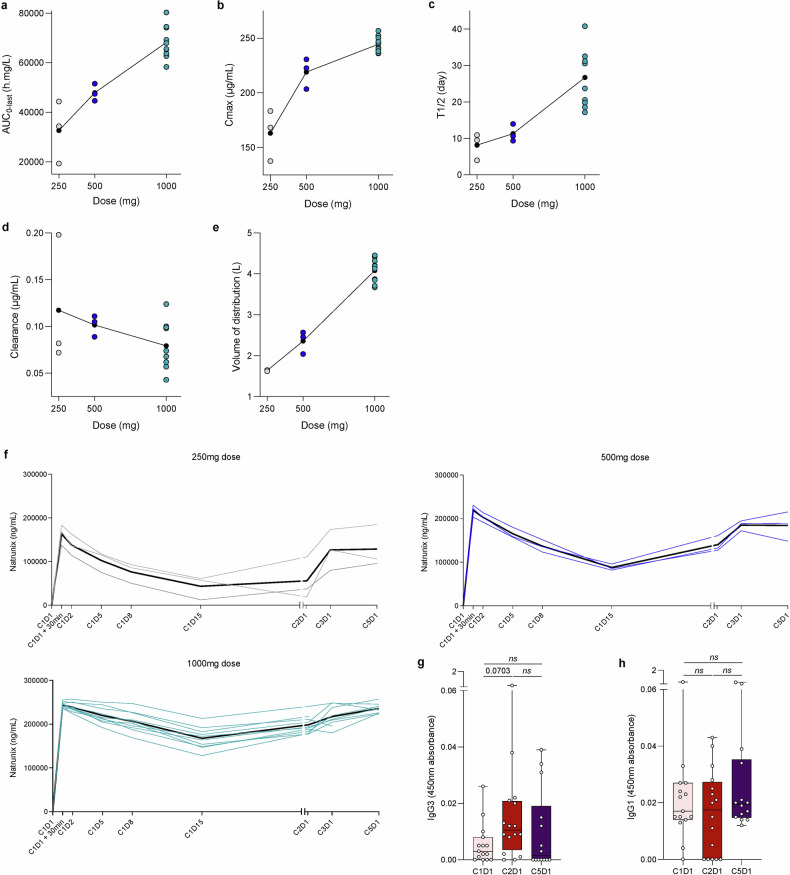
Pharmacokinetic analysis. **a**–**e** Dose-dependent pharmacokinetic measures of XB2001. Area under the curve (**a**), Cmax (**b**), half-life (**c**), clearance (**d**) and volume of distribution (**e**) are presented for the doses 250, 500 and 1000 mg. **f** Evaluation of XB2001 concentration in patient plasma over the first 5 treatment cycles according to the dose given (250, 500 or 1000 mg of XB2001). Measurement of IgG1 (**g**) and IgG3 (**h**) anti-drug antibody concentrations in patient plasma at C1D1, C2D1 and C5D1. n.s, not significant, comparison using Wilcoxon matched-pairs test

Pharmacokinetic data for XB2001 were analysed for each dose level. Figure 2 shows the course of pharmacokinetics parameters according to dose (AUC, Cmax and half-life time, clearance and volume of distribution) (Fig. 2a–e). AUC increased proportionally with dose, but this was not the case for Cmax, where there is non-linearity with dose and a plateau, with comparable Cmax observed between the 500 mg and 1000 mg doses. Half-life (T1/2) increased almost exponentially with dose, resulting in a lower decrease in plasmatic concentration in patients taking 1000 mg than in patients taking 500 mg. These data suggest that the 1000 mg dose induces target saturation and a stable plasmatic concentration. Evaluation of the concentration during the first 5 cycles confirms that 1000 mg induced a stable plasma concentration (Fig. 2f). Drug clearance decreased slightly with increasing dose, while there was a linear increase in volume of distribution with a peak at 4 L, which appears to represent classical distribution in the vascular and interstitial compartments (Fig. 2d, e). No relation was observed between pharmacokinetics parameters and clinical outcomes (Supplementary Tables 4 and 5). The coefficient of variation between patients was consistent with previous reports of therapeutic antibodies. Very small concentrations of anti-drug antibodies were detected in 3 patients after 2 and 5 cycles. These antibodies were predominantly IgG1 or IgG3 isotypes (Fig. 2g, h and Supplementary Fig. 2C).

### Correlative studies

#### Cytokines plasmic levels

**Fig. 3:**
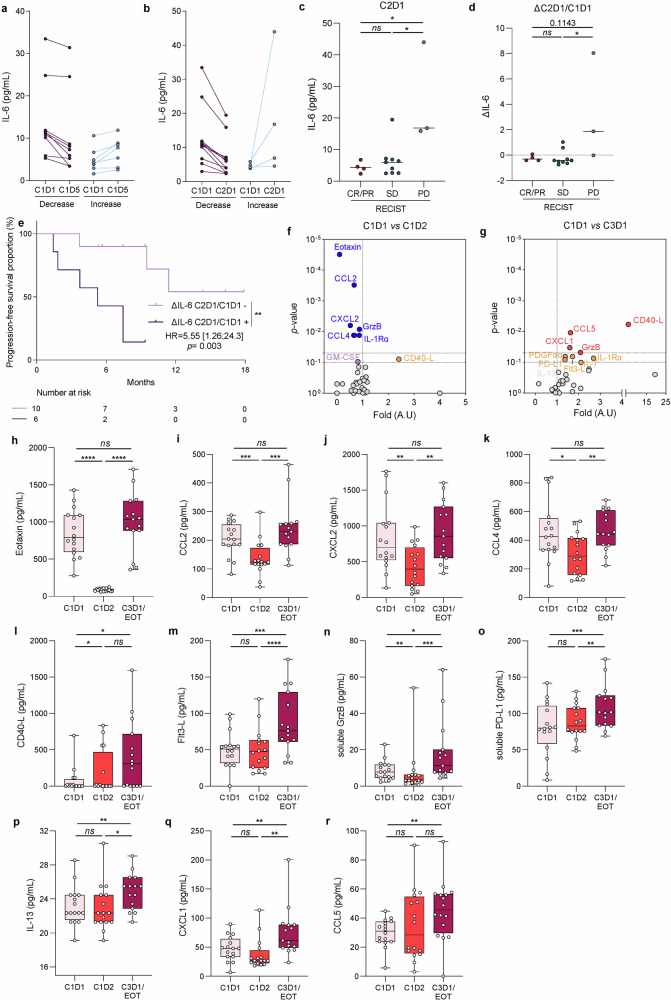
Correlative studies on patient plasma. Symbol and lines graph representing the concentration of IL-6 at C1D1 and C1D5 (**a**) or at C1D1 and C2D1 (**b**) according to the decrease or increase in tumour size after the first scan (*n* = 16). **c** Dot plot of IL-6 concentration in plasma of C2D1 patients according to RECIST response (CR complete response, PR partial response, SD stable disease, PD progression disease) (*n* = 16). n.s. not significant, *p < 0.05; comparison using Mann–Whitney unpaired test. **d** Dot plot of delta IL-6 concentration between C1D1 and C2D1 according to RECIST response (CR complete response, PR partial response, SD stable disease, PD progression disease) (*n* = 16). n.s. not significant, *p < 0.05; comparison using Mann–Whitney unpaired test. **e** Kaplan–Meier curves for progression-free survival with patients stratified according to the positive or negative delta of IL-6 concentration between C1D1 and C2D1. Two-sided *P* value with significance level set at 0.05. **f**, **g** Patient plasma was recovered at C1D1, C1D2 and C3D1 and cytokine and chemokine secretion was analysed by bioplex. Volcano plot describing differential analysis performed on bioplex data between C1D1 and C1D2 (**f**) and C1D1 and C3D1 (**g**) timepoints. The log2 FC indicates the mean expression level for each gene. Each dot represents one analysed target. **h**–**r** Patient plasma was recovered at C1D1, C1D2 and C3D1 and cytokine and chemokine secretion was analysed by bioplex. Box and whisker plots showing the concentration of Eotaxin (**h**), CCL2 (**i**), CXCL2 (**j**), CCL4 (**k**), CD40-L (**l**), Flt3-L (**m**), soluble Granzyme B (**n**), soluble PD-L1 (**o**), IL-13 (**p**), CXCL1 (**q**) and CCL5 (**r**) in patient plasma. n.s. not significant, *p < 0.05, **p < 0.01, ***p < 0.001, and ****p < 0.0001; comparison using Wilcoxon matched-pairs test. Survival distributions were compared using the log-rank test (**e**)

IL-6 is known to be induced by IL-1 and the decrease in serum of IL-6 levels has already been reported as a surrogate marker of the biological effect of IL-1 mAb.^20,21^ We assessed serum IL-6 levels at baseline (Cycle 1 Day 1) and during treatment. We observed a decrease in serum IL-6 levels in 9 of 16 patients at Cycle 1 Day 5 and in 12 of 16 patients at Cycle 2 Day 1 (Fig. 3a, b). Serum IL-6 levels at Cycle 2 Day 1 were lower in patients whose tumours had disease control by CT scan evaluation (Fig. 3c). Similarly, the decrease in serum IL-6 levels between baseline and Cycle 2 Day 1 was associated with disease control rate (DCR), and also with an improvement in PFS (Fig. 3d, e).

To further explore the biological effect of IL-1α mAb, we performed a wider analysis of plasma cytokines using a bioplex assay testing 46 different cytokines. Rapidly, one day after the first injection, we observed a significant decrease of eotaxin, CCL2, CCL4, Granzyme B, IL1Rα and CXCL2, on Cycle 1 Day 2, but this decrease was not persistent over time (Fig. 3f, h, l). In contrast, treatment induced an increase in soluble CD40-L, Granzyme B, CXCL1 and CCL5 at Cycle 3 Day 1 (Fig. 3g, m–r). However, none of these parameters was associated with clinical efficacy.

These data underline the biological efficacy of XB2001 by its ability to reduce IL-6 and other IL-1 related cytokines such as eotaxin, CCL2, CCL4 and CXCL2. The accumulation of soluble CD40L, and soluble Granzyme B might suggest the activation of an adaptive immune response. The decrease in plasma IL-6 concentration is a surrogate marker of the clinical efficacy of the experimental regimen.

#### Peripheral blood immune response

**Fig. 4:**
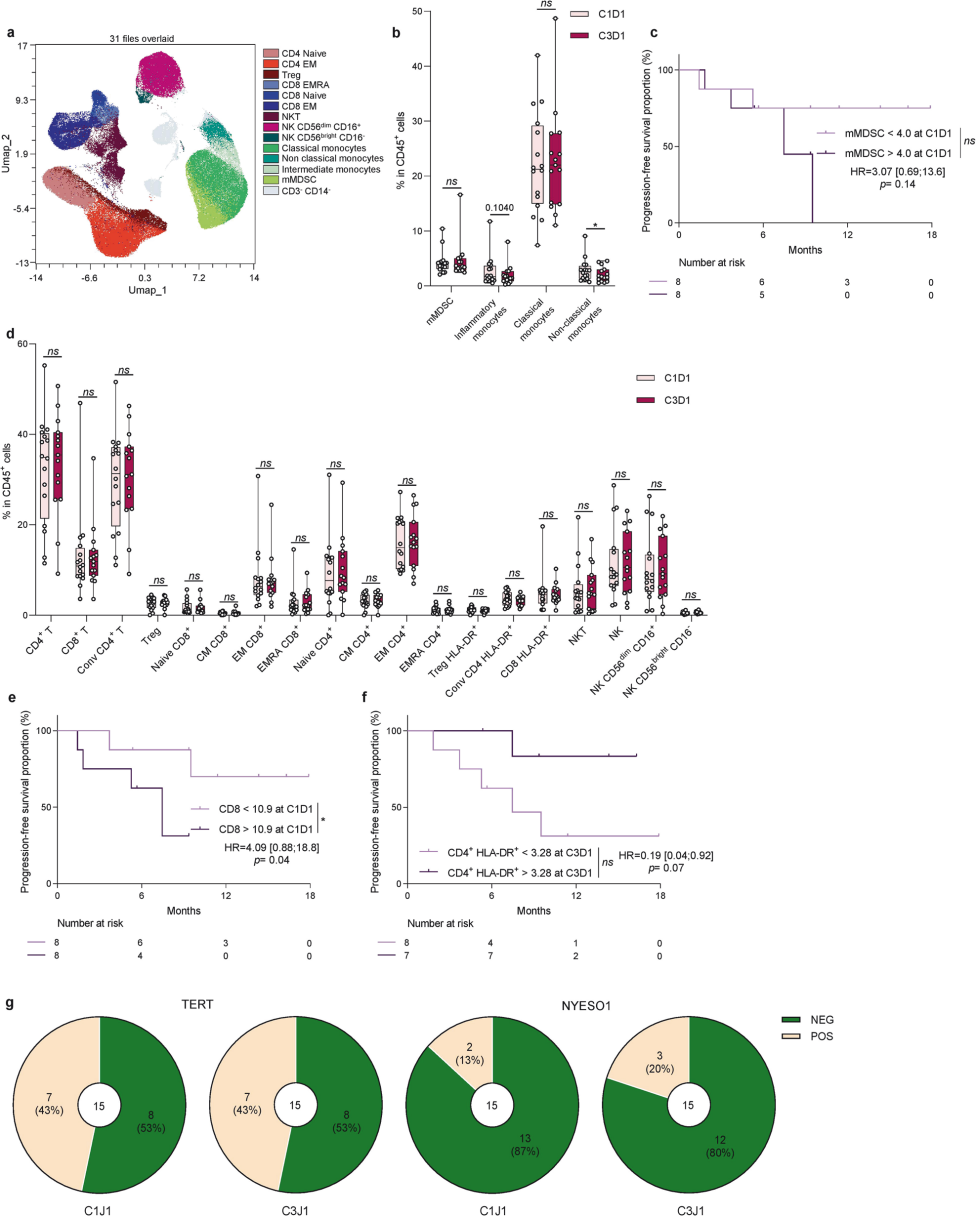
Correlative studies on peripheral blood immune response. **a** UMAP visualisation of CD45^+^ immune cells and CD45^+^ cells were clustered on the basis of their high-dimensional expression profile using Cluster X. Each dot corresponds to a single cell. Representative data from 16 patients, for whom PBMC analysis was performed at C1D1 and C3D1 (C3D1 missing for one patient); subsampled up to 5000 per sample; analysis of 155,000 cells in total. **b** Box and whisker plots showing the percentage of mMDSC, inflammatory monocytes, classical monocytes and non-classical monocytes in CD45^+^ cells at C1D1 (*n* = 16) and C3D1 (*n* = 15). n.s, not significant, *p < 0.05; comparison using Wilcoxon matched-pairs test. **c**, Kaplan–Meier curves for progression-free survival with patients stratified according to mMDSC percentage at C1D1 with a cutoff at the median. Two-sided *P* value with significance level set at 0.05. **d** Box and whisker plots showing the percentage of CD4^+^ T, CD8^+^ T, conventional CD4^+^ T, Treg, naïve, central memory (CM), effector memory (EM), effector memory CD45RA^+^ (EMRA) CD8^+^ or CD4^+^ T, Treg HLA-DR^+^, conventional CD4^+^ HLA-DR^+^, CD8^+^ HLA-DR^+^, NKT, NK, NK CD56^dim^ CD16^+^ and NK CD56^bright^ CD16^+^ cells in CD45^+^ cells at C1D1 (*n* = 16) and C3D1 (*n* = 15). n.s. not significant; comparison using Wilcoxon matched-pairs test. **e** Kaplan–Meier curves for progression-free survival with patients stratified according to CD8^+^ T cells percentage at C1D1 with a cutoff at the median. Two-sided *P* value with significance level set at 0.05. **f** Kaplan–Meier curves for progression-free survival with patients stratified according to CD4^+^ HLA-DR^+^ T cells percentage at C3D1 with a cutoff at the median. Two-sided *P* value with significance level set at 0.05. **g** Parts of whole (red and blue) showing the percentage of positive (in red) or negative (in blue) antitumor responses against TERT or NY-ESO1 at C1D1 and C3J1 (*n* = 15). Survival distributions were compared using the log-rank test (**c**, **e**, **f**)

We performed flow cytometry analysis on PBMCs from all patients with samples available at baseline, at Cycle 3 Day 1; we distinguished a total of 14 immune cell types (Fig. 4a and Supplementary Fig. 3). We observed a significant decrease in non-classical monocytes and a non-significant decrease in inflammatory monocytes with no modification of other myeloid subsets (Fig. 4b). When looking at the prognostic role of blood myeloid cells, we observed that the number of mMDSC at baseline was not significantly associated with poor PFS (Fig. 4c). No myeloid variable was prognostic at Cycle 3 Day 1.

When looking at blood lymphoid cells, we observed no change in the proportion of each of the cell subsets (Fig. 4d). However, we noted that a high baseline level of total CD8 T cells was associated with a lower PFS (Fig. 4e). At Cycle 3 Day 1, we noted that a high level of CD4 + HLA-DR+ cells was associated with longer PFS (Fig. 4f).

Specific anti-tumour T cell responses present in PBMC against shared tumour antigens like telomerase and NY-ESO1 was tested using ELISPOT. We observed that 7 and 2 out of 15 patients presented a baseline immune response against telomerase and NY-ESO1 respectively (Fig. 4g). During treatment, we did not observe induction of immune response against these antigens (Fig. 4g).

#### Immunohistology

**Fig. 5:**
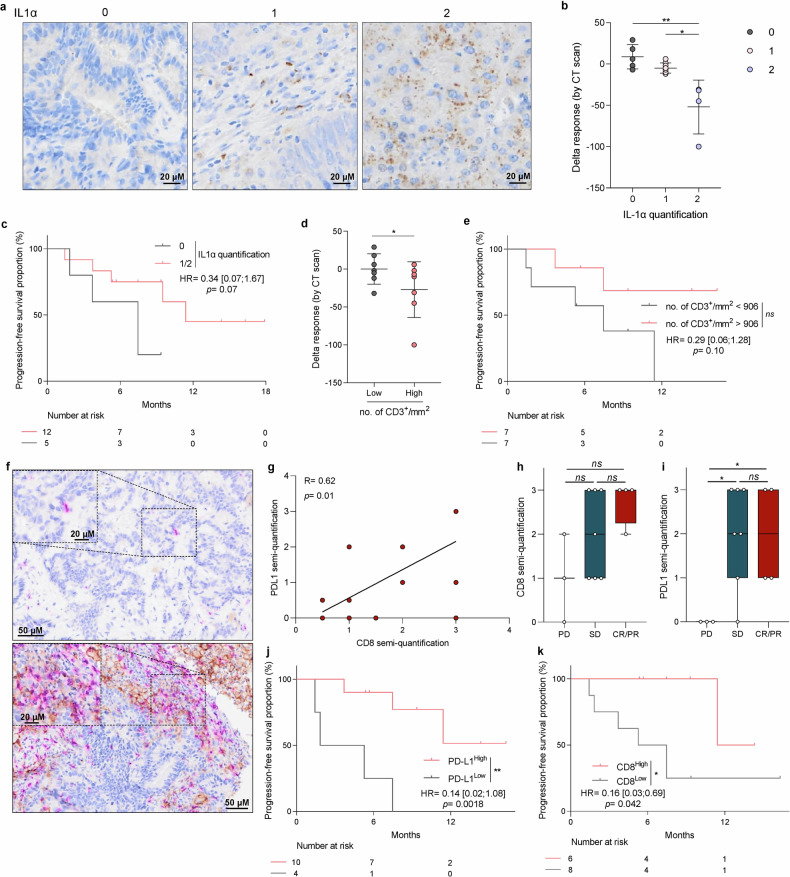
Correlative studies on tumour tissue. **a** Representative pictures of IL-1α staining of baseline colorectal cancer samples according to each intensity staining group: 0 (negative), 1 (low) and 2 (high) (scale bar 20 µM). **b** Dot plot of delta response by CT scan according to IL-1α quantification on baseline tumour samples (*n* = 17). *p < 0.05, **p < 0.01; comparison using two-way ANOVA test. **c** Kaplan–Meier curves for progression-free survival with patients stratified according to IL-1α quantification with IL-1α expression (1/2) or non-expression (0) as cutoff. Two-sided *P* value with significance level set at 0.05. **d** Dot plot of delta response by CT scan according to the number of CD3 per mm² on baseline tumour samples (*n* = 14). Patients were divided into two groups: low or high number of CD3 per mm². *p < 0.05; comparison using two-way ANOVA test. **e** Kaplan–Meier curves for progression-free survival with patients stratified according to the number of CD3 per mm² on baseline tumour samples with a cutoff at the median. Two-sided *P* value with significance level set at 0.05. **f** Representative pictures of CD8/PD-L1 double staining of baseline tumour samples from a patient with high expression and a patient with low expression of both markers (scale bar 50 µM) focusing on one area for each patient (scale bar 20 µM). **g** The correlation between CD8 semi-quantification and PD-L1 semi-quantification was determined (*n* = 14). Correlation was performed using the Pearson test. Box and whisker plots showing the CD8 semi-quantification (**h**) or the PD-L1 semi-quantification (**i**) on baseline tumour samples according to RECIST response (*n* = 14) (CR complete response, PR partial response, SD stable disease, PD progression disease). n.s, not significant, *p < 0.05; comparison using Mann–Whitney unpaired test. Kaplan–Meier curves for progression-free survival with patients stratified according to PD-L1 quantification (**j**) or CD8 quantification (**k**) on baseline tumour samples with a cutoff at the median. Two-sided *P* value with significance level set at 0.05. Survival distributions were compared using the log-rank test (**c**, **e**, **j**, **k**)

A baseline sample was obtained from 17 patients, and we were able to perform IL-1α labelling on a tumour slide taken at inclusion. We observed that IL-1α was mainly expressed in cells of myeloid morphology or in cancer cells (Fig. 5a). IL-1α was not expressed in 5 samples, while it was moderately or intensely expressed in other samples. IL-1α expression is associated with better response to treatment in terms of objective radiologic response and PFS (Fig. 5b, c). The mean number of CD3 per mm² was 954.8 [115.9 – 3041.4]. A high CD3 count was associated with better objective radiological response and improved PFS (Fig. 5d, e). Double CD8/PD-L1 staining was also performed on baseline slides (Fig. 5f). We observed a strong correlation between PD-L1 and CD8 expression (Fig. 5g). Patients with high PD-L1 and CD8 expression had better ORR and better PFS than other patients (Fig. 5h–k). Supplementary Table 6 presents univariate and multivariate Cox regression models for each of the tested biomarkers.

These data show that the immune context predicts response to experimental treatment.

## Discussion

This study represents the first clinical results for the usage of XB2001, an IL-1α-targeting monoclonal antibody (mAb), in humans, addressing the critical need for effective treatments in metastatic colorectal cancer (mCRC), particularly in chemo-resistant cases. IL-1α plays a capital role in CRC and more specifically in the tumour microenvironment (TME), promoting angiogenesis, immune evasion, and tumour progression. Current third-line treatment options, such as trifluridine/tipiracil (FTD/TPI) and bevacizumab, have shown limited efficacy in heavily pretreated patients.^7^ Our study demonstrates the potential of XB2001 to enhance the efficacy of this standard regimen, offering a novel approach to overcoming the challenges of chemoresistance while maintaining a favourable safety profile.

The combination of XB2001 with FTD/TPI and bevacizumab showed encouraging clinical outcomes, with 76% of disease control rate and 9.5 months of PFS. These results surpass previous benchmarks set by regorafenib, fruquintinib, and FTD/TPI monotherapy in similar patient populations.^4–6,22^ A notable reduction in serum IL-6 levels, an IL-1-induced cytokine, was associated with improved therapeutic response, highlighting the on-target activity of XB2001. Additionally, pharmacokinetic data indicated dose-dependent saturation at 1000 mg, supporting its selection as the RP2D. Compared with historical clinical data, these findings suggest that the addition of XB2001 may significantly improve outcomes in mCRC patients with high IL-1α expression.

Despite promising results, this study has limitations. The small sample size (*n* = 17) and absence of a control arm constrain the generalizability and definitive attribution of outcomes to XB2001. The manageable treatment-related adverse events (TRAEs) observed, such as neutropenia and infections, should be further monitored in future trials to avoid unexpected rare toxicity. Furthermore, while post-hoc biomarker analyses suggest that high intratumoral IL-1α and immune infiltration (e.g., CD8/PD-L1 expression) predict better outcomes, these findings require validation in larger, randomized studies. The study’s phase I design inherently favours patients with better performance status, potentially biasing efficacy results.

This study opens avenues for future research on the immunomodulatory and antitumoral effects of XB2001. Preclinical data suggest that FTD/TPI enhances immune responses, including reducing immunosuppressive macrophages and triggering immunogenic cell death.^23^ The observed increase in immune markers such as serum granzyme B and CD40L highlights XB2001’s potential to synergize with FTD/TPI and bevacizumab by fostering an antitumoral immune environment. Another preclinical study in the same model showed that the combination of FTD/TPI with an anti-PD-1 antibody improved tumour regression compared with either agent alone, indicating synergistic activity of the combination in MSS CRC, and suggesting a potential immune effect of FTD/TPI in CRC.^24^ Furthermore, increased tumour immunogenicity is observed with a higher CD8/Treg ratio with the use of the combination. Our study confirms that initial immune infiltration of the tumour is associated with a better outcome. CD8 and PD-L1 expression are associated with better therapeutic outcomes. CD8 and CD3 immune infiltration are well-established prognostic parameters.^25,26^ Transcriptomic data from TCGA (Supplementary Fig. 4) and previous IHC-based studies^27^ have confirmed that PD-L1 expression is also linked to better prognosis in CRC, which is not surprising given the ability of IFNγ-producing CD8 T cells to promote PD-L1 expression. Thus, the association of clinical response with initial immune infiltration suggests potential for personalized approaches based on immune profiling. Future randomized trials should explore the role of XB2001 in combination with immune checkpoint inhibitors, aiming to enhance tumour immunogenicity further.

In conclusion, XB2001, combined with FTD/TPI and bevacizumab, demonstrated a favourable safety profile and promising efficacy in the third-line treatment of mCRC. The drug effectively reduces IL-1-inducible cytokines like IL-6, supporting its biological and clinical relevance. Biomarker analysis highlights the importance of targeting the inflammatory and immune components of the TME, with tumours expressing high IL-1α and immune infiltration showing the best responses. Despite the limitations of this early-phase study, our findings provide a strong rationale for advancing XB2001 to larger, randomized trials to compare its efficacy against established standards of care and evaluate its potential to transform treatment paradigms in mCRC.

### Statement of translational relevance

TASKIN is the first early-phase clinical trial testing the hypothesis of tolerance and efficacy of the association between trifluridine/tipiracil + bevacizumab and anti-IL-1α mAb immunotherapy for multi-treatment metastatic colorectal cancer (mCRC). With the growing interest in reversing resistance to immunotherapy in microsatellite stable colorectal cancer, TASKIN confirms the feasibility and sustainability of integrating immunotherapy with standard treatments. Phase 2 enrolment is currently ongoing, and will enable us to compare the effect of this combination with that of standard treatment.

## Materials and methods

### Patient selection

Patients aged 18 and over with histologically confirmed non-resectable metastatic colorectal cancer (mCRC) were eligible for inclusion. Patients were required to have received, and experienced progression or unacceptable adverse effects with standard chemotherapies regimens. Previous treatment must have included fluoropyrimidine, irinotecan, oxaliplatin, anti-VEGF monoclonal antibody (not limited to bevacizumab) or anti-EGFR (epidermal growth factor receptor) monoclonal antibody (for patients with *RAS* wild-type disease). Neoadjuvant or adjuvant chemotherapy was allowed if the disease recurred during treatment or within six months of the last dose. Additionally, MMR, BRAF, and RAS status had to be determined prior to enrolment. Eligible patients were required to have adequate organ function and an Eastern Cooperative Oncology Group (ECOG) performance-status score of 0 or 1. Organ function criteria included an absolute neutrophil count ≥1500 cells/mL, haemoglobin ≥9 g/dL, platelets ≥100,000/mL, creatinine ≤1.5 times the upper limit of normal (ULN) or creatinine clearance >50 mL/minute calculated by the Cockcroft-Gault equation, bilirubin ≤1.5 mg/dL or ≤2.0 ULN in the presence of liver metastases, and alanine transaminases ≤3 times ULN (or ≤5 times ULN in patients with liver metastases). The main exclusion criteria included high microsatellite instability/mismatch repair deficiency, *BRAF* V600 mutations and brain metastases.

### Ethics approval statements

The protocol was approved by the Ethics Committee “Comité de Protection des Personnes Sud-Ouest et Outre-Mer IV on under the 5 May 2022 number 22.00525.000075 and by the ANSM (MEDAECNAT-2022-05-0020 2022-001400-18) in accordance with French Law. The study was registered with ClinicalTrials.gov under the identifier NCT05201352. The study complied with the Declaration of Helsinki and Good Clinical Practice guidelines. Written informed consent was obtained from all patients prior to selection.

### Study design

The protocol of this phase I, open label, 3 + 3 dose escalation study has previously been described elsewhere.^28^ Briefly, the study aimed to evaluate the safety and tolerability and to establish the Maximum Tolerated Dose (MTD) of XB2001 in patients with mCRC receiving FTD/TPI-based chemotherapy (FTD/TPI 35 mg/m² on days 1–5 and 8–12 every 28 days.). Each patient in phase I part of this study was treated until disease progression or the onset of unacceptable toxicities. Three patients were treated at each dose level of XB2001. Level 1/2 involved XB2001 at a 250 mg dose for two doses alone, followed by concomitant administration of FTD/TPI plus XB2001 at the 250 mg dose (3 patients were treated at this level). Level 3 consisted of XB2001 at a 500 mg dose, combined with FTD/TPI (3 patients were treated at this level). Level 4 involved concomitant administration of XB2001 at a dose of 1000 mg plus FTD/TPI, with 6 patients were expected. However, an additional patient was added due to an error in the administration of XB2001, which was given at 500 mg instead of 1000 mg for patient 8, making it impossible to assess the dose-limiting toxicity (DLT) period. Finally, following the approval of concomitant administration of FTD/TPI and bevacizumab as a new standard of care, 4 additional patients were added (level 5) to assess the safety of FTD/TPI plus bevacizumab (5 mg/kg i.v. every 14 days) in combination with XB2001 at the 1000 mg dose. For each dose level, 3 patients were treated, and if there was no dose-limiting toxicities (DLTs) in the first cycle, the protocol moved to the next highest dose. If DLT occurred, a further 3 patients were treated at this dose. If ≥2 patients showed DLT, the dose was considered as not tolerated, and lower doses were studied in subsequent cohorts. At the highest dose, 6 patients were included. The MTD was defined as the highest dose achieved at which no more than one out of six patients developed DLT. The Recommended phase 2 dose (RP2D) was determined on the basis of safety, pharmacokinetics and preliminary efficacy assessments, in patients treated at doses authorized for safety.

Patients were included in the safety analysis if they received at least one dose of XB2001. Safety assessments encompassed physical examinations, laboratory tests, electrocardiograms, and monitoring of adverse events (AEs), which were graded using the NCI Common Terminology Criteria for Adverse Events (version 4.03). Investigators determined the relationship of each AE to the study drug as unrelated, unlikely, possibly, probably, or definitely related. DLTs were defined as grade 4 (G4) haematologic AEs or grade ≥3 non-haematologic AEs that were considered related to the study drug, but not to disease progression, and that did not resolve within 14 days following presentation with standard management. DLTs were evaluated during the first 28 days of treatment.

All patients received prophylactic G-CSF.

### Efficacy

CT scan imaging was obtained at screening and every 8 weeks during treatment for the first 12 months of treatment and then every 12 weeks, until end-of-treatment (EOT). Objective response was assessed using RECIST version 1.1 (RECIST v1.1) in patients who received at least one dose of XB2001. Progression-free survival (PFS) is defined as the time from inclusion in the clinical trial to disease progression or death from any cause.

### Plasma collection

Blood samples were collected from patients for pharmacokinetics analysis at the following time-points: pre-dose; and days 1, 2, 5, 8, 15, 28 post-dose. Blood samples were also collected from patients for biomarker analysis at the following time-points: Cycle 1 Day 1, Cycle 3 Day 1, Cycle 5 Day 1 and at the end of treatment. After the blood sampling was done at the different times described above, a heparin tube was used to isolate and bank the plasma. For this purpose, after collection, the heparin tube was centrifuged at 1000 g for 10 min at room temperature. The plasma was then recovered, aliquoted at a rate of 1000 µL per cryotube and stored at −80 °C until analysis.

### Peripheral blood mononuclear cell isolation

Using the blood samples for biomarker analysis taken on Cycle 1 Day 1, Cycle 3 Day 1, Cycle 5 Day 1 and at the end of treatment, after blood sampling in EDTA tubes, peripheral blood mononuclear cells (PBMC) were isolated from the whole blood by density gradient centrifugation (Lymphocyte Separation Medium, CMSMSL0101, Eurobio) with UNI-SEP tubes (U-16, Eurobio Scientific). Whole blood was transferred into UNI-SEP tubes at a volume of 17 mL per tube and centrifuged at 1000 g for 20 min, with an acceleration setting of 5 and no brake applied. Following the removal of as much plasma as possible, the phase containing enriched PBMCs was recovered. The cells were then washing twice with 45 mL of phosphate-buffered saline (PBS), and centrifuged at 300 g for 10 min. The resulting PBMC pellet was resuspended in 5 mL of PBS 1X for counting. A final wash with 5 mL of PBS 1X was performed before cryopreservation, which involved freezing at a concentration of 10.10^6^ cells per cryotube in a solution containing 50% CS5 Cryostor (C2874, Sigma-Aldrich) and 50% CS10 Cryostor (C2999, Sigma-Aldrich) until further use.

### Pharmacokinetic of XB2001

Blood samples were collected from patients for XB2001 pharmacokinetics analysis at the following time-points: pre-dose; and days 1, 2, 5, 8, 15, 28 post dose. Plasma concentrations of XB2001 were determined by ELISA, according to the procedure from XBiotech. Briefly, after coating of the capture antigen on a microplate for at least 1 hour at 37 °C and blocking of the capture antigen with 20% of FBS-TBS buffer for at least 1 hour at room temperature (RT), the standards, controls and samples were prepared and samples were diluted in a 20% FBS-TBS buffer in three independent dilutions (1/100 1/1000, 1/5000) and then transferred to the microplate and incubated at RT for approximately 2 h. After washing the plate 5 times with wash buffer, the secondary antibody mouse anti-human IgG4 Fc-HRP (0.5 mg/ml, Southern Biotech Product, Clone: HP6025) was transferred into the microplate and incubated at RT for approximately 1 h in the dark. Finally, after washing the plate 5 times with wash buffer, TMB substrate (421101, BioLegend) was added into each well and incubated at RT in the dark for 15 min. The reaction was stopped by adding stop solution (423001, BioLegend) to each well of the microplate and after gently vortexing the plate, the absorbance was read at 450 nm.

Non compartmental analysis (NCA) of XB2001 was performed using PKanalix software (MonolixSuite 2023R1, Lixoft, Antony, France) in order to calculate pharmacokinetics parameters, for each individual. The trapezoidal linear method was used to compute the area under the curve from time zero to the time of the last measured concentration (AUC_0-last_). This method only uses observed data during the first infusion, in order to calculate the slope of the terminal elimination phase and the corresponding terminal half-life (T_1/2_). In this case, it allows to compute specific pharmacokinetics parameters for intravenous administration, such as the clearance (Cl) and the volume of distribution (Vd). Maximum observed concentration (C_max_) was determined from the observed concentration. AUC_0-last_ was computed rather than AUC_0-inf_ because the percentage of AUC_0-inf_ due to extrapolation from the last time point to infinity was too high (>20). Default software settings were used unless otherwise stated.

### Anti-product response analysis

The anti-product response to XB2001 was assessed by ELISA assay according to XBiotech’s procedure and carried out at the following sample times: pre-dose; and days 1, 2, 5, 8, 15, 28, 56, 112 post dose. Briefly, a dilution of the capture antibody (XB2001) or coating buffer alone were added on each well of the microplates and plates were incubated at 37 °C for 1 h. After blocking and washing of microplates, plasma samples were diluted 1/500 with 20% of FBS-TBS solution and then were added in each well (coated or non-coated with Natrunix) and incubated at RT for 2 h. During incubation, the dilution of each secondary antibody (mouse anti-human IgG2 Fc-HRP conjugated (9060-05, Southern Biotech, Clone: 31-7-4), mouse anti-human IgG3 (hinge region specific)-HRP conjugated (9230-05, Southern Biotech, Clone: SB81a), mouse anti-human IgG1 (Fc specific)-HRP conjugated (9054-05, Southern Biotech, Clone: HP6001), goat anti-human IgA (a-chain specific)-HRP conjugated (2050-05, Southern Biotech), mouse anti-human IgM (μ-chain specific)-HRP conjugated (9040-05, Southern Biotech, Clone: JDC-10)) was prepared in 20% FBS-TBS buffer. After washing the microplates, the different secondary antibodies were added to each well of interest in microplates coated and non-coated with XB2001 and were incubated at RT in the dark for 1 h. After washing the microplates, TMB substrate was added into each well and incubated at RT in the dark for 15 min. The reaction was stopped by adding stop solution to each well of the microplate and after gently vortexing the plate, the absorbance was read at 450 nm.

Pharmacodynamics: The following sampling times were analysed: pre-dose, and days 5 and 28 post dose. Plasma IL-6 concentrations were determined using ELISA, following the manufacturer’s guidelines (Biolegend, 430517).

### Histology

Biopsies were obtained either prior to study entry using archival materials less than 2 years old or at baseline as fresh samples. Fresh biopsies were fixed in PFA immediately after collection and embedded in paraffin by the pathology laboratory. Four-micron slices were prepared from FFPE tumour samples using a Tissue-Tek Autosection microtome (Sakura). Slide staining was performedon an Autostainer 48 (Agilent) using primary antibodies against IL1α primary antibody (clone BSB-138, Diagomics) or CD3 (clone F7.2.38, Agilent). Slides were deparaffinized in a pH9 buffer at 95 °C for 25 min, then cooling and washed twice in wash buffer (Agilent) for 5 min each. Peroxydase activity was blocked with peroxydase blocking reagent (SM801, Agilent). Anti-human CD3 or anti-human IL1α antibodies were applied for 30 min (CD3) or 60 min (IL1α) at room temperature. After two washes, EnVision FLEX HRP polymers (SM802, Agilent) were added for 20 min (CD3) or 30 min (IL1α) at room temperature. DAB (SM803, Agilent) was applied for 5 min (CD3) or 10 min (IL1α), followed by two washes. Finally, slides were stained with hematoxylin (SM806, Agilent) for 5 min and mounted using a Leica automated coverslipper. The slides were then digitalised at x20 magnification using a VS200 scanner (Olympus) to generate whole slide imaging (WSI) files in vsi format. The analysis was performed with QuPath software (v.5.1), and ROIs were selected on each slide.

For CD3 analysis, the number of positive cells was counted in each area and the average was determined. IL-1α staining was evaluated independently by two pathologists and split into three groups, namely IL-1α negative, IL-1α low and IL-1α high.

The immunoscore for CD8/PDL1 was assessed by measuring the densities of PD-L1+ and CD8+ cells and evaluating their spatial proximity within a single tissue section. Immunohistochemistry was performed using anti-PD-L1 (QR1), anti-CD8 (C8/114B) antibodies, followed by hematoxylin counterstaining. PD-L1 and CD8 antibodies were visualized using DAB and Magenta-HRP, respectively. All stained slides were digitized at 20x magnification using a high-resolution scanner (VS200, Olympus). The resulting whole-slide images were analysed using QuPath software.

### Bioplex assay

Forty-five analytes were quantified in plasma at two timepoints: Cycle 1 Day 1 and Cycle 3 Day 1, using the Human XL Cytokine Magnetic 45-plex Luminex® assay (#898855, R&D Systems, USA) according to the manufacturer’s protocol. These analytes included: C-C motif chemokine ligand (CCL)2, 3, 4, 5, 11, 19, and 20, CD40 ligand, fractalkine, C-X-C motif chemokine ligand (CXCL) 1, 2, and 10, epidermal growth factor (EGF), fibroblast growth factor (FGF), FMS-like tyrosine kinase 3 ligand (FLT3L), granulocyte colony-stimulating factor (G-CSF), granulocyte-macrophage colony-stimulating factor (GM-CSF), granzyme B, interferon (IFN)-α, -β, -γ, interleukin (IL)-1α, 1β, 1RA, 2, 3, 4, 5, 6, 7, 8, 10, 12, 13, 15, 17A, 17E, and 33, programmed death-ligand 1 (PD-L1), platelet-derived growth factor (PDGF)-AA and AB/BB, transforming growth factor (TGF)-α, tumour necrosis factor (TNF)-α, TNF-related apoptosis inducing ligand (TRAIL), and vascular endothelial growth factor (VEGF). The standard performance values for each analyte are provided in Supplementary Table 7.

### Immune cells analysis

We performed immunophenotyping by flow cytometry on PBMCs from Cycle 1 Day 1 and Cycle 3 Day 1.

#### Blood count analysis

Antibodies used: Multicolour flow cytometry was performed using custom-designed tubes from Beckman Coulter incorporating dry coating technology with the following antibodies: anti-CD16-FITC (clone 3G8), anti-CD56-PE (clone N901), anti-CD25-ECD (clone B1.49.9), anti-HLA-DR-PE-Cy5.5 (clone Immu-357), anti-CD14-PE-Cy7 (clone RMO52), anti-CD4-APC (clone 13B8.2), anti-CD8-AlexaFluor700 (Clone B9.11), anti-CD3-APC-AlexaFluor750 (clone UCTH1), anti-CD15-PacificBlue (clone 80H5) and anti-CD45-KromeOrange (clone J.33). Liquid antibodies were also used: anti-CD127-BV605 (BioLegend, clone A019D5), anti-CCR7-BV650 (BioLegend, clone G043H7) and anti-CD45RA-BV785 (BioLegend, clone HI100).

Staining protocol: After thawing and counting, 1.10^6^ of PBMCs were added to DURAClone tubes containing liquid antibodies, vortexed for 15 s and incubated at room temperature in the dark for 15 min. Next, 2 mL of red blood lysis solution (VersaLyse solution, A09777, Beckman Coulter) mixed with 50 μL of IOTest 3 Fixative solution (A07800, Beckman Coulter) was added, inverted and incubated for an additional 15 min in the dark. Following incubation, the cells were centrifuged at 1000 rpm for 5 min. The pellet was washed with 2 mL PBS, centrifuged again at 1000 rpm for 5 min and resuspended in 150 µL PBS. Finally, cell acquisition was performed on a DxFLEX cytometer (Beckman Coulter).

#### Data analysis

FCS files were analysed by commercial software Kaluza (Beckman Coulter) and Omiq (Omiq, Inc.).

Manual gating: After checking and validating the compensations for each FCS file in Kaluza software (Beckman Coulter), live singlet CD45^+^ cells were exported for analysis on a pipeline implemented in Omiq.

High-dimensional data analysis was performed on 31 samples (*n* = 16 samples at Cycle 1 Day 1 and *n* = 15 samples at Cycle 3 Day 1) and 5 000 cells per sample were included (total *n* = 155,000 CD45^+^ immune cells).

Uniform Manifold Approximation and Projection (UMAP) analysis: Visualization of the global single-cell landscape was performed using UMAP in Omiq based on channels CD16, CD56, CD25, HLA-DR, CD14, CD4, CD8, CD3, CD127, CCR7 and CD45RA, random seed 4478, epochs 200, learning rate 1, neighbours 75 and minimum distance 0,4. Then ClusterX was performed based on the umap-1 and umap-2 features, and by using a Gaussian Kernel with an alpha coefficient of 0.001.

### T cell specific immune response

Circulating tumour-specific T cell responses were evaluated using an IFN-γ ELISPOT assay after short-term in vitro stimulation of PBMCs. Cells were stimulated with a mixture of eight TERT-derived major histocompatibility complex class II‒binding peptides (a pool of HLA-DR and HLA-DP-restricted TERT peptides^29,30^) and NY-ESO1 peptides at a concentration of 5 μg/mL for six days, as previously described.^29,31^ All synthetic peptides (>90% purity) were obtained from JPT (Germany). To asses antiviral recall responses, a peptide mixture referred to as CEF – comprising peptides from influenza virus (Flu), Epstein–Barr virus, cytomegalovirus (Cellular Technology Ltd) was used. Frozen PBMCs were thawed and cultured with tumour-derived peptides (5 µg/ml) in RPMI supplemented with 5% fetal bovine serum in 24-well plates (4 × 10^6^ cells per well). IL-7 (5 ng/mL, 200-07, PeproTech) and IL-2 (20 UI/mL, 202-IL-010, Novartis) were added on days 1 and 3, respectively. On day 7, the antigen-specific T cells was quantified using the IFN-γ ELISpot assay following the manufacturer’s instructions (hIFNgp-2M/5, Human IFN-γ Single-Color ELISPOT Precoated 96 wells Strip, ImmunoSpot). Briefly, lymphocytes from the in vitro stimulation (10^5^ cells per well) were incubated for 24 h at 37 °C in ELISpot plates precoated with anti-human IFN-γ antibodies, in the presence or absence of peptide mixtures, using CTL-Test™ PLUS Medium (CTLTP-010, ImmunoSpot). Controls included cells cultured with medium alone (negative control) and phorbol 12-myristate 13-acetate (1 ng/mL; P8139, Sigma-Aldrich)/ionomycin (10 mmol/L; I3909, Sigma-Aldrich) as positive control. IFN-γ spots were revealed as per the manufacturer’s protocol (Immunospot, CTL, Germany). The number of specific T cells, expressed as ΔIFN-γ spots per 10^5^ cells, was calculated by subtracting the background value (medium control). Spot-forming cells were quantified using the C.T.L. Immunospot system (Cellular Technology Ltd). Responses were deemed positive if the number of IFN-γ spots exceeded 10 and was at least twice the background level.^32^

### Correlative studies

We examined correlations in immunohistological analysis with analysis of IL1α expression, CD3 content and Immunoscore IC, serum analysis with assessment of cytokine changes using bioplex assay, evolution of immune cell numbers in blood using multiparametric flow cytometry and study of telomerase-specific T-cell response using ELISPOT.

### Statistical analysis

Patient characteristics and AEs were summarized using descriptive statistics. The safety analysis included all enroled patients who received at least one dose of XB2001, while efficacy and biomarker analyses included patients who completed at least one treatment cycle. Comparisons of patient characteristics were conducted using the Mann–Whitney unpaired test for continuous variables, as applicable. The prognostic value of variables for PFS was assessed using univariate Cox regression models. Continuous variables were dichotomized based on either the median cut-off or the methodology proposed by Lausen et al.^33^, utilizing the *maxstat* R library. Survival probabilities were estimated using the Kaplan–Meier method, and survival curves were compared with the log-rank test. All statistical analyses were performed using R software (version 4.2.3) and GraphPad Prism (version 10.0.02). A *p*-value < 0.05 was considered statistically significant.

## Supplementary information

Supplemental Material

Protocol of the clinical trial TASKIN

## Data Availability

This study was approved by the Ethics Committee “Comité de Protection des Personnes Sud-Ouest et Outre-Mer IV on May 5, 2022 under reference number 22.00525.000075, and by the ANSM (MEDAECNAT-2022-05-0020 2022-001400-18) in accordance with French Law. The datasets generated during this study are available upon request from the corresponding authors.

## References

[1] Sung, H. et al. Global Cancer Statistics 2020: GLOBOCAN Estimates of Incidence and Mortality Worldwide for 36 Cancers in 185 Countries. *CA Cancer J. Clin.* **71**, 209–249 (2021). 33538338 10.3322/caac.21660

[2] Cervantes, A. et al. Metastatic colorectal cancer: ESMO Clinical Practice Guideline for diagnosis, treatment and follow-up☆. *Ann. Oncol.* **34**, 10–32 (2023). 36307056 10.1016/j.annonc.2022.10.003

[3] André, T. et al. Pembrolizumab in Microsatellite-Instability–High Advanced Colorectal Cancer. *N. Engl. J. Med.* **383**, 2207–2218 (2020). 33264544 10.1056/NEJMoa2017699

[4] Grothey, A. et al. Regorafenib monotherapy for previously treated metastatic colorectal cancer (CORRECT): an international, multicentre, randomised, placebo-controlled, phase 3 trial. *Lancet* **381**, 303–312 (2013). 23177514 10.1016/S0140-6736(12)61900-X

[5] Mayer Robert, J. et al. Randomized Trial of TAS-102 for Refractory Metastatic Colorectal Cancer. *N. Engl. J. Med.* **372**, 1909–1919 (2015). 25970050 10.1056/NEJMoa1414325

[6] Dasari, A. et al. Fruquintinib versus placebo in patients with refractory metastatic colorectal cancer (FRESCO-2): an international, multicentre, randomised, double-blind, phase 3 study. *Lancet* **402**, 41–53 (2023). 37331369 10.1016/S0140-6736(23)00772-9

[7] Prager, G. W. et al. Trifluridine–Tipiracil and Bevacizumab in Refractory Metastatic Colorectal Cancer. *N. Engl. J. Med.* **388**, 1657–1667 (2023). 37133585 10.1056/NEJMoa2214963

[8] Grivennikov, S. I., Greten, F. R. & Karin, M. Immunity, Inflammation, and Cancer. *Cell* **140**, 883–899 (2010). 20303878 10.1016/j.cell.2010.01.025 PMC2866629

[9] Beaugerie, L. Inflammatory bowel disease therapies and cancer risk: where are we and where are we going? *Gut* **61**, 476–483 (2012). 22157331 10.1136/gutjnl-2011-301133

[10] Lurje, G. et al. Polymorphisms in interleukin 1 beta and interleukin 1 receptor antagonist associated with tumor recurrence in stage II colon cancer. *Pharmacogenet. Genomics* **19**, 95 (2009). 18987561 10.1097/FPC.0b013e32831a9ad1

[11] Apte, R. N. et al. The involvement of IL-1 in tumorigenesis, tumor invasiveness, metastasis and tumor-host interactions. *Cancer Metastasis Rev.* **25**, 387–408 (2006). 17043764 10.1007/s10555-006-9004-4

[12] Voronov, E. et al. IL-1 is required for tumor invasiveness and angiogenesis. *Proc. Natl Acad. Sci.* **100**, 2645–2650 (2003). 12598651 10.1073/pnas.0437939100 PMC151394

[13] Hurtado, C. G., Wan, F., Housseau, F. & Sears, C. L. Roles for Interleukin 17 and Adaptive Immunity in Pathogenesis of Colorectal Cancer. *Gastroenterology* **155**, 1706–1715 (2018). 30218667 10.1053/j.gastro.2018.08.056 PMC6441974

[14] Liu, C. et al. Blocking IL-17A enhances tumor response to anti-PD-1 immunotherapy in microsatellite stable colorectal cancer. *J. Immunother. Cancer* **9**, e001895 (2021). 33462141 10.1136/jitc-2020-001895 PMC7813395

[15] Dmitrieva-Posocco, O. et al. Cell-Type-Specific Responses to Interleukin-1 Control Microbial Invasion and Tumor-Elicited Inflammation in Colorectal Cancer. *Immunity* **50**, 166–180.e7 (2019). 30650375 10.1016/j.immuni.2018.11.015 PMC6490968

[16] Isambert, N. et al. Fluorouracil and bevacizumab plus anakinra for patients with metastatic colorectal cancer refractory to standard therapies (IRAFU): a single-arm phase 2 study. *Oncoimmunology* **7**, e1474319 (2018). 30228942 10.1080/2162402X.2018.1474319 PMC6140586

[17] Bruchard, M. et al. Chemotherapy-triggered cathepsin B release in myeloid-derived suppressor cells activates the Nlrp3 inflammasome and promotes tumor growth. *Nat. Med.* **19**, 57–64 (2013). 23202296 10.1038/nm.2999

[18] Hickish, T. et al. MABp1 as a novel antibody treatment for advanced colorectal cancer: a randomised, double-blind, placebo-controlled, phase 3 study. *Lancet Oncol.* **18**, 192–201 (2017). 28094194 10.1016/S1470-2045(17)30006-2

[19] Mourtzakis, M. et al. A practical and precise approach to quantification of body composition in cancer patients using computed tomography images acquired during routine care. *Appl. Physiol. Nutr. Metab.* **33**, 997–1006 (2008). 18923576 10.1139/H08-075

[20] Ridker, P. M. et al. Modulation of the interleukin-6 signalling pathway and incidence rates of atherosclerotic events and all-cause mortality: analyses from the Canakinumab Anti-Inflammatory Thrombosis Outcomes Study (CANTOS). *Eur. Heart J.* **39**, 3499–3507 (2018). 30165610 10.1093/eurheartj/ehy310

[21] Ridker, P. M., MacFadyen, J. G., Thuren, T. & Libby, P. Residual inflammatory risk associated with interleukin-18 and interleukin-6 after successful interleukin-1b inhibition with canakinumab: further rationale for the development of targeted anti-cytokine therapies for the treatment of atherothrombosis. *Eur Heart J.* **41**, 2153–2163 (2020). 31504417 10.1093/eurheartj/ehz542

[22] Li, J. et al. Effect of Fruquintinib vs Placebo on Overall Survival in Patients With Previously Treated Metastatic Colorectal Cancer: The FRESCO Randomized Clinical Trial. *JAMA* **319**, 2486–2496 (2018). 29946728 10.1001/jama.2018.7855 PMC6583690

[23] Limagne, E. et al. Trifluridine/Tipiracil plus Oxaliplatin Improves PD-1 Blockade in Colorectal Cancer by Inducing Immunogenic Cell Death and Depleting Macrophages. *Cancer Immunol. Res.* **7**, 1958–1969 (2019). 31611243 10.1158/2326-6066.CIR-19-0228

[24] Patel, M. R., Falchook, G. S., Hamada, K., Makris, L. & Bendell, J. C. A phase 2 trial of trifluridine/tipiracil plus nivolumab in patients with heavily pretreated microsatellite-stable metastatic colorectal cancer. *Cancer Med.* **10**, 1183–1190 (2021). 33544407 10.1002/cam4.3630 PMC7926002

[25] Pagès, F. et al. Effector Memory T Cells, Early Metastasis, and Survival in Colorectal Cancer. *N. Engl. J. Med.* **353**, 2654–2666 (2005). 16371631 10.1056/NEJMoa051424

[26] Reichling, C. et al. Artificial intelligence-guided tissue analysis combined with immune infiltrate assessment predicts stage III colon cancer outcomes in PETACC08 study. *Gut* **69**, 681–690 (2020). 31780575 10.1136/gutjnl-2019-319292 PMC7063404

[27] Nobin, H. et al. The prognostic value of programmed death-ligand 1 (PD-L1) expression in resected colorectal cancer without neoadjuvant therapy - differences between antibody clones and cell types. *BMC Cancer* **24**, 1051 (2024). 39187798 10.1186/s12885-024-12812-7 PMC11346183

[28] Fumet, J.-D. et al. Phase I/II study of trifluridine/tipiracil plus XB2001 versus trifluridine/tipiracil in metastatic colorectal cancer. *Future Oncol.* **0**, 1–9 (2024). 10.1080/14796694.2024.2415280 PMC13178176 39530624

[29] Godet, Y. et al. Analysis of Spontaneous Tumor-Specific CD4 T-cell Immunity in Lung Cancer Using Promiscuous HLA-DR Telomerase-Derived Epitopes: Potential Synergistic Effect with Chemotherapy Response. *Clin. Cancer Res.* **18**, 2943–2953 (2012). 22407833 10.1158/1078-0432.CCR-11-3185

[30] Laheurte, C. et al. Immunoprevalence and magnitude of HLA-DP4 versus HLA-DR-restricted spontaneous CD4+ Th1 responses against telomerase in cancer patients. *OncoImmunology* **5**, e1137416 (2016). 27467955 10.1080/2162402X.2015.1137416 PMC4910811

[31] Laheurte, C. et al. Distinct prognostic value of circulating anti-telomerase CD4+ Th1 immunity and exhausted PD-1+/TIM-3+ T cells in lung cancer. *Br. J. Cancer* **121**, 405–416 (2019). 31358938 10.1038/s41416-019-0531-5 PMC6738094

[32] Moodie, Z. et al. Response definition criteria for ELISPOT assays revisited. *Cancer Immunol. Immunother.* **59**, 1489–1501 (2010). 20549207 10.1007/s00262-010-0875-4 PMC2909425

[33] Hothorn, T. & Lausen, B. On the exact distribution of maximally selected rank statistics. *Comput. Stat. Data Anal.* **43**, 121–137 (2003).

