# Supplementary material for: Safety and efficacy of trifluridine/tipiracil +/− bevacizumab plus XB2001 (anti-IL-1α antibody): a single-center phase 1 trial
Source: Signal Transduct Target Ther. 2025 Jan 17;10:22. doi: 10.1038/s41392-024-02116-4 (PMC11739593; doi:10.1038/s41392-024-02116-4)
Supplement: Supplementary file 1 — Supplemental Material [file 41392_2024_2116_MOESM1_ESM.docx]

Supplementary Materials for

Safety and efficacy of trifluridine/tipiracil +/- bevacizumab plus XB2001 (anti-IL-1α antibody): a single-center phase 1 trial

Marion Thibaudin^1,2,3,4^, Nicolas Roussot^1,2,3,4,5^, Chloé Burlot^6^, Antonin Schmitt^6^, Julie Vincent^5^, Zoé Tharin^5^, Leila Bengrine^5^, Hélène Bellio^5^, Aurélie Bertaut^7^, Léa Hampe^1,2^, Susy Daumoine^1,2^, Emilie Rederstorff^8^, Morgane Peroz^1,2^, Titouan Huppe^1,2^, Valentin Derangère^1,2,4,5^, David Rageot^1,2,4^, John Simard^9^, Caroline Truntzer^1,2^, Jean David Fumet^1,2,3,4,5^, Francois Ghiringhelli^1,2,3,4,5^

Correspondence to: [fghiringhelli@cgfl.fr](mailto:fghiringhelli@cgfl.fr) or [mthibaudin@cgfl.fr](mailto:mthibaudin@cgfl.fr)

**This PDF file includes:**

Figures. S1 to S4

Tables S1 to S7

Figure. S1.

**
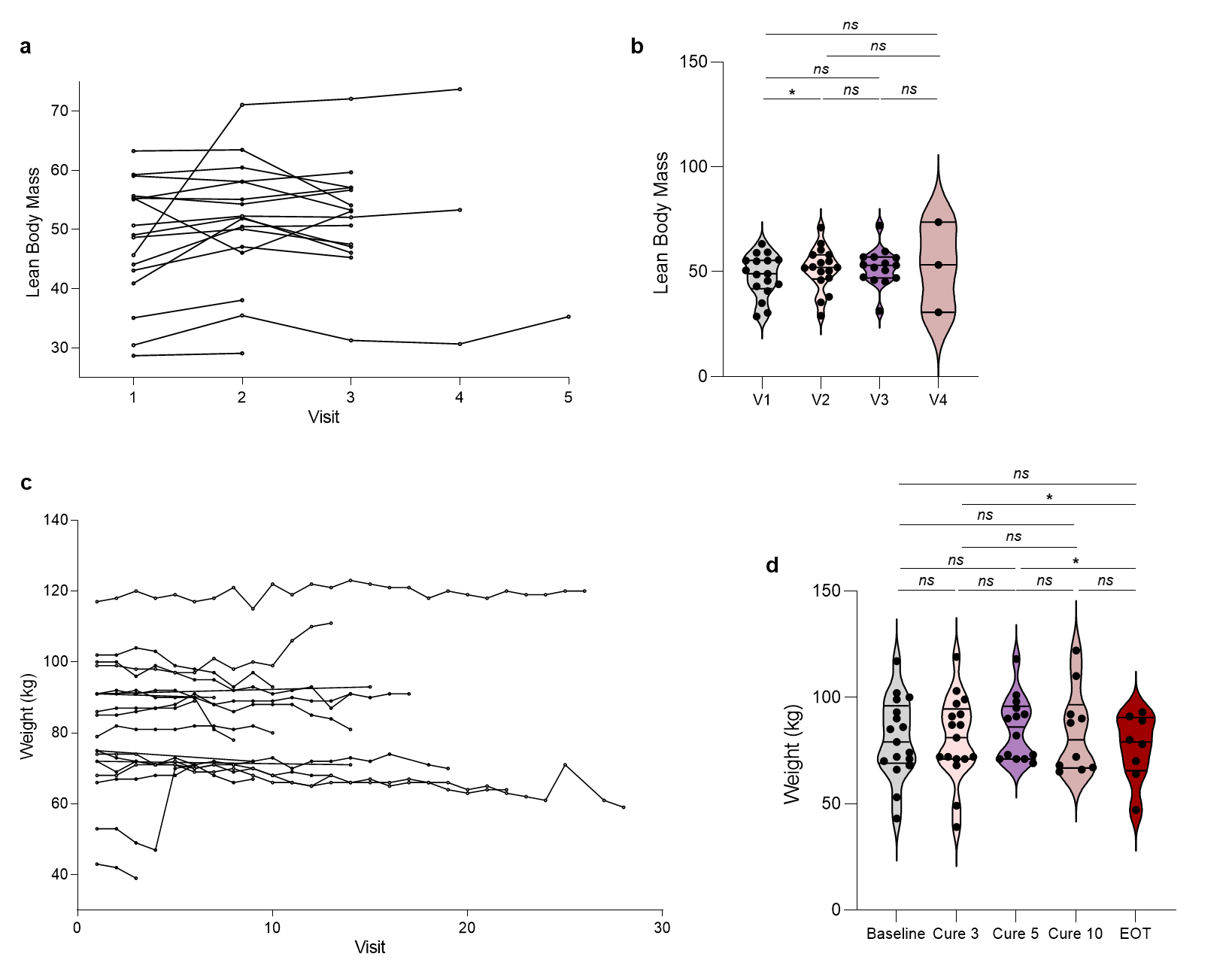
**

**Figure S1.**

**a,** Spider plot showing the evolution of lean body mass during treatment. Each line corresponds to one patient.

**b,** Violin plot showing the lean body mass at V1, V2, V3 and V4. n.s, not significant; *p < 0.05, comparison using Wilcoxon matched-paired test.

**c,** Spider plot showing changes in patients' weight during treatment. Each line corresponds to one patient.

**d,** Violin blot showing weight at baseline and at the beginning of Cure 3, Cure 5 and at the end of treatment (EOT). n.s, not significant; *p < 0.05, comparison using Wilcoxon matched-paired test.

Figure. S2.


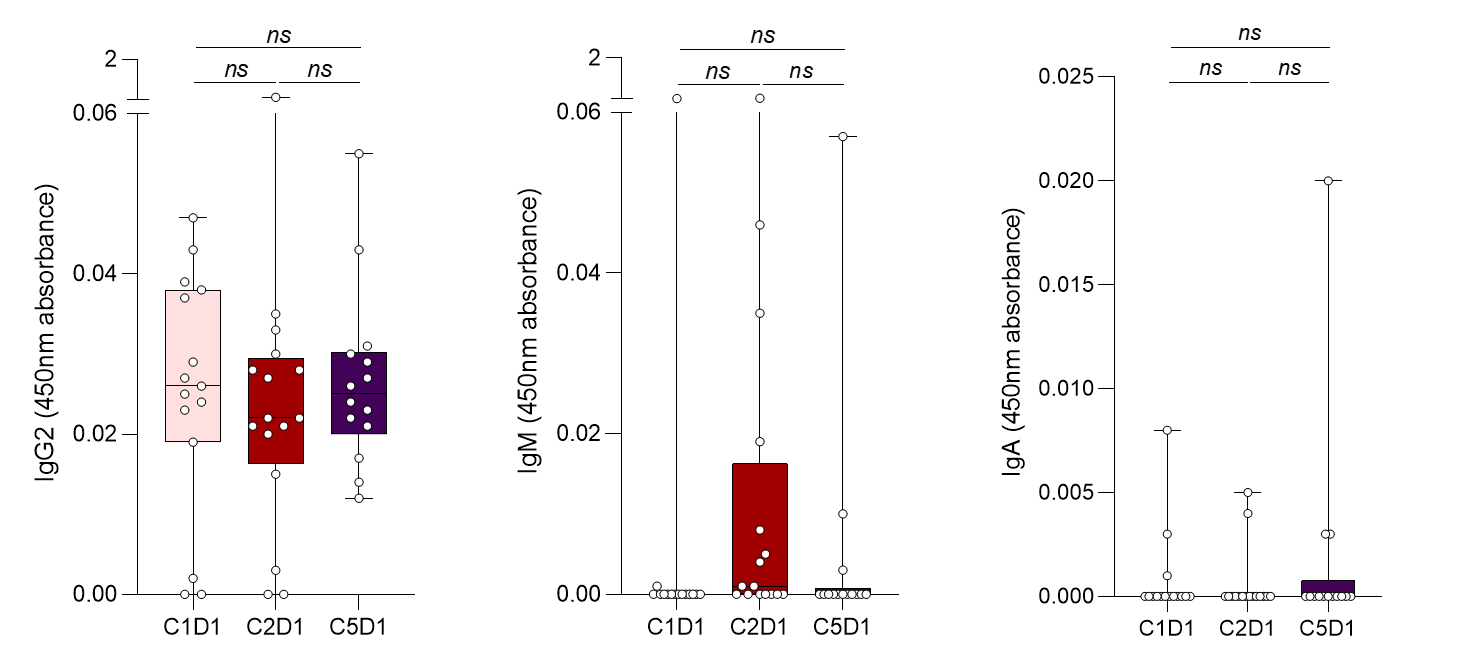


**Figure S2.** Measurement of IgG2 (**left**), IgM (**middle**) and IgA (**right**) anti-drug antibody concentrations in patient plasma at C1D1, C2D1 and C5D1.

Figure S3.


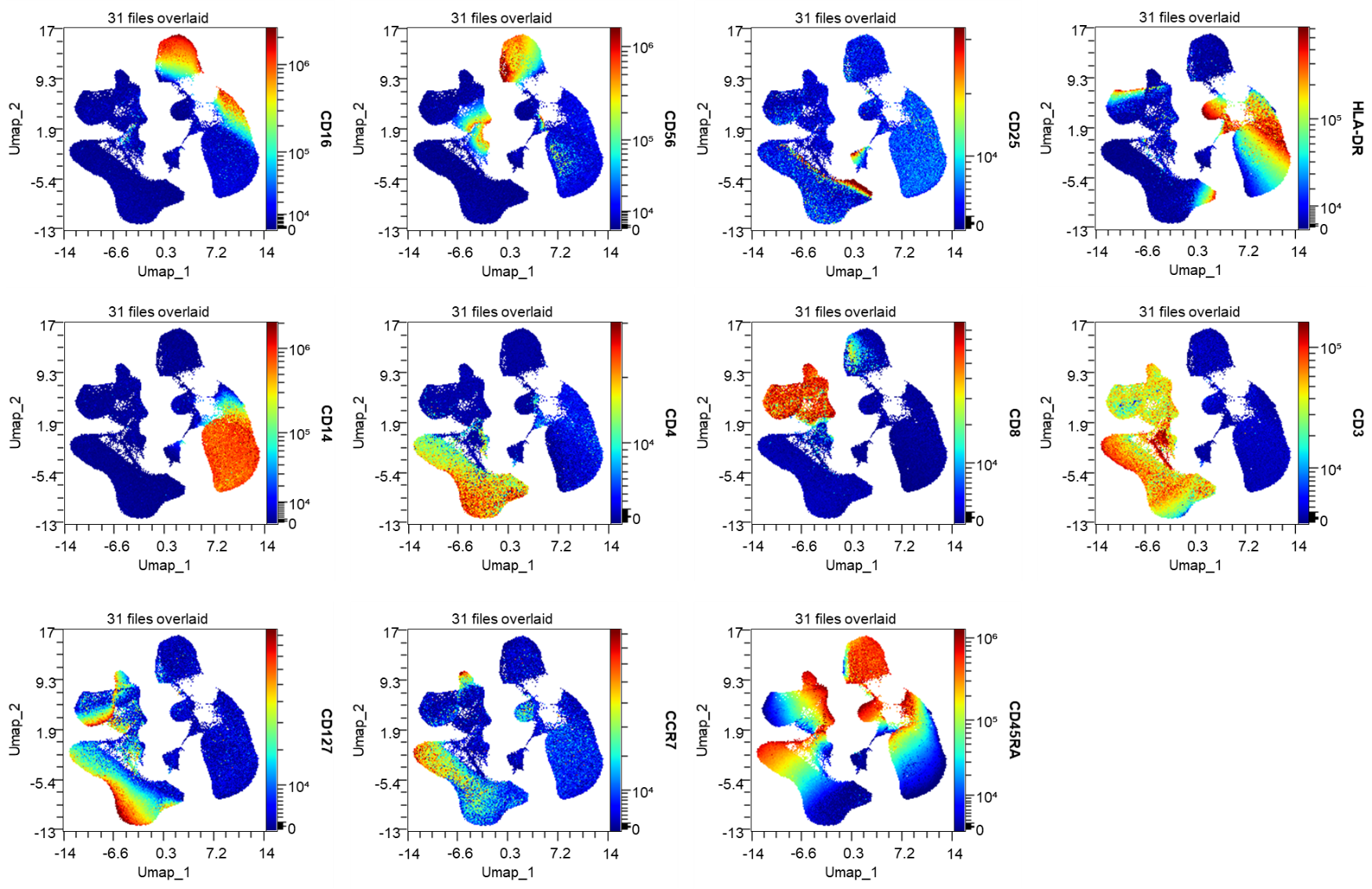


**Figure S3.** Immune cell marker expression is visualised on the UMAP map by color heat map.

Figure. S4.

**Figure S4.** Survival curves showing the probability of survival of colorectal cancer patients according to high or low *CD274* gene expression.

Table S1.

| **Table S1. Toxicity per dose level** | |  |  | |  |  |
| --- | --- | --- | --- | --- | --- | --- |
| ***System Organ Disorder*** | ****250 mg*** | ***500 mg*** | ***1000 mg*** | | ***1000 mg + bevacizumab*** |  |
|  | ***No. (%)*** | ***No. (%)*** | ***No. (%)*** | ***No. (%)*** | |  |
| **Gastrointestinal disorders** | | | | | |  |
| Diarrhoea | 1 (33.3%) | 2 (66.7%) | 1 (14.3%) | 2 (50%) | |  |
| Nausea | 0 | 2 (66.7%) | 3 (42.9%) | 3 (75%) | |  |
| Vomiting | 0 | 2 (66.7%) | 1 (14.3%) | 1 (25%) | |  |
| Abdominal pain | 0 | 1 (33.3%) | 0 | 2 (50%) | |  |
| Constipation | 0 | 2 (66.7%) | 1 (14.3%) | 0 | |  |
| **General disorders** | | | | | |  |
| Asthenia | 1 (33.3%) | 1 (33.3%) | 1 (14.3%) | 1 (25%) | |  |
| Decreasing appetite | 2 (66.7%) | 1 (33.3%) | 1 (14.3%) | 0 | |  |
| **Vascular disorders** |  |  |  |  | |  |
| Hypertension | 1 (33.3%) | 0 | 0 | 0 | |  |
| Hypotension | 1 (33.3%) | 0 | 0 | 0 | |  |
| **Immune system disorders** | | | | | |  |
| Infection | 0 | 0 | 2 (28.6%) | 0 | |  |
| **Skin and subcutaneous tissue disorders** | | | | | |  |
| Stomatitis | 0 | 0 | 0 | 2 (50%) | |  |
| Hand and foot syndrome | 1 (33.3%) | 0 | 0 | 0 | |  |
| **Blood and lymphatic system disorders** | | | | | |  |
| Anemia | 2 (66.7%) | 1 (33.3%) | 1 (14.3%) | 2 (50%) | |  |
| Neutropenia | 2 (66.7%) | 0 | 2 (28.6%) | 0 | |  |
| Thrombopenia | 1 (33.3%) | 1 (33.3%) | 2 (28.6%) | 2 (50%) | |  |
| **Liver function test disorders** | | | | | |  |
| Gamma GT increased | 2 (66.7%) | 0 | 0 | 0 | |  |
| Alanine aminotransferase increased | 1 (33.3%) | 0 | 0 | 0 | |  |
| PAL increased | 1 (33.3%) | 0 | 2 (28.6%) | 1 (25%) | |  |
| Aspartate aminotransferase increased | 1 (33.3%) | 0 | 0 | 0 | |  |
| Hyperbilirubinemia | 0 | 0 | 1 (14.3%) | 1 (25%) | |  |
| **Neuromuscular disorders** | | | | | |  |
| Myalgia | 2 (66.7%) | 0 | 0 | 0 | |  |
|  |  |  |  |  | |  |

**Table S1.** Toxicity per dose level

Table S2.

| **Table S2. Toxicity during the follow-up period** | | | |  |  | |
| --- | --- | --- | --- | --- | --- | --- |
| ***System Organ Disorder*** | ***Grade 0*** | ***Grade 1*** | ***Grade 2*** | ***Grade 3/4*** |  | |
|  | ***No. (%)*** | ***No. (%)*** | ***No. (%)*** | ***No. (%)*** |  |  |
| **Gastrointestinal disorders** | | | | |  |  |
| Diarrhoea | 10 (58.8%) | 3 (17.6%) | 3 (17.6%) | 1 (5.9%) |  |  |
| Nausea | 6 (35.3%) | 6 (35.3%) | 4 (23.5%) | 1 (5.9%) |  |  |
| Vomiting | 6 (35.3%) | 6 (35.3%) | 4 (23.5%) | 1 (5.9%) |  |  |
| Abdominal pain | 13 (76.5%) | 3 (17.6%) | 1 (5.9%) | 0 |  |  |
| Constipation | 16 (94.1%) | 1 (5.9%) | 0 | 0 |  |  |
| **General disorders** | | | | |  |  |
| Asthenia | 11 (64.7%) | 5 (29.4%) | 1 (5.9%) | 0 |  |  |
| Decreasing appetite | 14 (82.4%) | 3 (17.6%) | 0 | 0 |  |  |
| **Vascular disorders** |  |  |  |  |  |  |
| Hypertension | 0 | 0 | 0 | 0 |  |  |
| Hypotension | 16 (94.1%) | 1 (5.9%) | 0 | 0 |  |  |
| **Immune system disorders** | | | | |  |  |
| Infection | 16 (94.1%) | 1 (5.9%) | 0 | 0 |  |  |
| **Skin and subcutaneous tissue disorders** | | | | |  |  |
| Stomatitis | 16 (94.1%) | 1 (5.9%) | 0 | 0 |  |  |
| Hand and foot syndrome | 16 (94.1%) | 1 (5.9%) | 0 | 0 |  |  |
| **Blood and lymphatic system disorders** | | | | |  |  |
| Anemia | 8 (47.1%) | 4 (23.5%) | 4 (23.5%) | 1 (5.9%) |  |  |
| Neutropenia | 11 (64.7%) | 1 (5.9%) | 2 (11.8%) | 3 (17.6%) |  |  |
| Thrombopenia | 10 (58.8%) | 7 (41.2%) | 0 | 0 |  |  |
| **Liver function test disorders** | | | | |  |  |
| Gamma GT increased | 15 (88.2%) | 0 | 1 (5.9%) | 1 (5.9%) |  |  |
| Alanine aminotransferase increased | 15 (88.2%) | 0 | 1 (5.9%) | 1 (5.9%) |  |  |
| PAL increased | 12 (70.6%) | 4 (23.5%) | 1 (5.9%) | 0 |  |  |
| Aspartate aminotransferase increased | 15 (88.2%) | 0 | 1 (5.9%) | 1 (5.9%) |  |  |
| Hyperbilirubinemia | 14 (82.4%) | 2 (11.8%) | 1 (5.9%) | 0 |  |  |
| **Neuromuscular disorders** | | | | |  |  |
| Myalgia | 16 (94.1%) | 1 (5.9%) | 0 | 0 |  |  |
|  |  |  |  |  |  |  |

**Table S2.** Toxicity during the follow-up period

Table S3.

| **Table S3. Discontinuation of treatment** | | | | | |  |
| --- | --- | --- | --- | --- | --- | --- |
| ***Toxicity type*** | ****250 mg*** | ***500 mg*** | ***1000 mg*** | | ***1000 mg + bevacizumab*** |  |
|  | ***No. (%)*** | ***No. (%)*** | ***No. (%)*** | ***No. (%)*** | |  |
| **Hematological toxicity** | | | | | |  |
| Neutropenia | 1 (33.3%) | 0 | 1 (14.3%) | 0 | |  |
| Angiocholite | 1 (33.3%) | 0 | 0 | 0 | |  |
| Cholestase | 1 (33.3%) | 0 | 0 | 0 | |  |
| Hepatological toxicity | 1 (33.3%) | 0 | 0 | 0 | |  |
| **Non-hematological toxicity** | | | | | |  |
| Asthenia | 1 (33.3%) | 0 | 0 | 0 | |  |
| Infection | 1 (33.3%) | 0 | 0 | 0 | |  |
| Diarrhea | 0 | 1 (33.3%) | 1 (14.3%) | 0 | |  |
| Sepsis | 0 | 1 (33.3%) | 1 (14.3%) | 0 | |  |
| Pain | 0 | 0 | 0 | 0 | |  |
| Crusty rhinitis | 0 | 0 | 1 (14.3%) | 0 | |  |
|  |  |  |  |  | |  |

**Table S3.** Discontinuation of treatment

Table S4.

| **Table S4. Univariate analysis of pharmacokinetics paramaters and outcome** | | | | |
| --- | --- | --- | --- | --- |
| **Characteristic** | **HR***^1^* | **95% CI***^1^* | **p-value** | |
| AUC_0-last_ (h.μg/mL) | 2.21 | 0.87, 5.61 | | 0.094 |
| Cl (mL/min) | 0.58 | 0.24, 1.44 | | 0.24 |
| Cmax (μg/mL) | 1.88 | 0.75, 4.74 | | 0.18 |
| T1/2 (jours) | 1.74 | 0.84, 3.58 | | 0.14 |
| Tmax (h) | 1.53 | 0.77, 3.05 | | 0.22 |
| Vd (L) | 1.85 | 0.81, 4.22 | | 0.14 |
| Cres 1st adm(μg/mL) | 2.11 | 0.87, 5.13 | | 0.10 |
| Cres 2nd adm (μg/mL) | 2.26 | 0.84, 6.07 | | 0.11 |

*^1^* HR = Hazard Ratio, CI = Confidence Interval

**Table S4.** Univariate analysis of pharmacokinetics parameters and outcome

Table S5.

| **Table S5. Univariate analysis of pharmacokinetics parameters and treatment response** | | | | |
| --- | --- | --- | --- | --- |
| **Characteristic** | **No Response**,  N = 12*^1^* | **Response**,  N = 4*^1^* | | **p-value***^2^* |
| AUC_0-last_ (h.μg/mL) | 61,857 (46,724, 70,482) | 63,240 (51,838, 64,827) | 0.7 | |
| Cl (mL/min) | 0.078 (0.067, 0.101) | 0.099 (0.091, 0.124) | 0.4 | |
| Cmax (μg/mL) | 237 (218, 248) | 238 (212, 242) | 0.8 | |
| T1/2 (jours) | 18 (11, 32) | 20 (15, 23) | 0.8 | |
| Tmax (h) | 2 (2, 8) | 2 (1, 2) | 0.2 | |
| Vd (L) | 3.69 (2.36, 4.15) | 4.02 (3.30, 4.22) | 0.7 | |
| Cres 1st adm(μg/mL) | 138 (85, 178) | 151 (115, 158) | 0.9 | |
| Cres 2nd adm (μg/mL) | 189 (147, 208) | 180 (143, 185) | 0.4 | |

*^1^* Median (IQR)*^2^* Wilcoxon rank sum exact test; Wilcoxon rank sum test

**Table S5.** Univariate analysis of pharmacokinetics parameters and treatment response

Table S6.

| **Table S6. Univariate and multivariate analysis of immune biomarkers tested** | | | | | | | |
| --- | --- | --- | --- | --- | --- | --- | --- |
|  | **Univariate** | | | | **Multivariate** | | |
| **Variable** | **N** | **HR**^1^ | **95% CI**^1^ | **p-value** | **HR**^1^ | **95% CI**^1^ | **p-value** |
| **CD8** | 14 |  |  |  |  |  |  |
| 0-2 |  | — | — |  | — | — |  |
| 3 |  | 0.16 | 0.03, 0.69 | **0.042** | 0.16 | 0.01, 3.17 | 0.2 |
| **PDL1** | 14 |  |  |  |  |  |  |
| 0 |  | — | — |  | — | — |  |
| 1-3 |  | 0.14 | 0.02, 1.08 | **0.018** | 0.21 | 0.02, 1.87 | 0.2 |
| **IL1** | 16 |  |  |  |  |  |  |
| 0 |  | — | — |  | — | — |  |
| 1-2 |  | 0.34 | 0.07, 1.67 | 0.07 | 0.44 | 0.05, 3.52 | 0.4 |
| **Num_CD3_pos_per_mm2** | 14 |  |  |  |  |  |  |
| Low |  | — | — |  | — | — |  |
| High |  | 0.29 | 0.06, 1.28 | 0.10 | 0.09 | 0.01, 0.76 | **0.027** |
| ^1^HR = Hazard Ratio, CI = Confidence Interval | | | | | | | |

Table S6. Univariate and multivariate analysis of immune biomarkers tested

Table S7.

| **Table S7. List of cytokines analyzed by Luminex Assay** | |
| --- | --- |
| **Cytokine** | **Assay Working range (pg/ml)** |
| CD40 Ligand | 1659 - 59,995 |
| EGF | 32.6 - 1102 |
| Eotaxin | 63.9 - 2299 |
| FGF basic | 34.0 - 1084 |
| Flt-3 Ligand | 81.7 - 2783 |
| G-CSF | 29.8 - 1008 |
| GM-CSF | 60.9 - 2069 |
| Granzyme B | 14.3 - 469 |
| Groα | 173 - 2264 |
| Groβ | 38.3 - 1247 |
| IFNα | 14.7 - 500 |
| IFNβ | 13.0 - 446 |
| IFNγ | 18.1 - 611 |
| IL-1α | 36.0 - 1199 |
| IL-1β | 12.2 - 432 |
| IL-1ra | 41.9 - 1386 |
| IL-2 | 12.9 - 433 |
| IL-3 | 61.0 - 2093 |
| IL-4 | 4.10 - 140 |
| IL-5 | 22.0 - 780 |
| IL-6 | 34.3 - 1161 |
| IL-7 | 14.1 - 484 |
| IL-8 | 5.31 - 184 |
| IL-10 | 102 - 2997 |
| IL-12 p70 | 72.7 - 2419 |
| IL-13 | 111 - 3847 |
| IL-15 | 7.86 - 274 |
| IL-17A | 29.0 - 979 |
| IL-17E | 58.3 - 1938 |
| IL-33 | 60.4 - 1926 |
| IP-10 | 9.52 - 334 |
| MCP-1 | 45.1 - 626 |
| MIP-1α | 42.9 - 571 |
| MIP-1β | 319 - 11,240 |
| MIP-3α | 9.08 - 307 |
| MIP-3β | 14.5 - 483 |
| PDGF-AA | 15.8 - 537 |
| PDGF-AB/BB | 24.6 - 867 |
| PD-L1 | 271 - 9672 |
| RANTES | 1198 - 36,884 |
| TGF-α | 31.8 - 1098 |
| TNFα | 34.8 - 1325 |
| TRAIL | 87.3 - 2972 |
| VEGF | 19.9 - 678 |

Table S7. List of cytokines analyzed by Luminex Assay
