# Supplementary material for: Safety and efficacy of trifluridine/tipiracil +/− bevacizumab plus XB2001 (anti-IL-1α antibody): a single-center phase 1 trial
Source: Signal Transduct Target Ther. 2025 Jan 17;10:22. doi: 10.1038/s41392-024-02116-4 (PMC11739593; doi:10.1038/s41392-024-02116-4)
Supplement: Supplementary file 2 — Protocol of the clinical trial TASKIN [file 41392_2024_2116_MOESM2_ESM.pdf]

EudraCT N°: 2022-001400-18

Multicentric randomized phase I / II study to evaluate efficacy of trifluridine/tipiracil +/- bevacizumab plus XB2001 (anti-IL-1α True Human antibody) versus trifluridine/tipiracil +/- bevacizumab plus placebo in metastatic colorectal cancer patients after failure of oxaliplatin, irinotecan, fluoropyrimidine

TASKIN

	Version N° - Date version
FULL PROTOCOL	1.1 from 30/11/2020
	2.0 from 20/04/2021
	3.0 from 29/06/2021
	4.0 from 20/12/2021
	4.1 from 25/03/2022
	4.2 from 29/04/2022
	5.0 from 21/07/2022
	5.1 from 10/10/2022
	6.0 from 03/05/2023

**COORDINATING INVESTIGATORS**	**Pr GHIRINGHELLI François**<br>Centre Georges François Leclerc<br>1 rue du Pr. Marion<br>21000 DIJON<br>Phone : 03.80.73.77.76<br>Fax : 03.80.73.77.14<br>Email : FGhiringhelli@cgfl.fr
	**Pr LEPAGE Come**<br>CHU François Mitterand<br>2 Boulevard Maréchal de Lattre de tassigny<br>21000 Dijon<br>Phone : 03.80.29.37.50<br>Fax : 03.80.29.37.22<br>Email : come.lepage@u-bourgogne.fr

**SPONSOR**	**Centre Georges François Leclerc**<br>1 rue du Pr. Marion<br>21000 DIJON - FRANCE<br><br>*Tel. 03.45.34.81.16 - Fax: +33.3.80.73.77.53*

CLINICAL STUDY AUTORISATION FOR PROTOCOL TASKIN – FG_2020/04

Multicentric randomized phase I / II study to evaluate efficacy of trifluridine/tipiracil +/- bevacizumab plus XB2001 (anti-IL-1α True Human antibody) versus trifluridine/tipiracil +/- bevacizumab plus placebo in metastatic colorectal cancer patients after failure of oxaliplatin, irinotecan, fluoropyrimidine

**COORDINATIONG INVESTIGATORS :**<br>Pr GHRINGHELLI François<br><br><br><br>Pr LEPAGE Come	Department of Medical Oncology<br>Center Georges Francois Leclerc<br>1 rue du professeur Marion<br>21000 Dijon, France<br>Phone. : 03.80.73.77.76 / Fax : 03.80.73.77.14<br>Email : FGhiringhelli@cgfl.fr<br><br>Department of Gastroenterology and hepatology<br>CHU François Mitterand<br>2 Boulevard Maréchal de Lattre de tassigny<br>21000 Dijon<br>Phone : 03.80.29.37.50/Fax : 03.80.29.37.22<br>Email : come.lepage@u-bourgogne.fr
**BIOLOGICAL STUDY COORDINATOR :**<br>Dr FUMET Jean-David	Department of Medical Oncology<br>Center Georges Francois Leclerc<br>1 rue du professeur Marion<br>21000 Dijon, France<br>Phone. : 03.80.73.75.00 poste 33.03<br>Email : JDFumet@cgfl.fr
**METHODOLOGIST :**<br>Dr BERTAUT Aurélie	Methodology, biostatistics and data management unit (UMBD)<br>Center Georges Francois Leclerc<br>1 rue du professeur Marion<br>21000 Dijon, France<br>Phone. : 03.80.73.77.84<br>Email : ABertaut@cgfl.fr
**PROJECT MANAGER :**<br>REDERSTORFF Emilie	Clinical research center – promotion unit<br>Center Georges Francois Leclerc<br>1 rue du professeur Marion<br>21000 Dijon, France<br>Phone. : 03.45.34.81.16 / Fax : 03.80.73.77.53<br>Email : ERederstorff@cgfl.fr
**PHARMACOVIGILANCE NOTIFICATION**	R&D UNICANCER Pharmacovigilance Unit<br>Phone. : 01.44.23.04.16<br>Fax : 01.44.23.55.77<br>Email : pv-rd@unicancer.fr

REGULATORY CONSIDERATION

**COMPETENT AUTHORITY**	**Agence National de Sécurité du Médicament (ANSM)**	Registration date: 23/09/2020<br><br>Initiale autorisation : 24/05/2022
		ANSM reference: MEDAECNAT-2022-05-0020 _ 2022-001400-18
**ETHICS COMMITTEE**	**CPP SUD-OUEST et OUTRE MER IV**	Date of favorable opinion : 05/05/2022
		CPP reference : 22.00525.000075

Substantial modifications	
**N°**	**1**
**Date of ethics committee approval**	06/05/2022
**Date of competent authority (ANSM) authorization**	23/08/2022
**Protocole version**	5.0 from 21/07/2022
**Main modifications**	• Add mandatory sampling for patients from phase I (same sampling applicable for patients from phase II)<br>• Deletion of inclusion criteria n°14 and update of non-inclusion criteria n°13<br>• Corrections of inconsistencies in the protocol<br>• Implementation of the modifications requested by the CPP (for ANSM only)

Substantial modifications	
**N°**	**2**
**Date of ethics committee approval**	30/11/2022
**Date of competent authority (ANSM) authorization**	17/11/2022
**Protocole version**	5.1 from 10/10/2022
**Main modifications**	Clarification of the procedure for level 1 and level 2 following modifications performed in amendement 1 – patients from level 1 will be includ in level 2 after DLT period (28 days) if no DLT is observed.

Substantial modifications	
**N°**	**3**
**Date of ethics committee approval**	18/07/2023
**Date of competent authority (ANSM) authorization**	02/08/2023
**Protocole version**	6.0 from 03/05/2023
**Main modifications**	- Update of the standard of care with the abaility to add bevacizumab to trifluridine/tipiracil according to local practice of each investigator sites<br>- Addition of a level 5 in the phase I to evaluate the safety of the association of the trifluridine/tipiracil + bevacizumab and the dose of XB2001 determined at the end of the phase I. If level 5 results is in accordance with safety and tolerance, bevacizumab

	could be add in the phase II according to investigator discretion.<br>- Update of the Flowchart<br>- Clarification of the delay of 3 days between inclusion/randomization and first cycle C1D1<br>- Modification of the rythm of inclusion of the phase I with the possibility to include patients 3 by 3 for levels 4 and 5<br>- Clarification of IDMC meeting. Only one mandatory IDMC required after the inclusion of 10 patients in each ARM of the phase II. A second IDMC may be held after the inclusion of 26 patients in each ARM of the phase II and will be left to the choice of IDMC members to carry it out or not<br>- Update of the Investigator list (addition of sites from Angers, Carcassonne, Clermont-Ferrand and Reims for the phase II)<br>- Update of the end of study timing. End of study would be after 6 months of follow-up from the last treatment dose administration of the last patient included in the study<br>- Update of PK/PD study : EDTA tube replaces heparin tube

TABLE OF CONTENTS

1. INTRODUCTION AND RATIONALE OF THE STUDY 17

1.1 Location of the work in the context of the current knowledge 17

1.2 Originality and innovative aspects of this project 20

1.3 Patient benefits and foreseeable risk(s) Expected results 20

1.3.1. Potential benefit 20

1.3.2. Potential risk 20

1.4 Study population 20

2. STUDY OBJECTIVES 20

2.1 Main objectives 20

2.1.1. Main objective of phase I portion 21

2.1.2. Main objective of phase II portion 21

2.2 Secondary objectives 21

2.2.1. Secondary objective of phase I 21

2.2.2. Secondary objective of phase II 21

2.3 Exploratory objectives of phase I and II 21

3. EVALUATION CRITERIA 22

3.1 Primary end-points 22

3.1.1 Primary end-points of phase I portion 22

3.1.2 Primary end-points of phase II 22

3.2 Secondary endpoints 22

3.2.1 Secondary endpoints of phase I 22

3.2.2 Secondary endpoints of phase II 22

3.3 Exploratory endpoint of phase I and phase II 23

4. STUDY DESIGN 23

4.1 Study methodology 23

4.1.1. Phase I design (Dose escalation phase) 23

4.2 Progression of the study 25

4.3 Inclusion and registration procedures 26

4.4 Study early terminations/stopping rules and patient premature discontinuation 26

5. PATIENT SELECTION 28

5.1 Inclusion criteria 28

5.2 Non inclusion criteria 29

6. STUDY TREATMENTS 30

6.1 Investigational product: XB2001 31

6.1.1 Active ingredient, pharmacologic class, structure 31

6.1.2 Drug product (XB2001 100mg/mL) description 32

6.1.3 Storage 32

6.1.4 Stability 32

6.1.5 Dosing and methods of administration 32

6.1.6 Justification for 1000 mg dose selection 32

6.2 Non-investigational product: trifluridine/tipiracil - Bevacizumab 35
6.3 Dose adaptation........ 36
6.3.1 General guidance 36
6.3.2 XB2001 dose adaptation 37
6.3.3 Trifluridine/tipiracil +/- bevacizumab dose adaptation 38
6.4 Assessments of trifluridine/tipiracil compliance 40
6.5 Shipment, storage, accountability and destruction........ 40
6.5.1 XB2001 / Placebo........ 40
6.5.2 Trifluridine/tipiracil +/- bevacizumab 41
6.6 Restriction during the study 41
6.6.1 Birth control measures........ 41
6.6.2 Blood donation........ 42
6.7 Concomitant treatments 42
6.7.1 Authorised concomitant treatments 42
6.7.2 Prohibited concomitant treatments 42
6.7.3 Concomitant treatments use with caution 42

7. EVALUATION OF TREATMENT EFFICACY AND SAFETY........43

7.1 Efficacy evaluation........ 43
7.2 Safety evaluation 43
7.2.1 Physical examination 44
7.2.2 Electrocardiograms........ 44
7.2.3 Vital signs and weight 44
7.2.4 Clinical laboratory tests 44

8. DESCRIPTION OF VISITS AND INVESTIGATIONS........44

8.1 Baseline/screening assessment for phase I or phase II of the study 45
8.1.1 Eligibility screening for baseline visit phase I or phase II 45
8.1.2 Demographics, medical and disease history for baseline visit phase I or phase II........ 45
8.1.3 Clinical examination for baseline visit phase I or phase II........ 45
8.1.4 Biologicals tests for baseline visit phase I or phase II 45
8.1.5 Paraclinical examinations for baseline visit phase I or phase II 46
8.2 Visits and assessment during the treatment period of phase I 46
8.2.1 Clinical examinations and assessments during the treatment period of phase I 46
8.2.2 Biological tests during the treatment period of phase I 46
8.2.3 Paraclinical examinations during the treatment period of phase I........ 47
8.2.4 Pharmacokinetics and pharmacodymacis sampling for phase I 47
8.3 Visits and assessment during the treatment period of phase II 47
8.3.1 Clinical examinations and assessments during the treatment period of phase II 47
8.3.2 Biological tests during the treatment period of phase II 47
8.3.3 Paraclinical examinations during the treatment period of phase II........ 48
8.4 End-of-treatment assessments for phase I and phase II........ 48
8.4.1 Clinical examination for End-of-treatment assessments for phase I and phase II........ 48
8.4.2 Biological tests for End-of-treatment assessments for phase I and phase II 48

8.4.3 Paraclinical examinations examination for End-of-treatment assessments for phase I and phase II 49
8.5 Post treatment follow-up visits for phase I and phase II 49
8.6 Treatment after study interruption for phase I and phase II 49
8.7 Emergency unblinding for phase II 49
8.8 Ancillary studies blood and tumor sampling for phase I and phase II 49

9. SAFETY EVALUATION 51

9.1 Adverse event 52
9.1.1 General definition 52
9.1.2 Evaluating Adverse Events 52
9.1.3 Adverse Event Reporting 53
9.1.4 Identified and potential risks of XB2001 53
9.1.5 Identified and potential risks of trifluridine/tipiracil 54
9.1.5 Identified and potential risks of bevacizumab 55
9.2 Serious adverse events 57
9.2.1 General definition 57
9.2.2 Other events requiring reporting 58
9.2.3 Maternal exposure 59
9.2.4 Paternal exposure 59
9.2.5 Suspected unexpected serious adverse reaction (SUSAR) 60
9.2.6 Measures to be taken in case of a serious adverse event 60

10. BLOOD AND TUMOR SAMPLES ANALYSIS 61

10.1 Peripheral immunomonitoring (blood samples – mandatory for patients from phase I and phase II) 61
10.2 Pharmacokinetics analysis (blood sample and handling – mandatory for patients from phase I only) 62
10.3 Dosing of trifluridine/tipiracil (mandatory for patients from phase II only) 62
10.4 Immunohistology procedure (tumor samples – initial mandatory and second optional for patients from phase I and phase II) 62
10.5 Methodology for statistical analysis of ancillary studies: 62

11. STATISTICAL ANALYSIS PLAN 63

11.1 Required number of patients 63
11.2 Definition of study population analysis 63
11.3 Statistical analysis of endpoints 64
11.4 Managing the missing and non-valid data 64
11.5 Interim analysis 64
11.5.1 Safety/Tolerance interim analysis 64
11.5.2 Efficacy interim analysis 64
11.6 Method of randomization 65
11.7 Conversion into Phase III trial 65

12. OVERSIGHT COMMITTEES 65

12.1 Independant data monitoring committee 65
12.2 Steering Committee 65

13. QUALITY ASSURANCE 66

13.1 Data collection 66
13.2 Study monitoring 66
13.3 Audits and Inspection 67
14. ETHICAL AND REGULATORY CONSIDERATIONS 67
14.1 General requirements 67
14.2 Clinical Study Authorisation 67
14.3 Patient identification 67
14.4 Patient information and consent 67
14.5 Changes to the study protocol 68
14.6 Sponsor responsabilities 68
14.7 Insurance compensation 69
14.8 Investigator responsabilities 69
14.9 Human biological samples collections 69
14.9.1 Strorage and use of disease assessment samples (blood, biopsy, tumour specimen) 69
14.9.2 Collecting additional biological samples for research purpose 70
15. DATA PROCESSING AND CONSERVATION OF DOCUMENTS AND DATA OF THE RESEARCH 70
15.1 Data processing 70
15.1.1 Under the responsability of the sponsor 70
15.1.2 In the investigational site, when computerised medical record are used 70
15.2 Retention of documents by investigator sites 71
16. DATA OWNERSHIP AND CONFIDENTIALITY 71
17. PUBLICATION RULES 71
18. REFERENCES 72

General study design

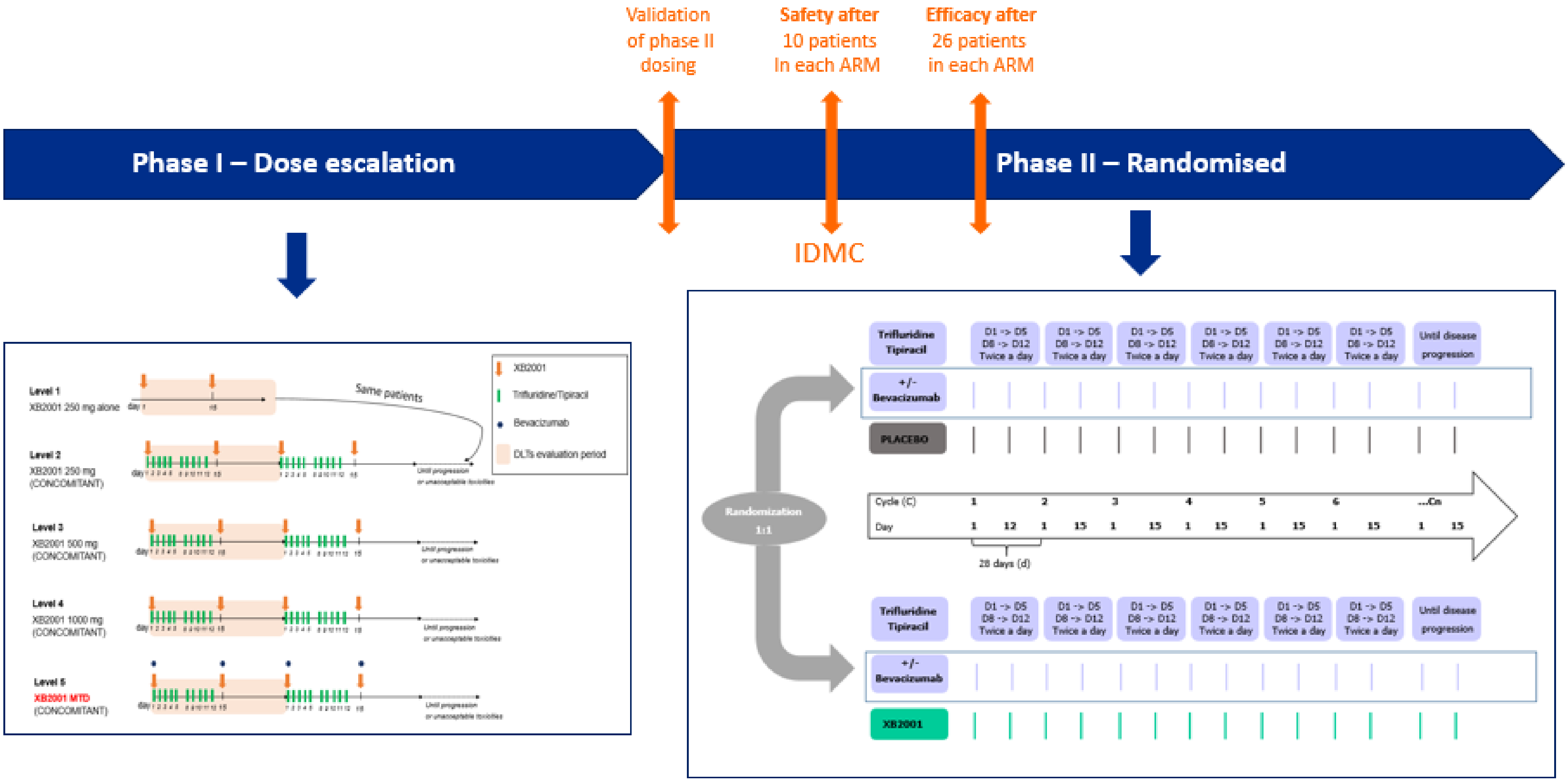

Table 1: Study flow chart for phase I study.

A general +/-3 days window is allowed for assessments or visit performed every 4 weeks and intermédiat administration of XB2001; 1 Cycle = 28 days.
A +/-7 days window is allowed for tumor evaluations. **Exception for the visite of day1 from Cycle 2** where no window is allowed in order to respect the timing of the PK/PD sampling mandatory at J29 following C1D1.

VISITS during phase 1 portion	BASELINE		FOLLOW-UP DURING TREATMENT															FOLLOW-UP AFTER TREATMENT
	≤ 21 days	≤ 14 days	C0 only for level 1		C1		C2		C3		C4		C5		C6, C7, C8, C^n…		End of treatment	Every 12 weeks up to 2 years
			D1	D15	D1	D15	D1	D15	D1	D15	D1	D15	D1	D15	D1	D15		
			V-1	*V-2*	*V1*	*V2*	*V3*	*V4*	*V5*	*V6*	*V7*	*V8*	*V9*	*V10*	*V^n*	*V^n*	*EOT*	*FU*
Signed informed consent form	X																	
eCRF registration	X																	
**PATIENT HISTORY**																		
Review of inclusion/non inclusion criteria	X																	
Demographic data	X																	
Cancer history and characteristics / prior therapies	X																	
Other relevant medical history	X																	
Collection of concomittant treatment		X[1]	X	X	X		X		X		X		X		X		X	
Collection of post-study new anti-neoplasic treatments																		X
Disease and survival status																		X
**SAFETY ASSESSMENTS**																		
**CLINICAL EXAMINATION**																		
Physical Examination		X	X		X		X		X		X		X		X		X	
Height		X																
Weight, PS (ECOG)		X	X		X		X		X		X		X		X		X	
Vital signs (BP, pulse, body temperature and respiratory rate)		X	X[13]	X[13]	X[13]	X[13]	X[13]	X[13]	X		X		X		X		X	
Adverse Event [2]	X	X	X	X	X	X	X		X		X		X		X		X	X
**PARACLINICAL EXAMINATION**																		
ECG [3]	X		*As clinically indicated*														X	
QLQC30 + QLQCR29	X						X				X				X[11]		X	
Patient diary / Compliance					X		X		X		X		X		X		X	
**BIOLOGICAL TESTS[4]**																		
Haematological and clinical chemistry		X	X	X	X	X	X		X		X		X		X		X	
ACE		X	X		X		X		X		X		X		X		X	
Urinalysis		X	X		X		X		X		X		X		X		X	
Pregnancy test [5] (urine hCG or Serum βhCG)		X	X		X		X		X		X		X		X		X	
Hepatitis and HIV serologies	X		*As clinically indicated*															
ACTIVITY ASSESSMENT																		
Radiological assessment by RECIST [7]	X				*Every 8 weeks until 12 months then every 12 weeks[9]*													X[8]

TREATMENT ADMINISTRATION(6)	BASELINE	C0 only for level 1		C1		C2		C3		C4		C5		C^n		End of treatment	Follow-up
XB2001		D1	D15	D1	D15	D1	D15	D1	D15	D1	D15	D1	D15	D1	D15		
Trifluridine/tipiracil +/- bevacizumab				D1 to D5	D8 to D12	D1 to D5	D8 to D12	D1 to D5	D8 to D12	D1 to D5	D8 to D12	D1 to D5	D8 to D12	D1 to D5	D8 to D12		

VISITS	BASELINE		FOLLOW-UP DURING TREATMENT																		FOLLOW-UP
	≤ 21 days	≤ 14 days	C0 only for level 1		C1		C2		C3		C4		C5		C^n		End of treatment	FU
			V-1	V-2	V1	V2	V3	V4	V5	V6	V7	V8	V9	V10	V^n	V^n	EOT	
**BIOLOGICAL SAMPLES MANDATORY**																		
Blood samples (1 EDTA of 6 mL) for PK and PD analysis			X(12)		X(12)		X(12)											
Blood samples for ancillary studies (5 EDTA of 10mL + 1 heparin 6mL (or 4mL) + 1 dry tube of 4 mL)					X(10)				X				X				X	
Tumor samples dated less than 2 years	X																	
**BIOLOGICAL SAMPLES OPTIONAL**																		
Biopsy of a primary or secondary sites									X									

(1) Data concerning all treatments received within 2 weeks of randomization. Check the current administration of prohibited medications and start considering substituting them.

(2) Ongoing toxicities or adverse event must be monitored until resolution or return to baseline level.
Information must be collected concerning adverse events occuring during the 30 days after the last administration of trifluridine/tipiracil +XB2001/placebo

(3) Baseline ECG should be performed in a triplicate measurement taken approximately 2-5mn apart only if clinical abnormality detected on the first ECG. During the study ECG is performed as clinically indicated at the discretion of the investigator.

(4) Biological test to be performed within 14 days prior to inclusion and within 72 hours before each cycle of trifluridine/tipiracil are described in the table below:

Test category	Test name
Haematology	Absolute Neutrophil count (ANC), absolute lymphocyte count, haemoglobin, platelet count
Clinical chemistry	Albumin, alkaline phosphatase, ASAT, ALAT, calcium, chloride, creatinine clearance, GGT, glucose, LDH, magnesium, potassium, sodium, total bilirubin, total protein.
Tumor markers	ACE
Urinalysis	Bilirubin, blood, colour and appearance, glucose, ketones, pH, **protein**, specific gravity.

(5) Pregnancy test: only for female pre-menopausal patients: urine within 72h or serum pregnancy test within 14 days prior to enrolment and at each 28-day cycle.

(6) Study treatment administration:
trifluridine/tipiracil [bid D1-D5; D8-D12; 1 cycle every 28 days] + XB2001 by intravenous infusion (250mg, 500mg or 1000mg according to the level tested for phase I) every 2 weeks (D1 and D15 of each cycle)

(7) CT scan (or RMI if CT scan is not indicated, but same method of evaluation should be maintained during all the study – a CT scan is recommended for the study) of the chest, abdomen and pelvis should be performed for RECIST assessment. Imaging disease assessment should be obtained every 8 weeks (+/-7 days), or sooner if clinically indicated, during the first 12 months of treatment phase, and every 12 weeks (+/-7 days) thereafter.

(8) Tumor assessment has to be continued during the post-treatment period if withdrawal was not related to disease progression and should be continued and documented every 8 weeks (or every 12 weeks after the first 12 months of treatment phase) until disease progression or initiation of an antineoplastic treatment.

(9) Radiological assessments should not be repeated if they were obtained less than 6 weeks from withdrawal therapy

(10) Blood samples should be drawn before the first dose of treatment is taken and at the latest 14 days before the first treatment administration.

(11) QLQC-30 and QLQCR29 will be performed every 2 cycles (Cycle 12, cycle14, cycle 16…)

(12) PK XB2001, 7 timepoints: day 1 before infusion / day 1 ≈ 30 minutes post infusion / day 2 / day 5 / day 8 / day 15 / day 29, (1 EDTA tube/time point)
PD, 3 timepoints: Day1 / Day 15 and Day 29
Patient from level 1 will performed PK, PD analysis only once.

(13) Blood pressure will be repeated during level 1, 2, 3 and 4 for all administration of XB2001 during the first two months, with measurement performed prior infusion, 30-minutes after the start of the infusion at the end of the infusion and 30 minutes after the end of the infusion

Table 2: Study flow chart for phase II study.

A general +/-3 days window is allowed for assessments or visit performed every 4 weeks and intermédiat administration of XB2001; 1 Cycle = 28 days. **Except for C1D12 that should be performed without any visit window for PK sampling**.

A +/-7 days window is allowed for tumor evaluations.

VISITS	BASELINE		FOLLOW-UP DURING TREATMENT															FOLLOW-UP AFTER TREATMENT
	≤ 21 days	≤ 14 days	C1		C2		C3		C4		C5		C6		C7, C8, C9, C^n…		End of treatment	Every 12 weeks up to 2 years
			D1	D12	D1	D15	D1	D15	D1	D15	D1	D15	D1	D15	D1	D15		
			V1	*V2*	*V3*	*V4*	*V5*	*V6*	*V7*	*V8*	*V9*	*V10*	*V11*	*V12*	*V^n*	*V^n*	*EOT*	*FU*
Signed informed consent form	X																	
eCRF registration / randomisation	X																	
**PATIENT HISTORY**																		
Review of inclusion/non inclusion criteria	X																	
Demographic data	X																	
Cancer history and characteristics / prior therapies	X																	
Other relevant medical history	X																	
Collection of concomittant treatment		X[1]	X		X		X		X		X		X		X		X	
Collection of post-study new anti-neoplasic treatments																		X
Disease and survival status																		X
**SAFETY ASSESSMENTS**																		
**CLINICAL EXAMINATION**																		
Physical Examination		X	X		X		X		X		X		X		X		X	
Height		X																
Weight, PS (ECOG)		X	X		X		X		X		X		X		X		X	
Vital signs (BP, pulse, body temperature and respiratory rate)		X	X		X		X		X		X		X		X		X	
Adverse Event [2]	X	X	X		X		X		X		X		X		X		X	X
**PARACLINICAL EXAMINATION**																		
ECG [3]	X		*As clinically indicated*														X	
QLQC30 + QLQCR29	X				X				X				X		X[11]		X	
Patient diary and compliance			X		X		X		X		X		X		X			
**BIOLOGICAL TESTS[4]**																		
Haematological		X	X	X	X		X		X		X		X		X		X	
Clinical chemistry		X	X	X	X		X		X		X		X		X		X	
ACE		X	X		X		X		X		X		X		X		X	
Urinalysis		X	X		X		X		X		X		X		X		X	
Pregnancy test [5] (urine hCG or Serum βhCG)		X	X		X		X		X		X		X		X		X	
Hepatitis and HIV serologies	X		*As clinically indicated*															
ACTIVITY ASSESSMENT																		
Radiological assessment by RECIST [7]	X		*Every 8 weeks until 12 months then every 12 weeks[9]*														X[9]	X[8]

TREATMENT ADMINISTRATION[6]	BASELINE		C1		C2		C3		C4		C5		C6		C^n		End of treatment	Follow-up
XB2001/placebo			D1	**D12[12]**	D1	D15	D1	D15	D1	D15	D1	D15	D1	D15	D1	D15		
Trifluridine/tipiracil +/- bevacizumab			D1 to D5	D8 to D12	D1 to D5	D8 to D12	D1 to D5	D8 to D12	D1 to D5	D8 to D12	D1 to D5	D8 to D12	D1 to D5	D8 to D12	D1 to D5	D8 to D12		

VISITS	BASELINE		FOLLOW-UP DURING TREATMENT															FOLLOW-UP
	≤ 21 days	≤ 14 days	C1		C2		C3		C4		C5		C6		C^n		End of treatment	*FU*
			V1	*V2*	*V3*	*V4*	*V5*	*V6*	*V7*	*V8*	*V9*	*V10*	*V11*	*V12*	*V^n*	*V^n*	*EOT*	
**BIOLOGICAL SAMPLES MANDATORY**																		
Tumor samples dated less than 2 years	X																	
Blood samples (5 EDTA of 10 mL + 1 Heparin of 6 or 4 mL + 1 dry tube of 6 mL)			X[10]				X				X						X	
Blodd samples (1 Heparin of 6 or 4 mL)				**D12[13]**														
**BIOLOGICAL SAMPLES OPTIONAL**																		
Biopsy of primary or secondary site							X											

(1) Data concerning all treatments received within 2 weeks of randomization. Check the current administration of prohibited medications and start considering substituting them.

(2) Ongoing toxicities or adverse event must be monitored until resolution or return to baseline level.
Information must be collected concerning adverse events occuring during the 30 days after the last administration of trifluridine/tipiracil +XB2001/placebo

(3) Baseline ECG should be performed in a triplicate measurement taken approximately 2-5mn apart only if clinical abnormality detected on the first ECG. During the study ECG is performed as clinically indicated at the discretion of the investigator.

(4) Biological test to be performed within 14 days prior to inclusion and within 72 hours before each cycle of trifluridine/tipiracil are described in the table below :

Test category	Test name
Haematology	Absolute Neutrophil count (ANC), absolute lymphocyte count, haemoglobin, platelet count
Clinical chemistry	Albumin, alkaline phosphatase, ASAT, ALAT, calcium, chloride, creatinine clearance, GGT, glucose, LDH, magnesium, potassium, sodium, total bilirubin, total protein.
Tumor markers	ACE
Urinalysis	Bilirubin, blood, colour and appearance, glucose, ketones, pH, **protein**, specific gravity.

(5) Pregnancy test: only for female pre-menopausal patients: urine within 72h or serum pregnancy test within 14 days prior to enrolment and at each 28-day cycle.

(6) Study treatment administration:
trifluridine/tipiracil [bid D1-D5; D8-D12; 1 cycle every 28 days] + XB2001/placebo by intravenous infusion (250mg, 500mg or 1000mg according to phase I results) every 2 weeks (D1 and D15 of each cycle)

(7) CT scan (or RMI if CT scan is not indicated, but same method of evaluation should be maintened during all the study – a CT scan is recommended for the study) of the chest, abdomen and pelvis should be performed for RECIST assessment. Imaging disease assessment should be obtained every 8 weeks (+/-7 days), or sooner if clinically indicated, during the first 12 months of treatment phase, and every 12 weeks (+/-7 days) thereafter.

(8) Tumor assessment has to be continued during the post-treatment period if withdrawal was not related to disease progression and should be continued and documented every 8 weeks (or every 12 weeks after the first 12 months of treatment phase) until disease progression or initiation of an antineoplastic treatment.

(9) Radiological assessments should not be repeated if they were obtained less than 6 weeks from withdrawal therapy

(10) Blood samples should be drawn before the first dose of treatment is taken and at the latest 14 days before the first treatement administration.

(11) QLQC-30 and QLQCR29 will be performed every 2 cycles (Cycle 8, 10, 12, 14, cycle 16…)

(12) At cycle 1 administration of XB2001 should be performed at Day 12 in order to be done at the same time of blood samples for trifluridine/tipiracil dosing. Other 2nd injection of XB2001 performed during cycle should be done at Day 15 +/-3 days.

(13) The date and the time of the administration of trifluridine at day 1 and at D12 will be collected

1. INTRODUCTION AND RATIONALE OF THE STUDY

1.1 Location of the work in the context of the current knowledge

Colorectal cancer (CRC) is the second most commonly diagnosed cancer in Europe and a leading cause of death both in Europe and worldwide[1]. Chemotherapy remains the cornerstone treatment for metastatic colorectal cancer (mCRC). When all metastatic sites cannot be surgically removed, treatment remains palliative and requires different chemotherapeutic protocols. However, the use of palliative systemic chemotherapy dramatically enhances response rates, progression-free survival (PFS) and overall survival (OS). Treatment is currently based on the use of three cytotoxic chemotherapy drugs – fluoropyrimidine, oxaliplatin and irinotecan – combined with targeted therapies (anti-EGFR (panitumumab or cetuximab) or anti-VEGF (bevacizumab or aflibercept) monoclonal antibodies)[2]. However, if these treatments fail, few therapeutic options remain. Regorafenib and trifluridine/tipiracil have recently been added to the therapeutic arsenal as third line therapies[3].

For the regorafenib phase III study (CORRECT), median progression free survival was 2.8 months (95% CI 1.4–3.7) versus 1.8 months (95% CI 1.3–1.7) for placebo, (hazard ratio 0.49; p < 0 · 0001). The median overall survival was 6.4 months (95% CI 3.6–11.8) versus 5.0 months (95% CI 2.8–10.4) for placebo (hazard ratio 0.77; p = 0.0052)[3]. Recently, trifluridine/tipiracil was also tested in this context. Trifluridine/tipiracil is an oral combination drug of trifluridine and tipiracil, at a molar ratio of 1:0.5. Trifluridine is a fluorinated thymidine analog. Like 5-FU, it can inhibit thymidylate synthase, but its main effect relies on its incorporation into DNA, resulting in DNA dysfunction[4]. Tipiracil enhances the bioavailability of trifluridine by inhibiting its enzymatic degradation by thymidine phosphorylase, leading to more durable and sustained responses. Trifluridine/tipiracil was recently compared to placebo in the same clinical setting than regorafenib. In this study, a median progression-free survival of 2.0 months (95% CI, 1.9 to 2.1) in the trifluridine/tipiracil group and 1.7 months (95% CI, 1.7 to 1.8) in the placebo group (hazard ratio 0.48; p < 0.001), was observed. The median overall survival improved from 5.3 months (95% CI, 4.6–6) to 7.1 months (95% CI, 6.5–7.8) (hazard ratio 0.68; p < 0.001)[5]. Due to its safety and efficacy profile, trifluridine/tipiracil is largely used in France after failure of classical therapies (oxaliplatin, irinotecan, 5 fluoraouracil, bevacizumab, and anti EGFR).

Recently the results of the SUNLIGHT study validate the use of trifluridine/tipiracil + Bevacizumab association in third line in metastatic colorectal cancer[47,48,49]. Trifluridine/tipiracil plus bevacizumab demonstrated promising efficacy in a randomized phase 2 trial of heavily pretreated patients with metastatic colorectal cancer (mCRC). SUNLIGHT phase II study was conducted to confirm these findings. The global phase 3 SUNLIGHT study enrolled patient aged ≥ 18 years with histologically confirmed mCRC, ECOG PS 0/1, and treated with1-2 prior chemotherapy regimens in an advanced setting, including fluoropyrimidines, irinotecan,oxaliplatin, an anti-VEGF monoclonal antibody (if medically considered) and/or anti-EGFR monoclonal antibody for RAS wild-type tumors. Patients were randomised (1:1) to receive trifluridine/tipiracil (35 mg/m 2 twice daily on days 1–5 and 8–12 of each 28-day cycle) alone or combined with Bevacizumab (5 mg/kg on days 1 and 15).Primary endpoint was overall survival (OS). 492 patients were included between November 2020 and February 2022, randomised to receive trifluridine/tipiracil + Bevacizumab (n = 246) or trifluridine/tipiracil (n = 246). Baseline characteristics were balanced between arms. Trifluridin/tipiracil + Bevacizuma significantly extended OS over Trifluridin/tipiracil alone, median OS was 10.8 months versus 7.5 months, respectively (HR, 0.61; 95% CI, 0.49, 0.77; P, 0.001). OS rates at 12 months were 43% in the Trifluridine/tiîracil + Bevacizumab arm and 30% in the Trifluridine/tipiracil arm. Median progression-free survival was 5.6 months in the trifluridine/tipiracil + Bevacizumab arm and 2.4 months in the trifluridine/tipiracil arm (HR, 0.44; 95% CI, 0.36,0.54; P, 0.001). Grade ≥ 3 adverse events (AEs) were not significantly increased in the trifluridine/tipiracil + Bevacizumab arm versus the trifluridine/tipiracil arm (72.4% vs 69.5%). No new safety signals were noted. The SUNLIGHT study conclusion is that Trifluridine/tipiracil + Bevacizumab provided a statistically significant and a clinically meaningful 3.3-month improvement in OS, extending mOS up to 10.8 months, with a 39% reduction in the HR of death in pts with refractory mCRC and with a predictable and acceptable safety profile. Standard of care in mCRC is being modified since beginning of 2023 according to the practices of each site who applies locally either the treatment Trifluridine/tipiracil alone or in combination with bevacizumab.

In many cancer types, immune-checkpoint inhibitors that target the PD-1/PD-L1 pathways, have changed the face of oncology. Such treatments were shown to improve patient outcomes in several cancer types and to induce a high rate of clinical responses in a subgroup of patients with metastatic microsatellite instability-high (MSI-H) CRC.

Recently, pembrolizumab and nivolumab, alone or in combination with the anti-CTLA-4 ipilimumab, have been approved by the FDA for the treatment of metastatic MSI-H patients (up to 4% of metastatic CRC patients)[6,7]. However, anti-PD-1 therapies given as monotherapy, do not induce clinical responses in microsatellite stable (MSS) patients. Such resistance to immunotherapy can be explained by the reduced expression of neoantigens and the inability of CD8+ T cells to infiltrate the tumor, thus leading to tumor immuno-exclusion[8,9]. However, in MSS colorectal cancer, some CD8 immune cells cancer are detected in both localized and metastatic tumors. Such immune infiltrates are associated with prognosis, suggesting that immune response could shape tumor growth of MSS tumors[10,11]. Alternative strategies to checkpoint inhibitors should be developed to restore antitumor immune response in MSS tumors.

Chronic inflammation is recognized as a promoting factor in carcinogenesis and tumor progression[12]. In the context of CRC, chronic intestinal inflammation, for example inflammatory bowel disease, is associated with colorectal carcinogenesis. For malignant disease, inflammation signals are essential for tumoral processes such as angiogenesis, remodeling of the tumor stroma, tumor invasion, metastatic spread and cachexia. IL-1α and β are major pro-inflammatory cytokines. Polymorphisms in *IL1A, IL1B* and *IL1RA* genes, which induce activation of IL-1 pathway, are associated with a higher risk of tumor recurrence[13]. IL-1β is predominantly expressed on myeloid lineages within tumor microenvironment and plays a role in chronic inflammation. These factors weigh in favor of IL-1β inhibition for cancer prevention or therapy[14]. Conversely, IL-1α is typically expressed by malignant cells[15]. The pro-oncogenic properties of IL-1α are its capacity to induce activation of NFKB and STAT3 pathways and to promote tumor aggressiveness[16]. IL-1 is a key regulator of IL-17 production, which is associated to poor prognosis in colorectal cancer[17,18]. IL-17A blockade reduces tumor progression in preclinical models of colorectal cancer. Genetic deletion of IL-1R1 in epithelial colon cell alleviated tumorigenesis in an APC mice model of colorectal cancer, demonstrating a cell-autonomous role for IL-1 signaling in early tumor seed outgrowth. T cell specific ablation of IL-1R1 decreased tumor-elicited inflammation dependent on IL-17, thereby reducing colorectal

cancer progression. Together, these data underline a role for IL-1 signaling in colorectal progression by acting on tumor induced inflammation and directly promoting colon epithelial cell tumorogenic transformation[19].

When tumors enlarge and the vascularization fails to sufficiently supply them, the tumor cells become stressed (due to hypoxia) and release IL-1α. With continued stress due to hypoxia, the tumor cells undergo necrotic death. The released IL-1α can now trigger the IL-1R1 on neighboring cells. This is the “alarmin” property.[20] Necrotic cells thus evoke inflammation, which brings in neutrophils and inflammatory cells to the area. These effects would be attenuated in the presence of endogenous IL-1Ra. Monoclonal antibody targeting IL1α neutralize the biological activity of IL-1α, including blocking the inflammatory induction of VEGFs and matrix metalloproteinases in order to inhibit neoangiogenesis and stromal remodeling in the tumor microenvironment. But IL-1α is commonly expressed on tumor cells where it is associated with invasiveness and dedifferentiation [21, 22]. Consequently, on tumor cells, IL-1α could act as a target, whereby anti IL1α could mediate direct inhibition of tumors.

Neutralization of IL-1α was proposed to have the potential to reduce tumor growth and to reverse or improve debilitating morbidities associated with the disease in colorectal cancer. MABp1 monoclonal antibody neutralizes IL-1α and improves patient’s symptoms and quality of life when compared to placebo. In an exploratory analysis in a subgroup of patients, the median survival was 6.1 months (95% CI 4.4–7.2) in the MABp1 group compared to only 2.4 months (95% CI 1.9–3.2) in the placebo group, reinforcing the idea that targeting IL-1 pathway is relevant for the treatment of mCRC[23]. Additionally, biological findings in a randomized MABp1 phase III trial in patients with advanced colorectal cancer, have shown that patients with lower levels of circulating IL-1Ra are more responsive to treatment with the IL-1α-targeting antibody MABp1. These observations define a potential biomarker for anti-IL-1α therapy[22]. Recently, we have observed that fluorouracil (5-FU) could activate the NOD-like receptor family, the pyrin domain containing 3-protein (NLRP3)-dependent caspase-1 activation complex (termed the inflammasome) in myeloid-derived suppressor cells (MDSCs), leading to IL-1 production which curtails anticancer immunity. IL-1 was also shown to induce the generation and expansion of T_H17 cells, which could promote tumor growth by favoring proangiogenesis and immunosuppression in an IL-17A dependent manner[25]. These observations were made in mice models but also in patients treated with 5FU. **Based on this rationale we suggested that inhibition of IL-1 could reverse resistance to 5-FU in mCRC.**

We recently performed a phase II clinical trial to test the safety, tolerability and efficacy of an anti IL-1α and IL-1β in combination with 5-FU and bevacizumab in refractory mCRC patients. Eligible patients had unresectable mCRC; were refractory or intolerant to fluoropyrimidine, irinotecan, oxaliplatin, anti-VEGF therapy and to anti-EGFR therapy (for tumors with wild-type *KRAS*). Patients were treated with a simplified folinic acid plus 5-FU regimen and bevacizumab (5 mg/kg) both administered by intravenous infusion every 2 weeks. XB2001 was injected subcutaneously once daily. The primary endpoint was the 2-months response rate determined upon CHOI criteria. Thirty-two patients with metastatic colorectal cancer were enrolled. Five patients demonstrated a response (CHOI criteria) and 22 patients had stable disease as the best 2-months overall response. Median progression-free and overall survival were 5.4 (95% CI, 3.6–6.6) and 14.5 months (95% CI, 9–20.6) respectively. Twenty patients experienced grade 3 toxicity. No grade 4 or 5 toxicity related to therapy occurred. The most common grade 3 adverse events were neutropenia in 8 (25%) patients, digestive side effects in 7 (21.9%) patients and hypertension in 6 (18.75%) patients. No treatment-related deaths or serious adverse events were reported**. 5-FU plus bevacizumab and anti IL-1α and IL-β has promising activity and a manageable safety profile, suggesting that this combination might become a potential treatment option for patients with refractory mCRC[26].**

In a recent unpublished preclinical work, we tested the immune effect of trifluridine/tipiracil and observed that, like 5-fluorouracil, this drug could induce blood monocytic MDSCs depletion. Trifluridine/tipiracil could also deplete tumor associated macrophages and induce IL-1 release by dying macrophages and monocytic MDSCs. **Such data support the rationale to test combination of trifluridine/tipiracil plus XB2001 in advanced chemorefractory colorectal cancer.**

XBiotech USA, Inc. has identified a True Human monoclonal antibody, XB2001 that blocks the biological activity of IL-1α with a high degree of affinity and specificity. IL-1α is a key and perhaps ubiquitous mediator of inflammatory responses and is implicated in the pathophysiology of various diseases, including cancer, cardiovascular and rheumatologic diseases. A vast body of clinical and non-clinical evidence supports targeting IL-1α to block pathological inflammatory processes that underlie an array of diseases. XB2001 is a recombinant human IgG4 monoclonal antibody specific for human interleukin-1α (IL-1α).

The entire XB2001 heavy and light chain sequences are identical to those found in naturally-occurring human peripheral repertoire, originally expressed by a peripheral blood B lymphocyte obtained from a healthy anti-IL-1α immune individual. No in vitro affinity maturation or modifications have been made to improve its natural binding affinity (~60 pM). True human antibodies, such as XB2001, should be effectively non-immunogenic in humans and thus be optimal in terms of safety, activity and pharmacokinetics. XBiotech has conducted 15 clinical studies to date using a previous anti-IL-1α True Human antibody[27, 28, 29, 30, 31, 32, 33, 34, 35]. These studies were conducted in a range of different therapeutic areas, from cancer to healthy volunteers, and have included intravenous and subcutaneous formulations and several different dose levels and dosing schedules. Targeting IL-1α has been found to be safe and very well tolerated in all studies [31, 32, 33, 36].

A first-in-man phase I study and two phase III studies sponsored by XBiotech have been completed with a previous generation anti-IL-1α True Human monoclonal antibody in oncology: (Advanced Cancers NCT01021072, Advanced Colorectal Cancer NCT01767857, and symptomatic colorectal cancer NCT02138422)[31,32]. The schedule dosage was 7.5mg/kg every 2 weeks.

XB2001 is a naturally occurring antibody that neutralizes IL-1α and is thus a safe and promising approach to block inflammation that occurs with advanced malignancies and chemotherapy. IL-1α induces upregulation of VEGF and angiogenesis in the tumor microenvironment; IL-1α also acts to recruit infiltration by leukocytes (such as myeloid suppressor cells) that can suppress anti-tumor immunity; systemically, IL-1α can mediate metabolic dysregulation, fatigue, anorexia, and anxiety through the hypothalamic-pituitary-adrenal axis. IL-1α is thus a central player in paraneoplastic inflammation. A Phase I-II was developed in pancreatic cancer using XB2001 plus 5-Fluorouracil, leucovorin and liposomal irinotecan. Three doses are bring tested (250mg, 500mg and 1000mg flat dose). The maximum dose studied was 1000mg.

XB2001 is the second molecule developed by XBiotech to specifically target interleukin-1α (IL-1α). By targeting a key pathway in paraneoplastic inflammation, previous clinical findings targeting IL-1α has shown anti-tumor activity while at the same time treating disease-related symptoms. The highly regulated expression of IL-1α makes

it a safe target with excellent tolerability profile, and thus an ideal treatment option in a vulnerable advanced cancer population, and highly suitable as an adjunct in combination with cytotoxic therapies.

1.2 Originality and innovative aspects of this project

Targeting IL-1 is an emerging field in oncology. IL-1 could directly promote tumor growth or impede anti-tumor immunity. Ongoing trials, performed in the setting of lung and breast cancer, test the effect of canakinumab (an IL-1β inhibitor) alone or in addition with chemotherapy or immunotherapy (NCT03447769, NCT03968419, NCT03631199, NCT03626545, NCT03742349).

In addition, drugs like trifluridine/tipiracil or 5FU induce production of IL-1 by myeloid cells such as monocytic MDSC and tumor associated macrophages[26] (and unpublished data). This effect limits fluoropyrimidin antitumor efficacy in preclinical models. Similar results were observed in patients with mCRC. These data support a strong rationale to combine fluoropyrimidine and an IL-1 blocker in mCRC cancer. Our previous trial demonstrated the safety of 5FU combination with XB2001 in mCRC cancer and strongly suggested efficacy[26].
Thus, based on clinical trials using IL-1 inhibition alone and our clinical trial combining 5-fluororuracil with anti IL-1 we believe that a randomized trial comparing trifluridine/tipiracil versus trifluridine/tipiracil plus XB2001 will be important to confirm the potential efficacy of fluoropyrimidin and IL1 blockers combination.

1.3 Patient benefits and foreseeable risk(s) Expected results

1.3.1. Potential benefit

mCRC is a frequent disease in France and western Europe. After failure of classical therapies few therapeutic options are available. XB2001 is expected to have a good safety profile, and a previous generation True Human anti-IL-1α has been shown to be safe and effective in several clinical studies of patients who have not responded to standard chemotherapy. If such treatment could improve efficacy of third line therapy in mCRC and improve quality of life such discovery will have direct beneficial effect on patients and public health.

1.3.2. Potential risk

The most serious side effects seen in patients receiving trifluridine/tipiracil are myelosuppression and gastrointestinal toxicity. The most commonly observed side effects (≥ 30%) in patients receiving trifluridine/tipiracil are neutropenia (53% [34% ≥ Grade 3]), nausea (34% [1% ≥ Grade 3]), fatigue (32% [4% ≥ Grade 3]), anemia (32% [12% ≥ Grade 3]). The most common adverse reactions (≥ 2%) resulting in discontinuation of treatment, dose reduction, postponement or interruption of administration are neutropenia, anemia, leukopenia, fatigue, thrombocytopenia, nausea and diarrhea.

Few risks are expected with trifluridine/tipiracil +/- XB2001. Major risks are an increased incidence of infection or myelosuppression and proteinuria. The previous study performed by the CGFL (IRAFU, NCT02090101) with the association of Anti IL-1α and β and a chemotherapy based on 5-FU +/- bevacizumab on the same population, did not show an increase in toxicity or adverse effects due to the combination therapy. The risk benefit balance of this study is acceptable.

1.4 Study population

Patients with advanced multitreated metastatic colorectal cancer.

2. STUDY OBJECTIVES

2.1 Main objectives

2.1.1. Main objective of phase I portion

The main objective of the phase I study is to establish the Maximum Tolerated Dose (MTD) of XB2001 (anti-IL-1α True Human antibody) as measured by Dose-Limiting Toxicity (DLT) in combination with trifluridine/tipiracil +/- bevacizumab in patients with refractory metastatic colorectal cancer.

2.1.2. Main objective of phase II portion

The main objective of the study is to evaluate the efficacy of trifluridine/tipiracil +/- bevacizumab + XB2001 (Experimental Arm) in comparison with trifluridine/tipiracil +/- bevacizumab + placebo (Standard Arm) in term of **6-month overall survival** in patients with refractory metastatic colorectal cancer.

2.2 Secondary objectives

2.2.1. Secondary objective of phase I

1) Pharmacokinetics (PK) study.

2) Pharmacodynamics (PD) study. In order to evaluate the decrease of IL-6 serum level

3) Safety of the association of XB2001 (anti-IL-1α True Human antibody) as measured by Dose-Limiting Toxicity (DLT) in combination with trifluridine/tipiracil +/- bevacizumab. Bevacizumab will be administered at the discretion of each investigator.

2.2.2. Secondary objective of phase II

1) To evaluate efficacy in both arms in terms of:

- Objective response rate (2 and 4-month rates) (RECIST 1.1);
- Progression free survival (PFS) (2-month, 4-month, 6-month rates and median PFS);
- Median OS

2) To evaluate efficacy (objective response rate, PFS and OS) according to mutational status, tumor sideness and administration of bevacizumab

3) To evaluate the tolerance in both arms;

4) To evaluate the quality of life in both arms.

2.3 Exploratory objectives of phase I and II

1) To perform an exhaustive peripheral immunological biomarker study before and under therapy (inflammatory cytokines (Luminex), immunosuppression (Treg MDSC), specific T cell response, peripheral CD8 and CD4 T cells phenotyping (polarization and exhaustion phenotyping), CD8 and CD4 T cell global function using intracellular staining).

2) To evaluate in situ immune response using immunohistology in a tumor biopsy before treatment

3) To evalutate inflammatory parameters (IL1RA, IL1A, IL1B, IL6, IL33)

4) To evaluate tumor markers and circulating DNA kinetics as early biomarkers of response.

5) To evaluate pharmacokinetics (PK) of trifluridine with or without XB2001

6) To evaluate in situ immune response using immunohistology in a biopsy performed 2 months after the initiation of treatment.

3. EVALUATION CRITERIA

3.1 Primary end-points

3.1.1 Primary end-points of phase I portion

The Maximum Tolerated Dose (MTD) of XB2001 is defined as the highest dose level at which no more than one out of six patients experience a DLT. If two or more out of six patients within the same cohort encounter DLT, MTD will be exceeded and the lower dose level will be considered to be the MTD. Adverse events (AEs) will be defined by Common Terminology Criteria for Adverse Events (CTCAE) version 5.0. Recommended Phase II Dose (RP2D) will be establish based on the MTD and the PK-PD relationships (between XB2001 exposure metrics (Cmax or trough concentrations) and IL-6 decrease. If no toxicity occurs at the maximum tested dosage, the RP2D dose will be determined by IDMC based on PK-PD data. IDMC will validate the RP2D. In total, 6 patients should be treated at the RP2D.

DLT is defined as all grade 3-4 adverse events that are deemed related to the test article unless there is a clear alternative cause. The observation period for DLT is set at the first 28 days following the first XB2001 administration

3.1.2 Primary end-points of phase II

Efficacy will be evaluated using 6-month overall survival. Overall survival is defined as the time interval from randomization to the date of death from any cause. Patients alive will be censored at the last date of last known follow-up.

3.2 Secondary endpoints

3.2.1 Secondary endpoints of phase I

1) Pharmacokinetics will be asses by, serum and plasma samples collected before XB2001 administration and at 6 time points post administration:

1- day 1 before infusion
2- day 1 ≈ 30 minutes post infusion (+/-10 minutes)
3- day 2
4- day 5
5- day 8
6- day 15
7- day 29

PK measurement will be carried out by quantitative indirect ELISA for XB2001 (Anti IL-1α) concentration measurement in Plasma.

2) Pharmacodynamics (PD) study will evaluate the decrease of IL-6 in serum level using ELISA (test performed D1, D15 and D29)

3) Tolerance and Safety of bevacizumab addition will be evaluated according to the CTCAE V 5.0. All adverse effects will be reported and graded.
DLT are defined as all grade 3-4 adverse events that are deemed related to bevacizumab unless there is a clear alternative cause. The observation period for DLT is set at the first 28 days following the first XB2001 + trifluridine/tipiracil + bevacizumab administration.
In level 5, XB2001 will be at the dose of level 4 if no toxicities observed during this level or at level 3 if toxicities are observed in level 4 and lower level is retained for MTD of XB2001 evaluation.
In level 5, no more than one out of six patients should experience a DLT related to bevacizumab or unless clear alternative cause. If two or more out of six patients within the level 5 encounter a DLT, the association with bevacizumab will not be retained for the phase II.

3.2.2 Secondary endpoints of phase II

1) **Objective response** will be defined according to RECIST 1.1 criteria, at 2 and 4 months.

Progression-free survival (PFS). PFS is defined as the time interval from randomization to the date of progression (defined using RECIST 1.1 criteria) or death from any cause. Patients alive without progression will be censored at the last date of last known follow-up. Median PFS as well as PFS rates at 2, 4 and 6-months will be provided.

2) Overall survival (OS), PFS (median, 2 4 and 6 months-rates) and response rate (2 and 4-month rates) will be described according to RAS status, right or left side of the tumor and administration of bevacizumab or not.
   - **OS** is defined as is defined as the time interval from randomization to the date of death from any cause
   - **PFS** is defined as the time interval from randomization to the date of progression.

Response and progression will be identified according to the RECIST criteria. Radiological responses will be evaluated by the investigator but also in central review.

3) **Tolerance** will be evaluated according to the CTCAE V 5.0. All adverse effects will be reported and graded at each visit.

4) **Quality of life** will be evaluated with the survey from EORTC: QLQ-C30 and QLC-CR29. Surveys will be completed at baseline (inclusion) and every two cycles until progression + at progression.

3.3 Exploratory endpoint of phase I and phase II

1) Peripheral markers: serum determination of inflammatory cytokines, CD4 polarization, CD8 phenotyping, functional assay of CD8 and CD4 T cells.
2) Tumor markers: lymphocyte infiltrate by immunohistochemistry (CD3, CD8, PDL1, CD163, DC-Lamp, PNAD, CD20).

3) Inflammtory markers (IL1RA, IL1A, IL1B, IL6, IL33)

4) Liquid markers (tumor marker CEA; Circulating DNA)

5) Analysis of trifluridine plasma concentrations (in ng/mL) by means of appropriate software to calculate the typical values and variabilities of trifluridine PK parameters in the study population by a population approach (only for the phase II)

6) Tumor markers: lymphocyte infiltrate by immunohistochemistry (CD3, CD8, PDL1, CD163, DC-Lamp, PNAD, CD20).

4. STUDY DESIGN

4.1 Study methodology

This trial will include 2 parts:

The first part will be a phase I, open label, 3+3 dose escalation study evaluating the safety, tolerability and establishing the Maximum Tolerated Dose (MTD) of XB2001 in a least 15 patients with metastatic colorectal cancer who are receiving trifluridine/tipiracil chemotherapy for levels 1 to 4 and in association with bevacizumab for level 5.. The duration of each patient in the phase I portion will be until progression of the disease (approximately 6 months) or unacceptable toxicities.

The second portion will be a phase II implemented following the completion of the phase I portion and declaration of the MTD. The duration of subject participation in the randomized, double-blind, placebo-controlled phase II portion of the Trial is approximately 6 months, until disease progression or unacceptable toxicities.

4.1.1. Phase I design (Dose escalation phase)

4 differents doses levels of XB2001 will be investigated:

- XB2001 250 mg alone (**level 1**) followed by level 2 at the end of the DLT period
- XB2001 250mg + trifluridine/tipiracil (concomitant administration) (**level 2**);

Intra patient escalation between level 1 and 2. No intra patient is planned for other dose levels.

- XB2001 500mg + trifluridine/tipiracil (concomitant administration) (**level 3**)
- XB2001 1000mg + trifluridine/tipiracil (concomitant administration) (**level 4**)

Level 5 will consist in either:

- XB2001 1000mg + trifluridine/tipiracil **+ Bevacizumab** (**level 5**) if less than 33% of toxicities occur at level 4
- XB2001 500mg + trifluridine/tipiracil **+ Bevacizumab** (**level 5 bis**) if 33% or more toxicities occur at level 4

Intra patient escalation is planned between level 1 and level 2. No intra patient is palnned for other dose levels Please refer to the diagram below of phase I:

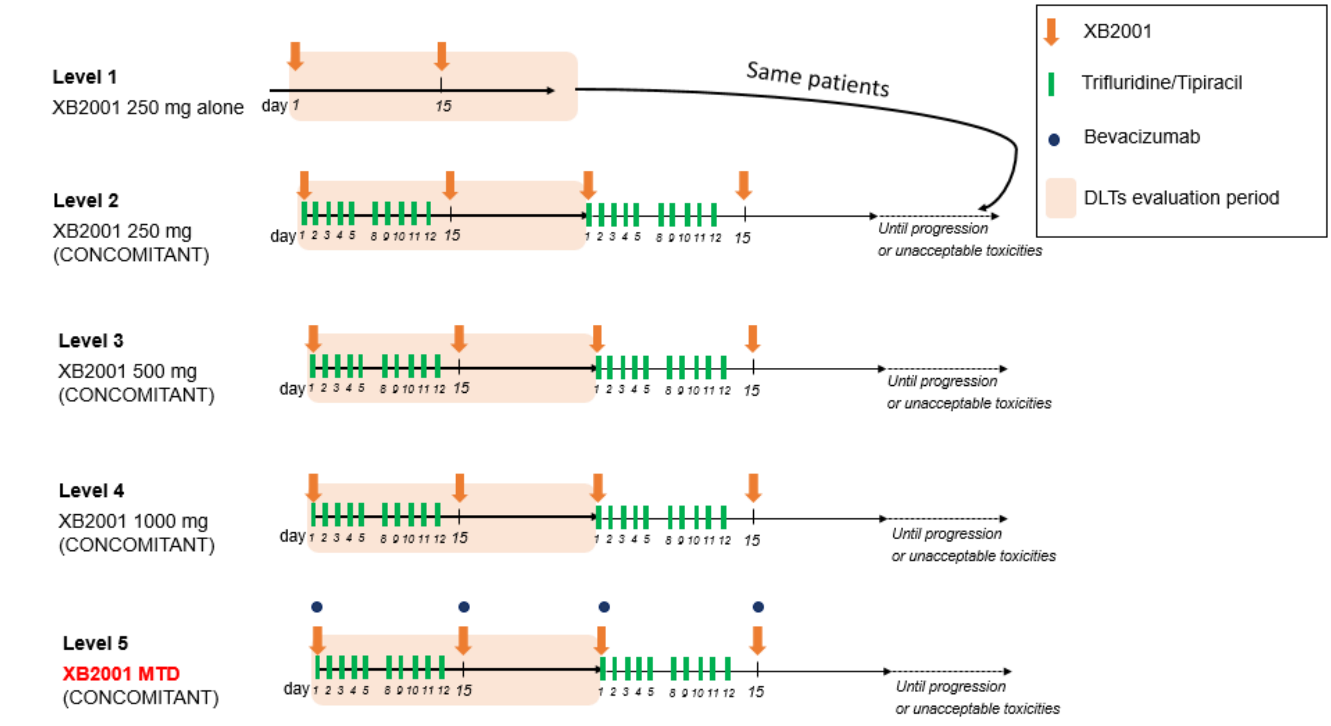

<u>Dose Escalation Rules</u>:

- XB2001 will be dose escalated with a 3+3 design used in order to establish MTD. The dose of XB2001 will be escalated in sequential cohorts of three patients each. Dose-escalation will continue if none of three patients experience a dose-limiting toxicity (DLT) (defined below). First patients of the level 1, 2 and 3 would be included two by two. Unless a DLT is observed, patients will only be included one by one. For the level 4 and 5, patients would be included three by three as is it prevous for a standard design of 3+3.
- The first level will be a monotherapy with of XB2001
- Subsequent levels will consist in increased doses of XB2001 + trifluridine/tipiracil (concomitant)
- A dose level will be expanded to 3 additional patients if one of the first three patients experience a DLT during the first treatment cycle of trifluridine/tipiracil alone (28 days – 2 administrations of XB2001) for level 1, 2, 3, 4. Same rules for the level 5 with the addition of bevacizumab.
- Due to the fact that the trifluridine/tipiracil chemotherapy regimen is an established treatment for these patients only the dose for XB2001 will be changed.
- Enrollment to dose-cohort levels 3, 4 and level 5 will be withheld until the last enrolled patient in the previous dose-cohort has reached the end of DLT evaluation period.
- 6 patients should be treated at the MTD
- The maximum tolerated dose (MTD) of XB2001 is defined as the highest dose level at which no more than one out of six patients experience a DLT. If two or more out of six patients within the same cohort encounter DLT, MTD will be exceeded and the lower dose level will be considered to be the MTD. Adverse events (AEs) will be defined by Common Terminology Criteria for Adverse Events (CTCAE) version 5.0.
- Patients evaluable for MTD must either have received at least 80% doses of trifluridine/tipiracil (for patients in level 2, 3, 4 and 5) and 2 injections of XB2001 or have experience a DLT.

- DLT is defined as all grade 3-4 adverse events that are deemed related to the test article unless there is a clear alternative cause. The observation period for DLT is set at the first 28 days following the first XB2001 administration
- Patients who do not finish the first cycle due to AEs other than DLTs will be replaced. Events will be classified as Not suspected (the relationship of the AE to study drug made a causal relationship unlikely or remote, or other medications, therapeutic interventions, or underlying conditions provided sufficient explanation for the observed event) or Suspected (the relationship of the AE to study drug made a causal relationship possible, and other medications, therapeutic interventions, or underlying conditions did not provide sufficient explanation for the observed event).
- All AEs will be recorded by the Investigator(s) during the period between post administration of the first dose of treatments until 14 days following the last dose of study drug administration.
- At each dose level, PK (Cmax, Cmean, Cmin), PK parameters (Cl, Vd,…) and accumulation will be determined.
- PD (variation of IL-6 level) will be determined at D1, D15 and D29.
- At the end of dose level 2, 3, 4 and 5 IDMC will analyse PK/PD data in addition to MTD data to validate the dose level
- Level 5. Dose of XB2001 will be the dose selected at the last level tested.
  - ➔ Level 4 if no DLT observed or 1 DLT over 6 patients
  - ➔ Level 3 if more than 1 DLT over 6 patients have been observed at level 4.
- If at the level 5 more than 1 DLT are observed and related to bevacizumab over 6 patients, association with bevacizumab will not be retained for the phase II of the study.

Patients in the phase I of the study will be treated until disease progression, unacceptable toxicity, intercurent conditions that preclude continuation of treatment or patient refusal in the absence of progression or intolerance.

After the MTD has been declared, the study will progress to the Phase II portion.

An efficacy IDMC will held after the inclusion in the phase II of 52 patients (26 in each arm). If the efficacy of the treatment is proven at the end of the IDMC, patients of the phase I still in the treatment period at a lower dose from the MTD will be informed of the result of the efficacy and will be able to benefit from the MTD dose.

4.1.2. Phase II design (Dose expansion phase)

This is a multicenter, randomized (1:1 ratio), double blind, non- comparative, phase II-study with a standard arm (Arm A) which consists of trifluridine/tipiracil + placebo and a experimental arm (Arm B) consisting of trifluridine/tipiracil in association with XB2001 in patient with advanced multitreated metastatic colorectal cancer.

Patient will be treated until disease progression, unacceptable toxicity, intercurrent conditions that preclude continuation of treatment or patient refusal in the absence of progression or intolerance.

- **ARM A – Standard arm – standard of care** (trifluridine/tipiracil +/- bevacizumab) **+ placebo**: Trifluridine/tipiracil: 35 mg/m² bid d1-d5; d8-d12; 1 cycle every 28 days +/- bevacizumab 5mg/kg every 2 weeks . Placebo administration every 2 weeks

- **ARM B – Experimental arm – standard of care** (trifluridine/tipiracil +/- bevacizumab) **+ XB2001**: Trifluridine/tipiracil: 35 mg/m² bid d1-d5; d8-d12; 1 cycle every 28 days +/- bevacizumab 5mg/kg every 2 weeks. Intra venous administration of XB2001 at 250mg, 500mg or 1000mg (dose selected according to the results of phase I) every two weeks.

4.2 Progression of the study

The study will consist of a 21-day baseline phase. Treatment phase will be with 28-day cycles of trifluridine/tipiracil +/- XB2001 until progression or until the development of unacceptable toxicity. After the end of treatment, there will be a 24-month post treatment phase (follow-up) which includes safety, efficacy and survival follow-up.

The investigation/procedures and examination schedule is shown in the study flow-chart in table 1 at the begining of the protocol.

The end of the study is defined as the date of the last visit performed by the last patient in the study.

The overall study duration is estimated to be approximately 54-month (24 month of recruitment + a median of 6-month of treatment + 24-month follow-up phase).

4.3 Inclusion and registration procedures

After eligibility criteria have been checked and fulfilled and the patient's consent has been obtained for principal study and facultative ancillary studies, the patient will be included and registered in the study.

The principal investigator or the CRA delegated for this task will performed the enrolment and randomization in the eCRF Clinsight. In return, the site will receive via email the confirmation of the inclusion of the subject with the allocated inclusion number. For the Phase II, the ARM of treatement XB2001 or placebo will only be provided to the pharmacy that is not blinded. The site staff and investigator will be blinded from the treatement arm and will only received the inclusion number. In case of problem with the eCRF Clinsight during enrolment please contact the data management department of the Centre Georges-François Leclerc. Contact details are summarized below:

Unité de méthodologie, Biostatistiques et Data Management
From Monday to Friday from 9 A.M to 6 P.M
Phone: +33(0)3.45.34.80.90 Fax: +33(0)3.80.73.77.34
Patients will begin treatment immediatly following registration/randomization.

4.4 Study early terminations/stopping rules and patient premature discontinuation

4.4.1 Temporary discontinuation and definitive termination of the study

The study can be suspended or stopped by the sponsor in agreement with the coordinating investigator, at the request of the IDMC and/or following a request by the respective regulatory authority and/or the responsible Ethics Committee for the following reasons:

- High frequency and/or unexpected severity of toxicity
- Insufficient patient enrolment
- Insufficient quality of data collection

4.4.2 Withdrawal from the study

Subjects are at any time free to withdraw from the study (investigational product and assessments), without prejudice to further treatment (withdrawal of consent). The reason(s) for withdrawal and the presence of any AEs will be investigated. If possible, they will be seen and assessed by an investigator. The investigator will follow-up AEs outside of the clinical study, until resolution.

4.4.3 Withdrawal from the study – withdrawal of consent

Patients who refuse to continue participation in the study, including telephone contact, should be documented as "withdrawal of consent" rather than "lost to follow-up."

Patients who withdraw consent for further participation in the study will not receive any further IP or further study observation, with the exception of follow-up for survival, which will continue until the end of the study unless the patient has expressly withdrawn his or her consent to survival follow-up. Note that the patient may be offered additional tests or tapering of treatment to withdraw safely.

If a patient withdraws from participation in the study, then his or her patient identification number cannot be reused. Withdrawn patients will not be replaced.

4.4.4 Discontinuation from study treatment

- <u>Criteria for discontinuation of a patient from study treatement</u>

A patient will have a permanent discontinuation of investigational product and will not receive any further investigational product if any of the following occur:

1. Withdrawal of consent from the study or lost to follow-up.

2. Withdrawal of consent from further treatment with IP: The patient is, at any time, free to discontinue treatment, without prejudice to further treatment. A patient who discontinues treatment is normally expected to continue to participate in the study unless they specifically withdraw their consent to further participation in any study procedures and assessments.

3. Adverse event that, in the opinion of the investigator or the sponsor, contraindicates further dosing.

4. Patient is determined to have met one or more of the exclusion criteria for study participation at study entry and continuing investigational therapy might constitute a safety risk.

5. Pregnancy or intent to become pregnant.

6. Patient noncompliance that, in the opinion of the investigator or sponsor, warrants withdrawal; e.g., refusal to adhere to scheduled visits.

7. Initiation of alternative anticancer therapy including another investigational agent.

8. Confirmation of PD and investigator determination that the patient is no longer benefiting from treatment. Patients who are permanently discontinued from further receipt of investigational product, regardless of the reason (withdrawal of consent, due to an AE, other), will be identified as having permanently discontinued treatment.

9. Patients who are permanently discontinued from receiving investigational product will be followed for safety, including the collection of any protocol-specified blood specimens, unless consent is withdrawn, or the patient is lost to follow-up or enrolled in another clinical study. All patients will be followed for survival. Patients who decline to return to the site for evaluations will be offered follow-up by phone as an alternative.

10. If platelets <60 000 mm^3 after 2 weeks of trifluridine/tipiracil interruption, XB2001 will be stopped and patient will perform end of study visit.

- <u>Procedures for discontinuation of a patient from study treatement</u>

At any time, patients are free to discontinue IP or withdraw from the study, without prejudice to further treatment. When a patient discontinues treatment for reasons other than disease progression, the sponsor (centre Georges-François Leclerc) should be informed immediately. The reason(s) for discontinuation IP and the presence of any AEs will be investigated If possible, they will be seen and assessed by an investigator(s). All AEs will be followed up and all IP should be returned by the patient.

By discontinuing from treatment, the patient is not withdrawing from the study. Patients should be followed for progression (if discontinuation in the absence of progression) and OS following treatment discontinuation as per the protocol schedule.

Any patient discontinuing IP should be seen within 14 days post-discontinuation for the evaluations outlined in the study schedule. The patient’s tumor status should be assessed clinically and, if appropriate, disease progression should be confirmed by radiological assessment. After discontinuation of IP, the PI/Sub-investigator will perform the best possible observation(s), test(s) and evaluation(s) as well as give appropriate medication and all possible measures for the safety of the patient. In addition, they will record on the eCRF the date of discontinuation, the reasons, manifestation and treatment at the time of discontinuation. If patients discontinue IP, the monitor must be informed immediately. Patients will be required to attend the treatment discontinuation visit. The patient should return all IP.

After discontinuation of the IP at any point in the study, all ongoing AEs or SAEs must be followed until resolution unless, in the investigator’s opinion the condition is unlikely to resolve due to the patient underlying disease, or the patient is lost to follow-up. All new AEs and SAEs occurring during the 30 calendar days after the last dose of IP must be reported (if SAEs, they must be reported to UNICANCER PV within 24 hours as described in Section 10.4) and followed to resolution as above. Patients should be seen within 14 days after discontinuing IP to collect and/or complete AE information.

Any patient who has not yet shown objective radiological disease progression at withdrawal from IP should continue to be followed as per RECIST 1.1.

All patients will be followed for disease progression and survival until 24 months after the end of treatement.

Regardless of the reason for termination, all data available for the patient at the time of discontinuation of follow-up must be recorded in the eCRF. All reasons for discontinuation of treatment must be documented.

4.4.5 Patient lost to follow-up

Patients will be considered lost to follow-up only if no contact has been established by the time the study is completed, such that there is insufficient information to determine the patient’s status at that time.

Investigators should document attempts to re-establish contact with missing subjects throughout the study period provided they have not withdrawn consent. If contact with a missing subject is re-established, the subject should not be considered lost to follow-up and any evaluations should resume according to the protocol.

4.4.6 Screen failures patients

Screen failures patients are patients who do not fulfill all the eligibility criteria for the study and therefore must not be assigned to treatment and must not be registered in the eCRF. In case those patients have been created in the eCRF, these patients should have the reason for « end of study » study withdrawal - ‘Incorrect Enrollment’ (ie, patient does not meet the required inclusion/exclusion criteria). This reason for study withdrawal is only valid for screen failures (not enrolled patients) and will be recorded on the screen failure log.

4.4.7 Replacement of patients

The main analysis of the efficacy criteria will be evaluated on mITT population (all the patients included in the study whatever the inclusion/non-inclusion criteria and who received at least one dose of treatment). A rate of 5% non-evaluable patient was taken into account in the sample size determination. Patients will not be replaced except if we observed more than 5% of non-evaluable patient (i.e. without at least one dose of treatment) at the end of the study.

5. PATIENT SELECTION

5.1 Inclusion criteria

The following criteria will be verified during the baseline phase (pre-screening period of 21 days) before patient registration. The informed consent form must be signed before any study-related procedure is performed.

To be eligible, the patients must fulfil all of the following inclusion criteria:

1. Male or female that must have signed a written informed consent prior to any study specific procedures
2. Aged ≥ 18 years at randomization
3. Patient with histologically proven metastatic colorectal cancer previously treated for metastatic disease by chemotherapy treatment including oxaliplatin, irinotecan, fluoropyrimidine, antiangiogenic (anti-VEGF: bevacizumab or aflibercept) and anti-EGFR (cetuximab or panitumumab) if indicated. For MSI tumor patients must be prereated by anti PD1 or anti PDL1 therapy.
4. Have a performance status of 0 or 1 according to the WHO Easter Cooperative Oncology Group (ECOG)
5. Knowledge of RAS, BRAF, Microsatellite status (MSS/MSI)
6. Baseline tumoral evaluation (thoraco-abdomino-pelvic computed tomography) perfromed within 21 days before inclusion/randomization with at least one measurable lesion according to RECIST 1.1 criteria.
7. Patient willing and able to comply with protocol for the duration of the study including: scheduled visits and exams, visits during the follow-up and treatment compliance.
8. Adequat hepatic, renal and bone marrow function within the following limits:
    - Total bilirubin ≤ 1,5 times the upper limit of normal (ULN) (unless documented Gilbert’s syndrome);
    - ASAT et ALAT ≤ 5 times ULN;
    - Measured Creatinine clearance (Cockcroft and Gault) > 30 ml / min
    - Absolute Neutrophil Count (ANC) > 1,5. 10^9 / L;
    - Platelet count ≥ 150. 10^9 / L;
    - Haemoglobin ≥ 9 g / dL (patients can be included even if they have been transfused)
    - Albuminemia ≥ 30 g / L;
    - Negative Hepatitis B, C and HIV serologies, or absence of active B or C hepatitis
    - Urea protein, urine dipstick should be less than 2 crossese or <1g/kg

9. Availability of tumor material dated less than 2 years with sufficient quantity (15 to 20 whithe slides)
10. Patient must be affiliated to a social health insurance
11. Evidence of post-menopausal status or negative urinary or serum pregnancy test for female pre-menopausal patients (urine within 72h or serum pregnancy within 14 days prior to inclusion).

12. - Women of childbearing potential willing to use adequate contraception method (including the use of a mechanical method of contraception in the event of hormonal contraceptive treatment) during the treatment period and 6 months following the end of treatment.

- Male patients with a partner of childbearing potential should use effective contraception during treatment and for up to 6 months after stopping treatment.

13. Normal ECG or ECG withour clinically significant findings with QTc < 470 ms.

5.2 Non inclusion criteria

Patient must not be enrolled if he/she fulfils any of the following non-inclusion criteria:

1. Other concurrent malignancies the last 3 years, except adequately treated cone-biopsied in situ carcinoma of the cervix, basal cell, squamous cell carcinoma of the skin or low risk prostate cancer. Patient who have had potentially curative therapy for a prior malignancy are eligible provided there has been no evidence of disease for ≥ 5 years and the risk of recurrence is considered low.
2. Symptomatic brain metastases
3. Estimated prognosis <3 months.
4. Mutational status BRAF mutant
5. Participation in progress, or in the 30 days preceding the first scheduled day of dosing in this study, in another therapeutic trial with an experimental molecule or within a time interval less than at least 5 half-lives of the investigational agent, whichever is longer.

6. Severe unbalanced illness, underlying infection that may prevent the patient from receiving treatment. Patients with a clinically important and unresolved Grade 3 or 4 non-haematologic adverse reaction related to previous therapies. Also, participant with any known Grade 3 or 4 anemia, neutropenia or thrombocytopenia due to prior chemotherapy that persisted > 4 weeks and was related to the most recent treatment.

7. Bowel obstruction or sub-obstruction or a history of inflammatory bowel disease or significant gasto intestinal disorder

8. History of autoimmune or inflammatory disease or interstitial lung disease.

9. Patient with congenital galactosemia, total lactase deficiency (lactose intolerance) or glucose-galactose malabsorption syndrome

10. Severe arterial thromboembolic events less than 6 months before randomization

11. New York Heart Association (NYHA) Class III or IV congestive heart failure, ventricular arrhythmias or uncontrolled blood pressure (defined as ≥ 160/100 mm Hg)

12. Clinically significant decrease in performance status (medical records) within 2 weeks of intended first dose administration.

13. Contraindication to receive a treatment with trifluridine/tipiracil, or an anti-IL-1α (XB2001 True Human antibody)

14. Concomitant systemic treatment with immunotherapy, immunosuppressants, corticosteroid therapy ≥10mg equivalent prednisone/prednisolone or hormone therapy: corticosteroid therapy administered chronically, immunosuppressive treatment, biotherapy administered as part of the management of an inflammatory disease (anti-TNF, anti-IL6, anti-IL1, anti PD-1, anti EGFR etc.) and live virus vaccines administered up to 14 days prior the first scheduled dose of treatement administration.

15. Current pregnancy (mendatory pregnancy test at baseline for female of childbearing potential) or breastfeeding.

16. Patient with any psychiatric, psychological, sociological, geographical problem or other severe concomitant disease, disorder or condition that potentially compromising the understanding of the information, the safety of the patient, the interpretation of study results or the conduct of the study compliance with the study protocol and follow-up schedule.

17. Patient deprived of their liberty or under guardianship, curatorship or safeguard of justice.

18. Known or suspected history of immunosuppression, including history of invasive opportunistic infections (e.g tuberculosis, histoplasmosis, listeriosis, coccidioidomycosis, pneumocystosis, aspergillosis) despite infection resolution. Presence or suspicion of active bacterial, fungal or viral infections, or uncontrolled fever.
19. Major surgery within 2 weeks prior to randomization, or have an unhealed operation wounds.

Only applicable if standard of care will require bevacizumab administration
20. Uncontrolled wound and important surgery within the last 28 days
21. Uncontrolled brain metastases (by local treatment)
22. All uncontrolled progressive disease within 1 month prior to randomization: grade 3-4 gastrointestinal bleeding (peptic ulcer, erosive esophagitis or gastritis), infectious disease or intestinal inflammation, diverticulitis, pulmonary embolism or other uncontrolled thromboembolic event
23. Uncontrolled high blood pressure, defined as a systolic blood pressure >140 mmHg or diastolic pressure >90 mmHg
24. Patients receiving anticoagulant treatment with an unstable dose of a vitamin K antagonist treatment, and/or having an abnormal INR (>3) in the four weeks before the randomization
25. Verified proteinuria above or equal to 1g/24 hours measured from 24 hours of urine if the urinary protein dipstick control is above or equal to 2+
26. Creatinine clearance (MDRD) <50 ml/min
27. Hypersensitivity to the active substance or to any of the excipients of bevacizumab or any contre indication to receive a treatment by bevacizumab
28. Hypersensitivity to Chinese Hamster Ovary (CHO) cell products or other recombinant human or humanised antibodies

6. STUDY TREATMENTS

The treatment to be used in this study are outlined in the table below
IMP = Investigational medicinal product
NIMP = Non investigational medicinal product

**Drug name**	**Dose / posology**	**Pharmaceutical form**	**Route of administration**	**IMP /NIMP**	**Sourcing**
**XB2001**	250 mg, 500 mg or 1000 mg based on phase I results - Q2W	Glass vials containing 10mL of 100mg/mL	IV infusion	IMP	Provided by the sponsor
**XB2001 Placebo**	250 mg, 500 mg or 1000 mg based on phase I results - Q2W	Glass vials of 10mL containing isotonic solution of PH ~7	IV infusion	IMP	Provided by the sponsor
**Trifluridine/tipiracil - TAS102 (LONSURF®)**	35mg/m^2<br>Twice a day from day 1 to day 5 and from day 8 to day 12. The cycles will be repeated every 28 days<br>Capsules of 15 or 20 mg.	Capsules of 15 mg and 20 mg	Oral administration	NIMP	Provided locally by the study site.
**Bevacizumab**	Solution for infusion	5mg/kg Q2W	IV infusion	NIMP	Provided locally by the study site.

6.1 Investigational product: XB2001

6.1.1 Active ingredient, pharmacologic class, structure

The XB2001 antibody is depicted below.

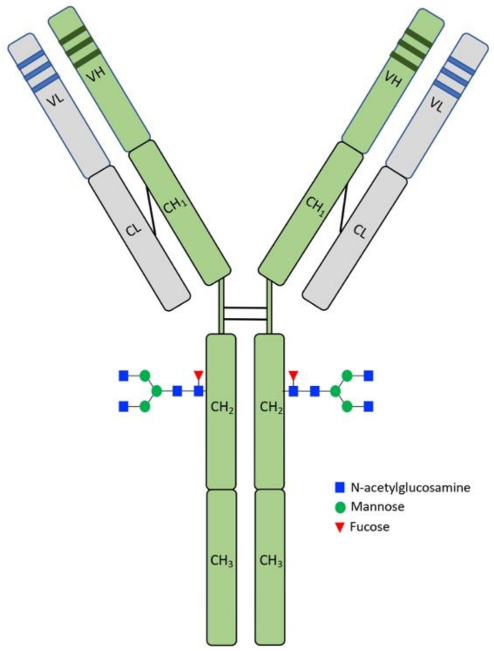

XB2001 has an IgG4, heavy chain and a kappa light chain. The heavy chains are joined at their hinge regions through two disulfide linkages and each heavy chain is joined to one light chain through one disulfide linkage between their CH1 and CL domains respectively. The heavy chain CH2 domains are glycosylated primarily with the oligosaccharide structure shown in below, as determined by cleaved Nglycan analysis performed by ProteomeLab PA800 Plus. The glycosylated residue (Asn-295 as numbered from the N-terminus of XB2001) is in the same highly conserved N-linked glycosylation site as found in endogenous IgG4 (Asn-297 according to the generic numbering system). Similarly, the primary glycan, commonly referred to as G0F, is the same as that found in about 22% of endogenous human IgG molecules[37].

The main isoform has a pI of about 6.47 and comprises over 60% of the total isoform population, as determined by capillary isoelectric focusing. The entire XB2001 heavy and light chain sequences are identical to those found in naturally-occurring human IgG4-k, with the light and heavy chain variable regions being identical to those originally expressed by a peripheral blood B lymphocyte that was obtained from a healthy individual.

Endogenous anti-IL-1α antibody has been reported in 5% to 28% of healthy serum or plasma samples [38,39]. It has been measured in cord blood, children and adults19. The anti-IL-1α antibodies measured in human plasma have been strictly of the IgG class, particularly IgG1, IgG2, and IgG4. Relatively equal distribution is seen in male and female plasma20. Binding affinities reported for endogenous anti-IL-1α antibodies, ranging from 4 to 16 pM, are comparable to that for XB2001, which is ~60 pM.

It is important to point out that affinity maturation had already taken place in the human host, and therefore no in vitro affinity maturation was required to increase the natural binding affinity of XB2001. In addition, the IgG4 heavy chain and kappa light chain constant regions have not been modified in any way in vitro.

These two features should make for a drug product with reduced potential for immunogenicity.

6.1.2 Drug product (XB2001 100mg/mL) description

XB2001 100mg/ml

The drug products include sterile liquid formulations of 100 mg/mL of XB2001 in a stabilizing isotonic formulation buffer of pH at ~ 7.0. The 100 mg/mL drug product is packaged in glass vials for the primary package. The vials used are 10-mL Type I borosilicate clear glass serum vial containing 10mL of the formulation and are sealed with a 20-mm Daikyo Flurotec butyl rubber stopper and flip-off aluminum seal.

6.1.3 Storage

The recommended storage condition for drug product in vials is upright, at 2-8°C, protected from light.

6.1.4 Stability

The drug product is formulated in a buffer in which most of the tonicity comes from trehalose rather than salt. Trehalose is an effective stabilizer against oxidation and aggregation, as well as thermal and mechanical stress.

6.1.5 Dosing and methods of administration

Treatments will continue until disease progression, unacceptable toxicity or study completion. For safety information on all treatments please see section 7.2.

XB2001 will be administered at 250 mg, 500 mg or 1000 mg dose levels by intravenous infusion for phase I portion.

XB2001/placebo will be administered at 1000 mg doses by intravenous infusion at each cycle (14 days) for phase II portion.

Partial or one vial of XB2001/placebo (10 mL at 100 mg/ml) should be diluted in normal saline prior to infusion as instructed in the pharmacy manual.

The drug product should then be mixed by gently inverting the bag ten times. After priming the infusion set lines, the delivery pump should be programmed to deliver 100 mL of the diluted drug/placebo product over up to a 1-hour period (60 +/- 15 minutes). The infusion should be chased with a minimum of 30mL of normal saline to deliver any product that may be held up in the dead volume of the infusion set.

After the first infusion, patient will stay 30 minutes in observation after the end of the perfusion.

6.1.6 Justification for 1000 mg dose selection

The present mAb is a human-derived anti-IL-1α antibody. About 20% of the healthy human population have high affinity, neutralizing anti-IL-1α antibodies circulating in the blood. XBiotech has been developing human-derived antibodies, including anti-IL-1α antibodies, in the clinic over the past 10 years. This extensive clinical experience has proven safety against both the target (IL-1α) and the use of true human derived antibodies (ie, absence of human anti-human antibody responses).

XBiotech has used another human-derived anti-IL-1α antibody (MAbp1) in both intravenous and subcutaneous delivered formulation. Intravenous delivery was explored in a dose escalation study performed in refractory cancer patients with no maximum tolerated dose (MTD) achieved, since no significant toxicity was observed to limit dose escalation, with the highest dose tested deemed to be the minimum effective dose[40]. Over 2,000 doses of the human derived anti-IL-1α antibody were administered at 7.5 mg/kg in cancer patients with cancer associated symptoms at baseline (ECOG performance status 1 and 2) without any definitive related toxicity[41].

The previous anti-IL-1α antibody was also used in a clinical study in a chemotherapy combination—nanoliposomal irinotecan (Nal-Iri) and 5-fluorouracil (5FU)/folinic acid (FA)—and these data were recently presented[42]. The study evaluated 18 patients treated for advanced pancreatic adenocarcinoma and cachexia who had previously failed gemcitabine-based chemotherapy (NCT03207724). The antibody was not only well-tolerated at all dose levels including the maximum dose level of 12mg/kg, but appeared to reduce toxicities normally associated with the cytotoxic therapy combination (12mg/kg is equivalent to 924mg dose in typical patients weighing an average of 77 kg).

XB2001 was isolated from the B-cells of a healthy human donor after affinity maturation had already taken place in vivo and no additional in vitro affinity maturation was performed in vitro to increase the naturally high binding affinity of this antibody. In addition, the Fc region of the XB2001 is an unaltered IgG4 heavy chain constant region naturally present in humans. Therefore, XB2001 should have minimal potential for immunogenicity.

Due to the disproportionate increase in blood volume with respect to increase in body weight, current knowledge of elimination of monoclonal antibodies, and the publicly available data from clinical trials and extensive population pharmacokinetic (PopPK) modeling justifies that the body size-based dosing for monoclonal antibody therapy in oncology is not supported and fixed dosing has been suggested to be more appropriated. Currently most immunotherapy based mAb are given using fixed doses. Therefore, supported by the strong track record of lack of immunogenicity and toxicity for monoclonal antibodies developed by XBiotech's proprietary platform, and the immediate need for available treatment for life-threatening colorectal cancer, a fixed dose is proposed in this study.

Human pharmacokinetic data for XB2001 are currently available from an ongoing phase I/II cancer study 1-BETTER (NCT04825288), where XB2001 is used in combination with ONIVYDE + 5-FU/LV (+folinic acid). Observed Cmax and Day 14 plasma levels for XB2001 are presented in Figure 1

Figure 1 XB2001 Plasma Concentration Box Plot from 250 mg Cohort of Pancreatic Cancer Trial (NCT04825288)

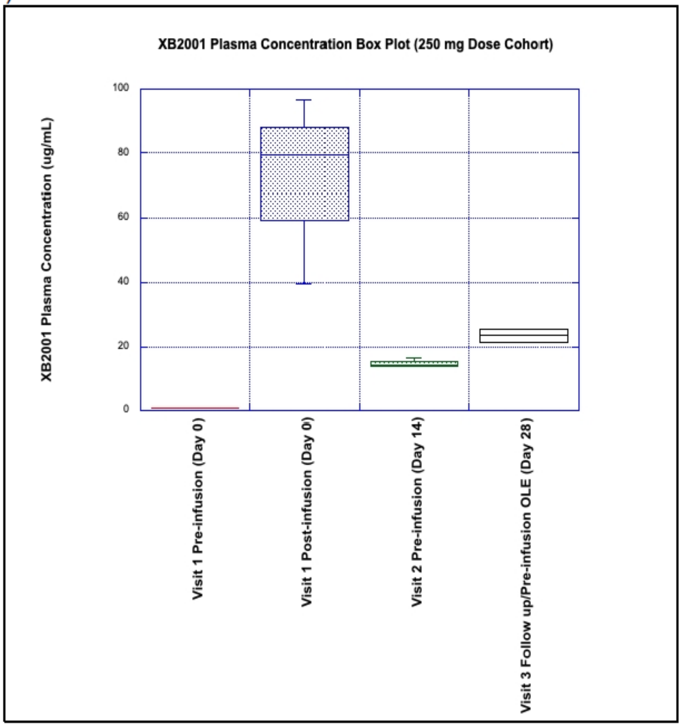

The average Cmax is consistent with previous clinical pK data for MABp1, where XBiotech demonstrate that concentration of antibody is proportional to the dose levels. The 1-BETTER Cmax of XB2001 at 250 mg (equivalent to ~ 3.3 mg/kg using average adult weight of 75 kg) is just as expected if using the MABp1 Cmax-dose relationship curve (Figure 2) and consistent with animal pk data (see Figure 3).

Figure 2

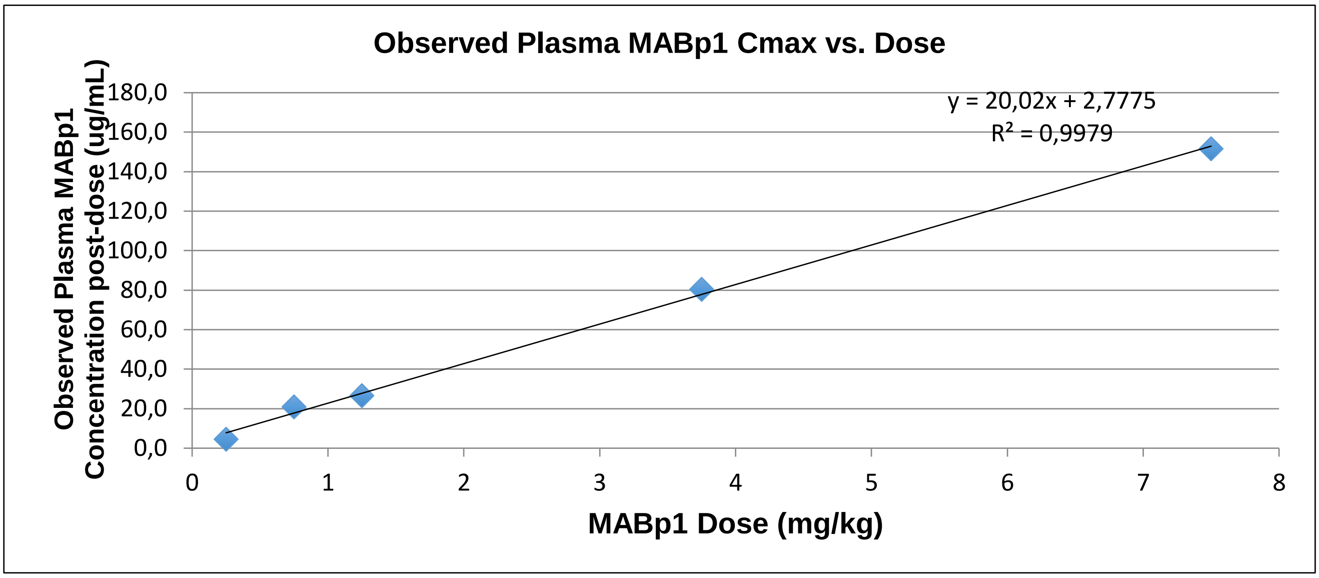

Figure 3:

Figure 2 XB2001 Animal PK Curve with One Single Dose of 1 mg (~ 40 mg/kg)

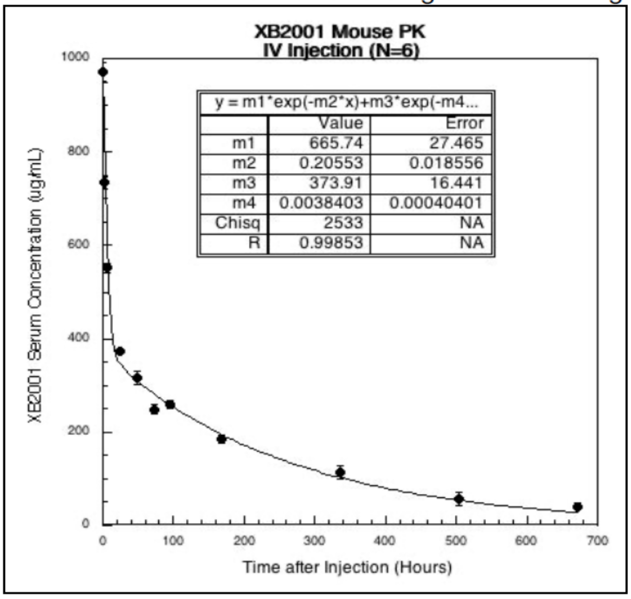

In the mice PK study, 1.0 mg was intravenously injected and an average Cmax (N=6) of 1040 µg/mL was observed. The 1.0 mg dose of monoclonal antibody (in a 20 g mouse) is equivalent to a dose of 3.75 g in human (75 kg weight), which is about 15-fold higher than the 250 mg initial dose proposed in the study. The estimated serum Cmax for 250 mg, based on animal PK data, is 70 µg/mL. This is consistent with an expected Cmax of about 80 µg/mL, based solely on estimated human plasma volume at 250 mg. The concentration seen at Day 14 is as expected, based on comparison to the day 14 concentration previously observed for a similar anti-IL-1a antibody, and according to the pK clearance curve of XB2001 seen in murine model (N=6). The mice PK curve of XB2001 suggested that concentration at Day 14 is 11% of the Cmax. So the expected Day 14 concentration for 250 mg is expected to be 10 µg/mL, which is consistent with the observations (Figure 1).

The targeted minimum plasma concentration for XB2001 is ~ 60 µg/ml. The target concentration is based on previous clinical experience with MABp1 (Hendifar, A. E., Kim, S., et. al. (2020). A phase I study of nanoliposomal irinotecan and 5-fluorouracil/folinic acid in combination with interleukin-1-alpha antagonist for advanced pancreatic[46] cancer patients with cachexia (OnFX)). Based on the human (and animal) pharmacokinetic observations and a treatment cycle of two weeks, an XB2001 dose level of 1,000 mg has been proposed to reach the target plasma concentration. Based on the proposed dose, a Cmax of approximately 350 µg/ml and a minimum serum concentration of ~ 59 µg/ml are assumed to be observed in subjects.

To be confident in dose determination, dose retain for phase II will be based on data obtained from MTD, but also from PK/PD. The dose level retain for phase II will be based on the DLT as classical for phase I and will also be confirmed based on pharmacokinetic study to validate the expected Cmax and absence of evidence of accumulation or increased clearance of XB2001 at increasing doses. Additionally, PK data will be correlated to biological activity, by testing the decrease of IL-6 as previously reported[45]. The IDMC will validate the dose retain for phase II based on PK/PD and MTD

To assess immunogenicity, anti-drug antibody responses are measured using a proprietary sandwich ELISA developed by XBiotech. Patient plasmas areincubated on XB2001-coated microplates followed by labelled secondary antibodies. Measurements are taken at the same timepoints as for pharmacokinetic data.

6.2 Non-investigational product: trifluridine/tipiracil - Bevacizumab

Standard of care should be at the discretion of each investigator with:

- Trifluridine/tipiracil (Lonsurf®) alone
- Trifluridine/tipiracil (Lonsurf®) + Bevacizumab

The TASKIN study does not impose a standare of care. The use of bevacizumab is at the discretion of each investigator according to local practice.

Trifluridine/tipiracil (Lonsurf®) and **bevacizumab** treatment **<u>will not be provided by the sponsor</u>**. Trifluridine/tipiracil and bevacizumab will be used in accordance with the marketing authorization indication in adults with metastatic colorectal cancer (mCRC):

The data provided in the SPC for the trifluridine/tipiracil and bevacizumab products should be followed. The information below are provided for information only and does not replace the guidelines and recommendations.

- **<u>Trifluridine/tipiracil:</u>**

The recommended dose is 35 mg/m^2/dose administered orally, twice daily, on days 1 to 5 and then on days 8 to 12 of each treatment cycle (1 treatment cycle = 28 days). Treatment should be continued for as long as therapeutic benefit is observed or until the development of unacceptable toxicity.

The dose to be taken in each administration should be calculated based on the body surface area (SC) (see Table below). The dose should not exceed 80 mg / dose. The patient should not make up for missed doses.

- **Calculation of initial dose based on body surface area (BSA)**

Dose after reduction	Body surface	Dose of trifluridine/tipiracil per administration in mg (twice a day)	Number of tablets per administration (twice a day)		Total daily dose (mg)
			15mg/6.14 mg	20mg/8.19mg	
35mg/m²	< 1,07	35	1	1	70
	1,07 - 1,22	40	0	2	80
	1,23 - 1,37	45	3	0	90
	1,38 - 1,52	50	2	1	100
	1,53 - 1,68	55	1	2	110
	1,69 - 1,83	60	0	3	120
	1,84 – 1,98	65	3	1	130
	1,99 - 2,14	70	2	2	140
	2,15 – 2,29	75	1	3	150
	≥ 2,30	80	0	4	160

- **Bevacizumab:**

Bevacizumab is a humanised monoclonal antibody which is a type of protein normally made by the immune system to help the body defend itself against infections and cancers.

Bevacizumab selectively binds to a protein called human vascular endothelial growth factor (VEGF) which is present in the lining of blood and lymph vessels. The VEGF protein induces the growth of blood vessels in tumors. These vessels bring the oxygen and nutrients needed by tumors. Once bevacizumab binds to VEGF, tumor growth is inhibited by blocking the formation of new vessels that supply the tumor with nutrients and oxygen.

Avastin is a medicine used in adult patients for the treatment of metastatic cancers of the large intestine, for example the colon or rectum. Avastin will be given in combination with chemotherapy containing a fluoropyrimidine.

Bevacizumab is recommended at a dose of 5 mg/kg of body weight given once every 2 weeks by intravenous infusion. It is recommended that treatment be continued until progression of the underlying disease or unacceptable toxicity.

Do not shake the bottle. Bevacizumab is a solution to be diluted in a solution of sodium chloride before administration by infusion. As per local guidance, the duration of the first infusion could be 90 minutes. If tolerance is good, that of the second infusion may be 60 minutes. Subsequent infusions could be administered over 30 minutes.

Bevacizumab indication is according to standard practice of each investigator site. Standard Arm should be the standard of care of the site in the indication of metastatic colorectal cancer treatment.

6.3 Dose adaptation

6.3.1 General guidance

The following general guidance should be followed for management of toxicities

All toxicities should be treated with maximum supportive care (including withholding the suspected agent causing the toxicity if required).

If the symptoms are promptly resolved with supportive care, continuing the same dose of the assigned investigational product with appropriate continuing supportive care should be considered.

Dose interruptions of trifluridine/tipiracil +/- bevacizumab does not cause a stop of the administration of XB2001. If trifluridine/tipiracil +/- bevacizumab administration is stopped / suspended, administration XB2001 could be maintained. If administration of trifluridine/tipiracil +/- bevacizumab is permanently discontinued, administration of XB2001 can be continued until progression or change of treatment.

All dose modification of trifluridine/tipiracil +/- bevacizumab or XB2001 interuption should be documented with clear reasoning and documentation of the approach taken.

Subjects who are experiencing unacceptable toxicities should discontinue treatment and be removed from study (see section 4.4.4).

In case of any doubt for the investigators for toxicity management of XB2001, the advice of the sponsor and the coordinator could be requested.

6.3.2 XB2001 dose adaptation

XB2001 dose will not be adapted. Dose will be administered or not but no dose reduction would be performed.

- **Toxicity from the association XB2001 + trifluridine/tipiracil +/- bevacizumab or XB2001 alone RELATED OR PROBABLY RELATED TO XB2001**

- Grade 1 or 2:
No interruption of XB2001 required or at the discretion of the investigator.

- Grade 3: XB2001 will be stopped until the toxicity is resolved or return at a grade ≤ 1 (or baseline grade) and restart without dose modification. If the event recurs second time, the patient will discontinue the treatment again and restart without dose modification when toxicity is resolved or return at a grade ≤ 1 (or baseline grade). If the event recurs à 3rd time, XB2001 will be discontinue permanently. Unless it is an hematological toxicity, XB2001 will be stopped at the 2nd recurrence of the hematological event.

- Grade 4: XB2001 will be stopped until the toxicity is resolved and restart without dose modification. If the event recurs XB2001 will be discontinue permanently.

- **Toxicity from the association XB2001 + trifluridine/tipiracil +/- bevacizumab or XB2001 alone NOT RELATED TO XB2001**

- Grade 1 or 2: No interruption of XB2001 required or at the discretion of the investigator.

- Grade 3 or 4: XB2001 could be stopped until the toxic problem is resolved or return at a grade ≤ 1 (or baseline grade) at investigator discretion and then restart without dose modification.

- **Severe infection**

In case of severe infection, XB2001 should be immediately suspended until the infection is resolved.

- **Proteinuria**

A urine dipstick is performed at each cycle. If 2 and 3 crosses or value > 1g/h treatment will be stopped until return to a baseline result or grade ≤ 1.

- **Infusion-Related Reactions**

**Infusion-Related Reactions**		
**Severity Grade of the Event (NCI CTCAE version 5.0)**	**Dose Modifications**	**Toxicity Management**
**Any Grade**	General Guidance	**For Any Grade:**<br>– Manage per institutional standard at the discretion of investigator.<br>– Monitor patients for signs and symptoms of infusion-related reactions (e.g., fever and/or shaking chills, flushing and/or itching,

Infusion-Related Reactions		
**Severity Grade of the Event (NCI CTCAE version 5.0)**	**Dose Modifications**	**Toxicity Management**
		alterations in heart rate and blood pressure, dyspnea or chest discomfort, or skin rashes) and anaphylaxis (e.g., generalized urticaria, angioedema, wheezing, hypotension, or tachycardia).
**Grade 1 or 2**	**For Grade 1:**<br>The infusion rate of study drug/study regimen may be decreased by 50% or temporarily interrupted until resolution of the event.<br>**For Grade 2:**<br>The infusion rate of study drug/study regimen may be decreased 50% or temporarily interrupted until resolution of the event.<br>Subsequent infusions may be given at 50% of the initial infusion rate.	**For Grade 1 or 2:**<br>– Acetaminophen and/or antihistamines may be administered per institutional standard at the discretion of the investigator.<br>– Consider premedication per institutional standard prior to subsequent doses.<br>– Steroids should not be used for routine premedication of Grade ≤2 infusion reactions.
**Grade 3 or 4**	**For Grade 3 or 4:**<br>Permanently discontinue study drug/study regimen.	**For Grade 3 or 4:**<br>– Manage severe infusion-related reactions per institutional standards (e.g., IM epinephrine, followed by IV diphenhydramine and ranitidine, and IV glucocorticoid).

6.3.3 Trifluridine/tipiracil +/- bevacizumab dose adaptation

Trifluridine/tipiracil +/- bevacizumab should be adjusted regarding standard practice. As an indication, the information below is presented but does not replace the information regarding trifluridine/tipiracil +/- bevacizumab dose adjustments present in the summary of product characteristics via the link below

https://ec.europa.eu/health/documents/community-register/2020/20200428147918/anx_147918_fr.pdf or http://base-donnees-publique.medicaments.gouv.fr/extrait.php?specid=65185956#

Dosage adjustments may be necessary depending on individual safety and tolerability of the patient.

➢ <u>**Trifluridine/tipiracil**</u>

A **maximum of 3 dose reductions is allowed**, without going below the minimum dose of 20 mg / m^2 twice a day. **The dose should not then be increased again if it had previously been reduced**.

In the event of haematological and / or non-haematological toxicities, patients must comply with the criteria interruption and resumption of treatment, as well as the methods of reducing the doses administered, mentioned below:

- **Haematological toxicity linked to myelosuppression**, criteria for interrupting and resuming treatment

Parameter	Interrupt criterion	Restart criterion(a)
Neutrophils	< 0,5 X 109/L	≥ 1,5 X 109/L
Platelet	< 0,5 X 109/L	≥ 75 X 109/L

(a) Criteria for restart treatment, applied for the initiation of the next treatment cycle, for all patients, regardless of whether the discontinuation criteria have been met.

- **Haematological or non-haematological adverse reactions** : Dosage adjustments recommended

**Adverse reaction**	**Recommended dosage adjustement**
Febrile neutropenia	- Interrupt the administration of treatment until the toxicity is resolved to a grade 1 or at baseline level noted before treatment<br>- When resuming treatment, reduce the dose per administration of 5 mg / m^2 relative to previous dosage<br>- The dose can be reduced by administration up to a minimum of 20 mg / m^2, twice daily (or 15 mg/m^2, twice per day in severe renal impairment)<br>- **Do not re-increase the dose when it has previously been reduced**
Grade 4 neutropenia (<0.5×10^9/L) requiring to delay the initiation of the cycle next more than 1 week	
Grade 4 thrombocytopenia (<25×10^9/L) requiring to delay the initiation of the cycle next more than 1 week	
Grade 3 or 4 non-haematological adverse reaction except for grade 3 nausea and / or vomiting controlled by a antiemetic therapy or diarrhea controlled by an anti-diarrheal medication	

- **Calculation of dose per administration based on body surface area (BSA) during decreases in dose**

**Dose after reduction**	**Body surface**	**Dose of trifluridine per administration in mg (twice a day)**	**Number of tablets per administration (twice a day)**		**Total daily dose (mg)**
			15mg/6,14 mg	20mg/8,19mg	
	**Level 1 dose reduction**: reduction from 35 mg/m^2 to 30 mg/m^2 per administration				
30mg/m^2	< 1,09	30	2	0	60
	1,09 - 1,24	35	1	1	70
	1,25 - 1,39	40	0	2	80
	1,40 - 1,54	45	3	0	90
	1,55 - 1,69	50	2	1	100
	1,70 - 1,94	55	1	2	110
	1,95 - 2,09	60	0	3	120
	2,10 - 2,28	65	3	1	130
	≥ 2,29	70	2	2	140
	**Level 2 dose reduction**: reduction from 30 mg/m^2 to 25 mg/m^2 per administration				
25mg/m^2	< 1,10	25[(a)]	2[(a)]	1[(a)]	50[(a)]
	1,10 - 1,29	30	2	0	60
	1,30 - 1,49	35	1	1	70
	1,50 - 1,69	40	0	2	80
	1,70 - 1,89	45	3	0	90
	1,90 - 2.09	50	2	1	100
	2,10 - 2,29	55	1	2	110
	≥ 2,3	60	0	3	120
	**Level 3 dose reduction**: reduction from 25 mg/m^2 to 20 mg/m^2 per administration				
20mg/m^2	< 1,14	20	0	1	40

	1,14 – 1,34	25[(a)]	2[(a)]	1[(a)]	50[(a)]
	1,35 – 1,59	30	2	0	60
	1,60 – 1,94	35	1	1	70
	1,95 – 2,09	40	0	2	80
	2,10 – 2,34	45	3	0	90
	≥ 2,35	50	2	1	100

[(a)] For a total daily dose of 50 mg, patients should take 1 tablet of trifluridine/tipiracil 20 mg / 8.19 mg in the morning and 2 tablets of trifluridine/tipiracil 15 mg / 6.14 mg in the evening.

- **Dosage adjustments for patient with renal failure**

Please refer to the summary of product characteristics for specific instructions regarding this population.

6.4 Assessments of trifluridine/tipiracil compliance

The trifluridine/tipiracil will be dispensed by city pharmacist.

A patient diary will be provided to assess complicance with trifluridine/tipiracil. It will contain detailed description of the treatment :

- The name of the investigator, the contact number of the key person to be contacted in case of problem,
- The modalities of storage of trifluridine/tipiracil
- The recommendations about capsules of trifluridine/tipiracil intake
- Pages per week of treatment intake in which the patient will enter key information about it (date and hour of intake, number of capsules taken)
- Diary should be brought back at each appointment, and will be collected by the investigator or delegated site staff and included in the study file at the end of the study.

eCRF data will provide 2 types of informations regarding compliance:
- Diary compliance
- Real compliance after compatibility performed at the pharmacy

If any discrepancies exist and in particular if the real compliance is less than 80%, the investigator in charge of the patient should be notified.

6.5 Shipment, storage, accountability and destruction

6.5.1 XB2001 / Placebo

XB2001/Placebo will be provided by the sponsor and will be distributed to the pharmacy at the investigational site via a dedicated central pharmacy (Eurofins-LC2) subcontracted by the sponsor. The pharmacy staff will be open label for the study and identification of XB2001 or placebo will be provided on the bottle.

The pharmacist of each site will receive numbered treatment and will need to acknowledge each receipt of all shipments to the central pharmacy Eurofins-LC2.

The pharmacist is responsible for a safe and proper handling and storage of the investigational product at the investigational site. The investigational product must be stored in locked facility with restricted access to the pharmacist and authorised personnel, and under environmental conditions consistent with the drug manufacturer recommendations.

XB2001/Placebo is Stored in the refrigerator (+2 ° C to +8 ° C), should not be frozen and should be stored in the original package in order to protect from light.

In case of temperature excursions outside the labelled storage conditions, sponsor should be immediatly informed to provide guidelines for storage. Temperature log should be kept updated by the pharmacist, to document adequate storage during the study.

The investigator/pharmacist must ensure that the investigational product is administrated only to patient enrolled in this study and in the experimental arm. The investigational product must not be used outside the context of the study protocol.

The pharmacist or the authorised staff must document the receipt, the dispensation, the return and the destruction of all investigational products received during the study. Records should mentionned delivery to the site, the inventory at the site, the use by each patient and the destruction by the site must be implemented and maintained by the pharmacist or anothe appropriately trained and delegated person at the investigational site. Will be reported on these records at least the dates, quantities, batchnumber of the investigational products. Forms will be provided by the sponsor to ensure trial treatment accountability.

The pharmacist will implement and accounting of medicinal products dispensed, used, unused. Dispensation traceability will cover the use of XB2001.

This process will be monitored by the sponsor CRA during the study and the pharmacy monitoring visit; the CRA will check that the accountability documentation has been filled in and signed by the pharmacist before the used and that unused investigational product are destroyed.

The destruction of all remaining investigational products, used and unused, will take place at the investigator's sites under the responsability of the pharmacist in accordance with national regulatory requirements, and with prior formal agreement from the sponsor. A certificate of destruction, identifying the concerned products will be given to the sponsor.

6.5.2 Trifluridine/tipiracil +/- bevacizumab

Trifluridine/tipiracil +/- bevacizumab will not be provided by the sponsor. The handling, storage and delivery will be performed according to standard practice and local regulation of each pharmacy.

For the study it will be asked to the patient to bring back his boxes during the on-site visit in order to collect the batch number and the expiration date.

6.6 Restriction during the study

6.6.1 Birth control measures

It is not known that trifluridine/tipiracil can reduced the effectiveness of hormonal contraceptive treatments. As a result, **patients using hormonal contraception must also use a mechanical contraceptive method.**

✓ <u>Definitions of females of chilbearing potential and post-menopausal status :</u>

- Females of childbearing potential are defined as those who are not surgically sterile (i.e bilateral tubal ligation, bilateral oophorectomy or complete hysterectomy) or post-menopausal

- Post-menopausal status is defined as: women with amenorrheic for 12 months without an alternative medical cause. The following age-specific requirement apply:

- Women < 50 years old would be considered post-menopausal if they have been amenorrheic for 12 months or more following cessation of exogenous hormonal treatments and if they have luteinizing hormone or follicle-stimulating hormon levels in the post-menopausal range for the institution or underwent surgical sterilization (bilateral oophorectomy or hysterectomy)
- Women ≥ 50 years old would be considered post-menopausal if they have been amenorrheic for 12 months or more following cessation of exogenous hormonal treatments, had radiation-induced menopause with last menses > 1 year ago, had chemotherapy-induced menopause with last menses > 1 year ago, or underwent surgical sterilization (bilateral oophorectomy, bilateral salpingectomy or hysterectomy).

✓ <u>Female patients of chilbearing potential:</u>

Females of childbearing potential who are sexually active with a non-serilized male partner must use at least 1 highly effective method of contraception from the time of screening and must agree to continue using such precautions for 30 days after the last dose of trifluridine/tipiracil +/- XB2001. Non-sterilized male partners of a female patient must use male condom plus spermicide throughout this period. Cessation of birth control after this point should be discussed with the responsible physician. Not engaging in sexual activity for the total duration of the study treatment and the drug washout period is an acceptable practice; however, periodic abstinence, the

rhythm method, and the withdrawal method are not acceptable methods of birth control. Female patients should also refrain from breastfeeding throughout this period.

- ✓ Male patients with a female partner of chilbearing potential :

Non-sterilized males who are sexually active with a female partner of childbearing potential must use a male condom plus spermicide from screening through **6 months** after the last dose of trifluridine/tipiracil. Not engaging in sexual activity is an acceptable practice; however, the rhythm method and the withdrawal method are not acceptable methods of contraception. Male patients should refrain from sperm donation throughout this period.

Female parteners (of childbearing potential) of male patients must also use a highly effective method of contraception throughout this period.

- ✓ Contraception methods:

Highly effective methods of contraception are defined as those that result in a low failure rate (i.e, less than 1% per year) when used consistently and correctly.

6.6.2 Blood donation

Patient should not donate blood while participating in this study for at least 90 days following the last administration of investigational product.

6.7 Concomitant treatments

All medications (including herbal preparations) and therapies taken by the patients or administered to the patient's onset of the study and given in addition to the investigational products during the study are considered as concomitant medications. Any concomitant medication(s) taken during the study will be recorded in the eCRF.

There are no known drug interactions with XB2001.

6.7.1 Authorised concomitant treatments

Cytochrom P450: When performing in vitro studies, trifluridine/tipiracil hydrochloride and 5- [trifluoromethyl] uracil (FTY) did not inhibit the activity of human cytochrome P450 (CYP) isoforms. An evaluation in vitro indicates that trifluridine/tipiracil hydrochloride and FTY had no inducing effect on the human CYP isoforms

The prescription of anti-emetics and G-CSF remains at the discretion of the investigators.

Subjects are permitted to use insulin or other anti-diabetics to control blood sugar, any recommended analgesics, medications to reduce/control the side effects of chemotherapy and anti-infectives/antibiotics.

6.7.2 Prohibited concomitant treatments

- O**ther anti-cancer treatments** than trifluridine/tipiracil are not allowed during the study

- Prescriptions of **immunosuppressive** or **corticosteroid** therapy longer than 5 days or greater than or equal to 1 mg/kg are not authorized. Immunotherapies/biologics, including agents that inhibit tumor necrosis factor (TNF), interleukin 1 (IL-1), epidermal growth factor (EGFR) and Programmed cell death protein 1 (PD-1) are prohibited.

- **Live virus vaccines** should not be given during treatment with XB2001.

- Herbal medicine

In the event that it becomes medically necessary for a subject to be treated with a restricted therapy during the study, this should be recorded both in the source document and in the eCRF as a concomitant therapy, however the subject may continue trial as scheduled if PI and sponsor are in agreement.

6.7.3 Concomitant treatments use with caution

- Concomitant treatment with **anti-TNF-α** (ex etanercept or other) is not recommended

- In vitro studies have shown that trifluridine is a substrate vis-à-vis the **nucleoside transporters CNT1, ENT1 and ENT2**. Therefore, caution is required when using drugs that interact with these carriers. As tipiracil hydrochloride is a substrate of the OCT2 and MATE1 transporters, the concentration plasma levels may therefore be increased if trifluridine/tipiracil were administered in combination with **inhibitors of OCT2 or MATE1**.

- Caution is required when using drugs which are **substrates for thymidine kinase human**, such as zidovudine. These drugs, if used concomitantly with trifluridine/tipiracil, may compete with trifluridine in their activation reaction by phosphorylation via thymidine kinase. Therefore, when concomitantly using **antiviral drugs that are substrates of human thymidine kinase**, a possible decrease in the effectiveness of the antiviral agent should be monitored and consider an antiviral therapeutic alternative using drugs that are not substrates of human thymidine kinase, such as lamivudine, didanosine and abacavir.

7. EVALUATION OF TREATMENT EFFICACY AND SAFETY

7.1 Efficacy evaluation

Efficacy will be evaluated using overall survival (OS), objective response rate and progression free survival (PFS).

✓ **Overall survival** (OS) is defined as the time interval from randomization to the date of death from any cause. Patients alive will be censored at the last date of last known follow-up.

✓ **Objective response rate** will be evaluated by Tumor evaluations/assessments and will be performed every 8 weeks from the initiation of treatment for the first 12 months of treatment or until first disease progression (whichever comes first), and then every 12 weeks until disease progression, when applicable.

The tumor evaluation dates for tumor evaluations (by RECIST) are not modified by dose delays. The assessments dates are relative to the treatment initiation date.

Treatment efficacy will be evaluated by measuring changes in tumor size in response to treatment mesured by computed tomography (CT) scan or magnetic resonance imaging (MRI) of the chest, abdomen and pelvis.

- For the delay of treatment management and the principal objective assesment, the treatment response will be evaluated by the investigator using RECIST v.1.1.

- Blinded indemendent central review committee (BICR):

An independant radiological review will be scheduled at the end of the study. The BICR will review centraly all the radiological tumor assessments according to RECIST v 1.1 for all the patient treated with at least one dose of treatment. Investigator of each site will be asked to upload DICOM format record of the tumor assessment to the web platform subcontracted by the sponsor; for each baseline and subsequent radiological tumor assesment for all enrolled patients.

✓ **Progression free survival** (PFS) is defined as the time interval from randomization to the date of progression (defined using RECIST 1.1 criteria) or death from any cause. Patients alive without progression will be censored at the last date of last known follow-up. Median PFS as well as PFS rates at 2 and 4 months will be provided.

7.2 Safety evaluation

Treatment safety evaluation will be based on adverse event (AE) occurrence, the use of concomitant treatments, changes occuring in the course of treatment, observed during physical examination, in the vital signs (arterial pressure, pulse and body temperature), in electrocardiogram (ECG) and biological and clinical examinations (biochemistry, haematology). Safety will be assessed by NCI-CTCAE V5.0.

All safety scheduled assessments must be performed relative to the start of the dosing cycle so that all laboratory procedures required for dosing should be performed within 3 days prior to dosing.

In case of emergency, the patient, a patient’s relative or the patient’s general practitioner will inform the investigator, by phone of the occurrence of an AE.

It is anticipated anticipated that arround 15-20%of patient treated with trifluridine/tipiracil +/- bevacizumab will develop severe toxicities. An increase in toxicities is not expected for patients who will benefit from an addition of XB2001 to treatment with trifluridine/tipiracil +/- bevacizumab + placebo.

7.2.1 Physical examination

Physical examinations will be performed according to the assessment schedule. Full physical examinations include assessments of the head, eyes, ears, nose, and throat and the respiratory, cardiovascular, gastrointestinal, urogenital, musculoskeletal, neurological, dermatological, hematologic/lymphatic, and endocrine systems. Height will be measured at baseline only. Targeted physical examinations are to be utilized by the Investigator on the basis of clinical observations and symptomatology. Modification observed on physical examination results should be reported as AE in the eCRF if clinically significant according to investigator judgement.

7.2.2 Electrocardiograms

Resting 12-lead ECGs will be recorded at screening, as clinicaly indicated during the treatment and at the end of treatment visit. ECGs should be obtained after the patient has been in a supine position for 5 minutes and recorded while the patient remains in that position.

7.2.3 Vital signs and weight

Vital signs: blood pressure (BP), pulse, temperature, and respiration rate will be evaluated according to the assessment schedules. Body weight is also recorded at each visit if possible alone. Any clinically significant changes in vital signs should be entered in eCRF as an Adverse Event.

7.2.4 Clinical laboratory tests

Blood and urine samples for determination of clinical chemistry, hematology, and urine analysis will be taken at the times indicated in the assessment schedules and as clinically indicated

Clinical laboratory safety tests, including serum pregnancy tests, will be performed in the hospital's laboratory according to local standard procedures. Sample tubes and sample sizes may vary depending on the laboratory method used and routine practice at the site. Urine pregnancy tests may be performed at the site using a licensed test (urine or serum pregnancy test). Abnormal clinically significant laboratory results should be repeated as soon as possible (preferably within 24 to 48 hours).

Additional safety samples may be collected if clinically indicated at the discretion of the Investigator. The date, time of collection, and results (values, units, and reference ranges) will be recorded on the appropriate eCRF.

Other safety tests to be performed at screening include assessment for hepatitis B surface antigen, hepatitis C antibodies and HIV antibodies.

Test category	Test name
**Haematology**	Absolute Neutrophil count (ANC), absolute lymphocyte count, haemoglobin, platelet count
**Clinical chemistry**	Albumin, alkaline phosphatase, ASAT, ALAT, calcium, chloride, creatinine clearance, GGT, glucose, LDH, magnesium, potassium, sodium, total bilirubin, total protein.
**Tumor markers**	ACE
**Urinalysis**	Bilirubin, blood, colour and appearance, glucose, ketones, pH, **protein**, specific gravity.

8. DESCRIPTION OF VISITS AND INVESTIGATIONS

The flow chart summarising the follow-up examination/visit schedule from portion of phase I is provided in table 1 in the begining of the protocol. The flow chart summarising the follow-up examination/visit schedule from portion of phase II is provided in table 2 in the begining of the protocol. A general +/-3 days window is allowed for assessments or visit performed every 4 weeks. As well a +/-3 days window is allowed for the 2nd injection of

XB2001 performed during each cycle; 1 Cycle = 28 days. Except for visit C1D12 with no visit window allowed due to PK sampling. A +/-7 days window is allowed for tumor evaluations.

8.1 Baseline/screening assessment for phase I or phase II of the study

Informed consent form (ICF) must be signed before the evaluations required for inclusion in phase I and inclusion/randomization of phase II and any study specific procedure are performed.
After signing the study ICF, baseline assessments will be done within 1 to 21 days prior to inclusion of phase I or randomization of phase II depending on assessments.
A procedure or evaluation already performed within the standard of care and which is in accordance with the protocol requirements does not need to be repeated unless clinically justified.
For laboratory evaluations used to determine eligibility, a repeated evaluation within the screening window is permitted for baseline results out of the defined range.

8.1.1 Eligibility screening for baseline visit phase I or phase II

- Check that all baseline inclusion / non-inclusion criteria are met
- Check that the patient will adhere to contraception conditions (refer to section 6.6.1)
- Review of baseline laboratory results: any result outside the normal range may be repeated (prior the first dose) at the discretion of the investigator
- Concomitant treatment: Check the administration of prohibited therapies and consider substituting them, when possible

8.1.2 Demographics, medical and disease history for baseline visit phase I or phase II

- Demographic data
- History of the disease: clinical characteritics, pathological diagnosis with TNM classification and pTNM if applicable and previous treatement (chemotherapy, surgery…)
- Previous treatment received for the disease. Record all compound received, schedule and number of cycles
- Relevant medical and surgical history other then studied disease
- Adverse event review from residual toxicities or ongoing events
- Concomitant treatments: Record all medication(s) received within 2 weeks prior to inclusion that may interact with one of the study drugs or may induce or potentiate some drug-related adverse events and indicate all ongoing treatment.

8.1.3 Clinical examination for baseline visit phase I or phase II

- Complete full physical examination
- ECOG Performance Status
- Vitals signs (blood pressure, pulse, body temperature and respiratory rate)
- Weight
- Height
- Signs and symptoms of the disease

8.1.4 Biologicals tests for baseline visit phase I or phase II

Biological tests should be done within 14 days of inclusion/randomization

- Clinical chemistry
- Hematology
- Pregnancy tests on blood or urine samples will be performed for women of childbearing potential within 28 days prior to the start of study treatment. Tests will be performed by the hospital’s local laboratory. If results are positive the patient is ineligible/must be discontinued from study treatment immediately. Details of the pregnancy tests must be recorded in the patient’s medical records.
- Hepatitis and HIV serologies could be performed within 21 days
- Urine analysis
- ACE

Review of all baseline laboratory results: any result outside the normal range or inclusion range may be repeated (prior to the first dose) at the discretion of the investigator.

Any clinically significant abnormal laboratory values should be repeated as clinically indicated and recorded in the eCRF.

8.1.5 Paraclinical examinations for baseline visit phase I or phase II

- Tumor evaluation including the verification of the extent of disease with Imaging by CT scan, with a baseline evaluation regarding RECIST criteria 1.1. Other appropriate radiological assessment will be left to the investigator's discretion (e.g. bone scan or brain if known metastasies or clinically indicated).
- Verification of heart function by Electrocardiogram (ECG) : 12-lead ECG in triplicate in 2-5 minutes apart if clinical abnormality detected. The ECG should be obtained after the patient has been in a supine position for 5 min and recorded while the patient remains in the supine position.
- Health-related quality of life survey EORTC-QLQ-C30 and CR29

8.2 Visits and assessment during the treatment period of phase I

The study flow chart of the study presented in **table** 1 list the assessments and the visit schedule. A general +/-3 days window is allowed for assessments or visits.
All examinations revealing a toxicity related to one of the investigational products must be periodically repeated until toxicity disappearance (or until considered irreversible).
First cycle (first treatment administration) should be performed at the latest 3 days after inclusion.
The treatment phase will continue until confirmation of disease progression or discontinuation of the treatment. A visit will be scheduled on site at day 1 and day 15 of each cycle of treatment with the following exams and procedures:

8.2.1 Clinical examinations and assessments during the treatment period of phase I

- Evaluation of DLTs and toxicities according to NCI-CTCAE version 5.0
- Physical examination as clinically indicated
- ECOG performance status
- Vital signs: blood pressure, pulse, body temperature and respiratory rate. Blood pressure will be repeated during level 1, 2, 3 and 4 for all administration of XB2001 during the first months, with measurement performed prior infusion, 30-minutes after the start of the infusion at the end of the infusion and 30 minutes after the end of the infusion.
- Review and collection of concomitant treatments

8.2.2 Biological tests during the treatment period of phase I

The following biological tests must be done at each cycle

- Clinical chemistry
- Hematology
- Urine analysis
- Pregnancy test
- ACE

Blood count results and biochemistry results must be obtained within 72 hours of the planned visit. Investigator should document the review and the evaluation of those biological reports promptly after reception in order to make dose adapatation of trifluridine/tipiracil if required.

- Sampling for PK/PD analysis (please refer to section 8.2.4 for timing details)
- Blood sampling for mandatory ancillary studies (please refer to section 8.8 for timing details)
- Tumoral sampling for mandatory ancillary studies (please refer to section 8.8 for timing details)

Please note that PK/PD samples and blood sampling will be performed only once for patient from level 1 included in level 2. PK/PD will be performed at C0 of level 1 and blood sampling will only be performed at C1 from level 2.

8.2.3 Paraclinical examinations during the treatment period of phase I

- Tumor evaluations will be done by imaging every 8 weeks (+/-7 days) during the first 12 months of treatment or until disease progression (whichever occurs first), and then every 12 weeks (+/-7 days) until progression. CT scan of the chest, abdomen and pelvis is required. MRI could be performed in the case of CTscan is not indicated but same method of evaluation should be kept. Study recommends CT scans. Note that radiological assessments should not be repeated if they were obtained less than 6 weeks from withdrawal of therapy. Other appropriate radiological assessments are left to the investigator’s discretion (e.g bone scan or brain scan if known metastases or if clinically indicated).
- Verification of heart functions by electrocardiogram (ECG) is at the investigator discretion as clinically indicated during the treatment period.
- Health-related quality of life survey EORTC-QLQ-C30 and CR29 will be completed every 8 weeks

8.2.4 Pharmacokinetics and pharmacodymacis sampling for phase I

In order to assess Pharmacokinetics (PK) one heparin tube of 6ml will be collected at 7 time points :

1- day 1 before infusion
2- day 1≈ 30 minutes post infusion (+/-10 minutes)
3- day 2
4- day 5
5- day 8
6- day 15 before XB2001 adminsitration
7- day 29 before XB2001 administration

Plasma will be isolated at each sampling time and frozen. PK measurement will be carried out during the phase 1 by quantitative indirect ELISA for XB2001 (Anti IL-1α) Concentration Measurement in Plasma. Performed by the “plateforme de transfert en biologie du cancer” (PTBC) from the CGFL of Dijon.
To assess immunogenicity, anti-drug antibody responses will be measured using a proprietary sandwich ELISA developed by XBiotech. Patient plasma was incubated on MABp1-coated microplates followed by labelled secondary antibodies. Measurements were taken at the same timepoints as for pharmacokinetic data and performed by the “plateforme de transfert en biologie du cancer” (PTBC) from the CGFL of Dijon.
Dosage of IL-6 using ELISA will be performed on Day 1 (30 min post infusion), Day 15 and day 29 samples.

8.3 Visits and assessment during the treatment period of phase II

The study flow chart of the study presented in **table 2** list the assessments and the visit schedule. A general +/-3 days window is allow for assessments or visits.
First cycle (first treatment administration) should be performed at the latest 3 days after randomization.
All examinations revealing a toxicity related to one of the investigational products must be periodically repeated until toxicity disappearance (or until considered irreversible).
The treatment phase will continue until confirmation of disease progression or discontinuation of the treatment. A visit will be scheduled on site every cycle (every 28 days). At every visit, patient will be assessed as follows:

8.3.1 Clinical examinations and assessments during the treatment period of phase II

- Evaluation of toxicities according to NCI-CTCAE version 5.0
- Physical examination as clinically indicated
- ECOG performance status
- Vital signs: blood pressure, pulse, body temperature and respiratory rate
- Review and collection of concomitant treatments

8.3.2 Biological tests during the treatment period of phase II

The following biological tests must be done at each cycle

- Clinical chemistry
- Hematology
- Urine analysis
- Pregnancy test
- ACE

Blood count results and biochemistry results must be obtained within 72 hours of the planned visit. Investigator should document the review and the evaluation of those biological reports promptly after reception in order to make dose adapatation of trifluridine/tipiracil if required.

Particularity/point of vigilance: Hematology and clinical chemistry will be performed on **Day 1** (or until 3 days before the treatment initiation) and **at Day 12 (+/- 0 day)** of the **cycle 1** at the site hospital in order to monitor biological parameters.

- Blood sampling for mandatory ancillary studies (please refer to section 8.8 for timing details)
- Tumoral sampling for optional ancillary studies (please refer to section 8.8 for timing details)

8.3.3 Paraclinical examinations during the treatment period of phase II

- Tumor evaluations will be done by imaging every 8 weeks (+/-7 days) during the first 12 months of treatment or until disease progression (whichever occurs first), and then every 12 weeks (+/-7 days) until progression. CT scan of the chest, abdomen and pelvis is required. MRI could be performed in the case of CTscan is not indicated but same method of evaluation should be kept. Study recommends CT scans. Note that radiological assessments should not be repeated if they were obtained less than 6 weeks from withdrawal of therapy. Other appropriate radiological assessments are left to the investigator's discretion (e.g bone scan or brain scan if known metastases or if clinically indicated).
- Verification of heart functions by electrocardiogram (ECG) is at the investigator discretion as clinically indicated during the treatment period.
- Health-related quality of life survey EORTC-QLQ-C30 and CR29 will be completed every 8 weeks

8.4 End-of-treatment assessments for phase I and phase II

The end of treatment visit should take place 28 days (+/-7 days) after the discontinuation of treatment either due to disease rpogression or due to other reason indicated in section 4.4.4. or if the patient has withdrawn from the study and contents to performing this visit. The following assessments will be performed at this visit:

8.4.1 Clinical examination for End-of-treatment assessments for phase I and phase II

- Evaluation of toxicities according to NCI-CTCAE version 5.0
- Physical examination as clinically indicated
- ECOG performance status
- Vital signs: blood pressure, pulse, body temperature and respiratory rate
- Review and collection of concomitant treatments

8.4.2 Biological tests for End-of-treatment assessments for phase I and phase II

The following biological tests must be done at the end of treatement visit:

- Clinical chemistry
- Hematology
- Urine analysis
- Pregnancy test
- ACE
- Blood sampling for mandatory ancillary studies (please refer to section 8.8 for timing details)

8.4.3 Paraclinical examinations examination for End-of-treatment assessments for phase I and phase II

- Tumor evaluations will be repeated if they were obtained less than 6 weeks from withdrawal of therapy
- Verification of heart functions by electrocardiogram ECG will be performed
- Health-related quality of life survey EORTC-QLQ-C30 and CR29 will be completed

8.5 Post treatment follow-up visits for phase I and phase II

Patient will be followed up every 12 weeks (+/-7 days) for a maximum of 2 years after treatment discontinuation.

Patients who withdrew from the study for other reasons than disease progression, tumor assessments with RECIST 1.1 evaluation should be continued to be documented every 8 weeks (+/- 7 days) during the first 12 months from the initiation of the treatment phase, and then every 12 weeks (+/-7 days) until disease progression or initiation of another cancer treatement.

Every 12 weeks after end of treatment visit, the following information will be collected:

- Disease and survival status: information may be collected during vising performed on the site for standard care or by phone calls documented in the source data of patient.
- Collection of persistent or long term occuring toxicities
- New cancer treatment
- Tumor assessment (if applicable)

Ongoing toxicities or AEs should be monitored until resolution or returned baseline level.

Information concerning adverse events occuring during the 30 days after the last administration of XB2001/placebo + trifluridine/tipiracil must be collected. Any late Seriou Adverse Drug Reaction (SAE related to the association of trifluridine/tipiracil + XB2001/placebo) at any time after this period must be reported to the pharmacovigilance of the study R&D UNICANCER.

8.6 Treatment after study interruption for phase I and phase II

After documented disease progression or discontinuation of treatement, the patient’s treatment will be at the investigator’s discretion.

8.7 Emergency unblinding for phase II

Subjects and investigators will be unblinded to their treatment allocation after database lock. In the event of an emergency that would require the investigator to be aware of the treatment allocation prior to the end of the trial, the investigator can obtain this information, on a per subject basis, after consultation with the Sponsor’s. Every effort should be made to consult with the Sponsor prior to emergency unblinding. Events that qualify for emergency unblinding are as follows:

• A grade 3 or greater AE which are “probably or definitely” related to XB2001 study drug AND only if treatment assignment information is deemed essential for the management of the event. This type of reaction would require that the patient receive no further doses and is followed until the resolution of the toxicity.

• Any suspected, unexpected, serious adverse reaction (SUSAR) AND only if treatment assignment information is deemed essential for the management of the event.

• Pregnancy. Subject may be able to continue on study if pregnancy is terminated and PI approves continuation in study.

8.8 Ancillary studies blood and tumor sampling for phase I and phase II

8.8.1: Tumor samples

Baseline tissue for ancillary studies are mandatory for patient from phase I and phase II and included in the principal informed consent form. A second biopsy is optional for patients from phase I and phase II depending on

a specific optional ICF that should be signed from the patient before any procedure for ancillary studies are performed.

- Tumor samples at inclusion (**Mandatory**)

Archive tumor samples from primary or secondary site of their current disease less than 2 years old will be collected at baseline. These blocks should undergo quality review, prior to evaluation or shipment. Each sample should be reviewed for:
− Adequate fixation
− Good preservation of morphology
− Presence of tumor tissue
− Histopathology consistent with indication
− Greater than 100 tumor cells are required to determine PD-L1 status – tumor cell content must be reviewed prior to testing in order to obtain a valid result for PD-L1.

Where it is not possible or indicated to ship the block, unstained slides should be prepared from the paraffin-embedded tumor sample block.
− 15 to 20 slides of 4 micron (µm) thick, unstained sections should be provided
− A new disposable microtome blade must be used for each block to prevent contamination between patient samples
− Slides are stable under these conditions for 6 months.
− Apply one section per slide to positively-charged Superfrost glass slides
− The sections should be dried overnight between room temperature and 37°C. Do not dry sections at temperatures above 37°C.

Sections should be stored at ambient temperature and protected from light until use or shipment to testing lab by courier at ambient temperature. It is recommended that slides are cut freshly prior to testing and they are used within 90 days of being cut to obtain status.

- Tumor samples after 8 weeks of treatment (**Optional)**:

An optional biopsy from secondary or primary tumor site will be required after 8 weeks of treatment with 2 biopsy cores.

The paraffin-embeded block will be send to the sponsor with a dedicated carrier. Where it is not possible or indicated to ship the block, unstained slides should be prepared from the paraffin-embedded tumor sample block.

− A minimum of 10 of 4 micron (µm) thick, unstained sections should be provided

− A new disposable microtome blade must be used for each block to prevent contamination between patient samples

− Slides are stable under these conditions for 6 months.

− Apply one section per slide to positively-charged Superfrost glass slides

− The sections should be dried overnight between room temperature and 37°C. Do not dry sections at temperatures above 37°C.

Sections should be stored at ambient temperature and protected from light until use or shipment to testing lab by courier at ambient temperature. It is recommended that slides are cut freshly prior to testing and they are used within 90 days of being cut to obtain status.

TUMORAL SAMPLES				
	**Quantité**	**Phase I Phase II**	**Timing**	**Utilisation**
Tumoral samples dated less than 2 years or fresh biopsy **MANDATORY**	Preferred the paraffin block or 15 to 20 white blades of 4µm	Phase I and phase II	At inclusion	Immunostaining
Biopsie fraîche **OPTIONAL**	Paraffin block	Phase I and phase II	• C3	Immunostaining

8.8.2: Blood samples

Blood sampling for ancillary studies are mandatory for patient from phase I and phase II and included in the principal informed consent form.

The table below summarized the sampling to be performed.

BLOOD SAMPLES				
**Type of tube**	**Volume**	**Phase I Phase II**	**Timing**	**Utilisation**
EDTA<br>**MANDATORY**	5 tubes of 10ml	Phase I and Phase II	• C1D1<br>• C3D1<br>• C5D1<br>• EOT	4 tubes= PBMCs for Immunomonitoring<br>1 tube = ADNc isolation
Heparin<br>**MANDATORY**	1 tube of 4mL or 6mL	Phase I and Phase II	• C1D1<br>• C3D1<br>• C5D1<br>• EOT	Plasma<br>Cytokines dosing
Dry tube<br>**MANDATORY**	1 tube of 4mL	Phase I and Phase II	• C1D1<br>• C3D1<br>• C5D1<br>• EOT	Serum<br>Cytokines dosing
Heparin<br>**MANDATORY**	1 tube of 4mL or 6mL	Phase II only	• C1D12	Plasma<br>trifluridine/tipiracil dosing

9. SAFETY EVALUATION

The Principal Investigator is responsible for ensuring that all staff involved in the study is familiar with the content of this section.

In this study, AEs and SAEs will be collected from the time of the patient signing the informed consent and AEs from the time until the follow-up period is completed (30 days after the last dose of treatment trifluridine/tipiracil +/- XB2001/placebo). If an event that starts post the defined safety follow up period noted above is considered to be due to a late onset toxicity to study drug then it should be reported as an AE in the eCRF or SAE as applicable.

During the course of the study, all AEs and SAEs should be proactively followed up for each patient for as long as the event is ongoing. Every effort should be made to obtain a resolution for all events, even if the events continue after the patient has discontinued study drug or the study has completed.

Any AEs that are unresolved at the patient's last visit in the study are followed up by the Investigator for as long as medically indicated, but without further recording in the eCRF.

9.1 Adverse event

9.1.1 General definition

An adverse event is defined as any untoward medical occurrence, in a patient or clinicaly study subject treated by a medicinal product without necessarily a causal relationship with the treatement. An AE can be any unfavorable or unintended sign (including an abdormal laboratory finding, for example), symptom or disease temporammy associated with the use of a medicinal product or protocol-specified procedure, whether or not considered related to the medicinal product or protocol-specified procedure, whether or not considered related to the medicinal product or protocol-specified procedure. In addition, any worsening (i.e any clinically significant adverse change in frequency or intensity) of a pre-existing condition that is temporally associated with the use of the investigational product, is also considered as an AE. Progression of the disease under study is not considered as AE.

All subjects will be monitored for AEs during the study. Assessments may include monitoring of any or all of the following parameters: the subject's clinical symptoms, laboratory, pathological, radiological or surgical findings, physical examination findings, or findings from other tests and procedures.

The term AE is used to include both serious and non-serious AEs. All patients who are permanently discontinued from receiving investigational product will be followed.

9.1.2 Evaluating Adverse Events

As far as possible, each AE should be evaluated to determine:

- ✓ The severity of the event
- ✓ The AEs relationship (related or not related)
- ✓ The AEs duration (onset date end dates or ongoing if continuing at final examination)
- ✓ Action taken (corrective treatment, hospitalisation, prolongation of hospitalisation,…)
- ✓ The seriousness of the event (section 9.2.1)

- **Severity**

For each episode of an AE, all changes to the CTCAE grade attained, as well as the highest CTC grade, are to be reported.

It is important to distinguish between seriousness and severity. Severity is a measure of intensity whereas seriousness is defined by the criteria in Section 9.2.1. An AE of severe intensity need not necessarily be considered serious. For example, nausea that persists for several hours may be considered severe nausea, but not a SAE unless it meets the criteria shown in Section 9.2.1. On the other hand, a stroke that results in only a limited degree of disability may be considered a mild stroke but would be an SAE when it satisfies the criteria shown in Section 9.2.1.

Assessment of severity is one of the responsibilities of the investigator in the evaluation of AEs and SAEs. Severity will be graded according to the NCI CTCAE v5. The determination of severity for all other events not listed in the CTCAE should be made by the investigator based upon medical judgment and the severity categories of Grade 1 to 5 as defined below.

- ✓ **Grade 1 (mild)**: asymptomatic or mil symptoms; clinical or diagnostic observations only; intervention not indicated.
- ✓ **Grade 2 (moderate):** minimal local or non-invasive intervention indicated; limiting age-appropriate instrumental activities of daily living (ADL).
- ✓ **Grade 3 (severe):** medically significant but not immediately life-threatening; hospitalisation or prolongation of hospitalisation indicated; disabling; limiting self-care ADL.
- ✓ **Grade 4 (life threatening):** urgent intervention indicated.
- ✓ **Grade 5 (fatal)** Death (loss of life) as a result of an event.

The grading scales found in the revised NCI CTCAE version 5 will be utilized for all events with an assigned CTCAE grading. For those events without assigned CTCAE grades, the recommendation in the CTCAE criteria

that converts mild, moderate, and severe events into CTCAE grades should be used. A copy of the CTCAE version 5 can be downloaded from the Cancer Therapy Evaluation Program website (http://ctep.cancer.gov).

Events, which are unequivocally due to disease progression, should not be reported as an AE during the study

- **Relationship**

The investigator must analyse every AE and try and determine the cause, duration and whether the AE is related to the IP(s) or study procedures.

- **Duration**

Adverse events must be followed until thy have resolved, stabilized, or returned to baseline levels.

9.1.3 Adverse Event Reporting

All AEs that occur from the time of the inclusion until 30 days after the last dose of trifluridine/tipiracil +/- XB2001 must be reported by the investigator. All AEs will be recorded on the AE page of the eCRF and documented in the subject's source documents.

Deterioration as compared to baseline in protocol-mandated laboratory values and vital signs should only be reported as AEs if the investigator considers the event clinically significant or if they fulfil any of the SAE criteria or are the reason for discontinuation of treatment with the IPs.

If deterioration in a laboratory value or vital sign is associated with clinical signs and symptoms, the sign or symptom will be reported as an AE and the associated laboratory result or vital sign will be considered as additional information. Whenever possible, the reporting Investigator should use the clinical rather than the laboratory term (e.g anemia versus low haemoglobin value). In the absence of clinical signs or symptoms, clinically relevant deteriorations is non-mandated parameters should be reported as AEs.

Deterioration of a laboratory value that is unequivocally due to disease progression should not be reported as an AE/SAE.

Any new or aggravated clinically relevant abnormal medical finding at a physical examination as compared with the baseline assessment will be reported as an AE.

9.1.4 Identified and potential risks of XB2001

More than 1,100 patients have been treated using a previous anti-IL-1α antibody in patients with advanced solid tumors, advanced hematologic malignancies, metastatic colorectal cancer, peripheral vascular disease, type II diabetes, acne vulgaris, plaque psoriasis, pyoderma gangrenosum, atopic dermatitis and hidradenitis suppurativa. No antibody-related toxicities have been across the indications.

Over 2,000 doses (N=309) of previous anti-IL-1α antibody were administered at 7.5 mg/kg to refractory, metastatic CRC patients with cancer associated symptoms at baseline (ECOG performance status 1 and 2). In this study the most common AEs reported (>10%) were:

- Abdominal pain
- Peripheral edema
- Fatigue
- Anemia
- Constipation
- Decrease in weight
- Asthenia
- Decreased appetite
- Nausea.

The majority of these events were grade 1 or 2 and appeared to be related to the underlying CRC. The prevalence of these events was similar in the treatment and placebo groups.

Two infusion reactions were reported in this trial, and they were not serious or severe (grade I or II).

XB2001 is a recombinant human derived IgG4 monoclonal antibody specific for human interleukin-1α (IL-1α). As such, it is an immunomodulator that has anti-inflammatory and anti-neoplastic properties. Other agents that could be considered in the same pharmacologic class include biologic agents that target IL-1 receptor and IL-1β. Potential risks for agents in this class include infusion or injection site reactions and risk of infection.

XB2001 is defined as a True Human monoclonal antibody. Unlike previous generations of humanized or fully human antibodies, the entire XB2001 heavy and light chain sequences are identical to those found in naturally-occurring human IgG4κ. The heavy and light chain variable regions of XB2001 are identical to those originally expressed by a peripheral blood B lymphocyte that was obtained from a healthy individual.
No in vitro affinity maturation or modifications were made to improve the natural binding affinity (~60 pM) of XB2001. A true human antibody should be effectively non-immunogenic in humans and thus exhibit optimal activity and pharmacokinetics.

The mechanism behind infusion reactions is not clear in all cases. It may involve a reaction against the antibody products themselves, or, against some minor residual component from the manufacturing process (i.e. host cell proteins). To date, after administration to over 1,100 patients, and over 5,000 doses across all trials, the risk of infusion reactions with True Human antibodies remains extremely low. Newertheles, since there is a historic infusion reaction risk with previous generation antibodies monitoring is encouraged for at least 1 hour after the end of the initial XB2001 infusion. Availability of resuscitation equipment must be ensured. Pre-medication with antihistamines or corticosteroids is not required.

For the purposes of expedited safety reporting in clinical trials, **the following should be considered expected events:**
• Infusion Related Reactions
• Injection Site Reactions

9.1.5 Identified and potential risks of trifluridine/tipiracil

The most serious side effects seen in patients receiving trifluridine/tipiracil are **myelosuppression** and **gastrointestinal toxicity**.

The most commonly observed side effects (≥ 30%) in patients receiving trifluridine/tipiracil are neutropenia (53% [34% ≥ Grade 3]), nausea (34% [1% ≥ Grade 3]), fatigue (32% [4% ≥ Grade 3]), anemia (32% [12% ≥ Grade 3]). The most common adverse reactions (≥ 2%) resulted in discontinuation of treatment, dose reduction, postponement or interruption of administration are **neutropenia**, **anemia**, **leukopenia**, **fatigue**, **thrombocytopenia**, **nausea** and **diarrhea**.

Adverse reactions reported in clinical studies in patients treated with trifluridine/tipiracil:

- **<u>Very common (≥ 1/10)</u>**

✓ Neutropenia, leukopenia, anemia, thrombocytopenia
✓ Decreased appetite
✓ Diarrhea, nausea, vomiting
✓ Tired

- **<u>Frequent (≥ 1/100, < 1/10)</u>**

✓ Lower respiratory tract infection
✓ Febrile neutropenia, lymphopenia
✓ Hypoalbuminemia
✓ Dysgeusia, peripheral neuropathy
✓ Dyspnea
✓ Abdominal pain, constipation, stomatitis, mouth problems
✓ Hyperbilirubinemia
✓ Palmar-plantar erythrodysesthesia, rash, alopecia, pruritus, dry skin
✓ Proteinuria
✓ Fever
✓ Edema, inflammation of the mucous membranes

- ✓ Malaise
- ✓ Elevated liver enzymes, elevated blood alkaline phosphatase
- ✓ Loss of weight

- **<u>Uncommon (≥1 / 1,000, <1/100)</u>**

- ✓ Septic shock, infectious enteritis, lung infection, biliary tract infection, flu, urinary tract infection, gingivitis, shingles, intertrigo inter-toes (athlete's foot), candida, bacterial infection, infection, neutropenic sepsis, infection of the tract upper respiratory tract, conjunctivitis
- ✓ Cancer pain
- ✓ Pancytopenia, granulocytopenia, monocytopenia, erythropenia, leukocytosis, monocytosis
- ✓ Dehydration, hyperglycemia, hyperkalaemia, hypokalaemia, hypophosphatemia, hypernatremia, hyponatremia, hypocalcaemia, gout
- ✓ Anxiety, insomnia
- ✓ Neurotoxicity, dysesthesia, hyperesthesia, hypoaesthesia, syncope, paraesthesia, feeling of burning, lethargy, dizziness, headache
- ✓ Decreased visual acuity, blurred vision, diplopia, cataracts, dry eye
- ✓ Dizziness, hearing discomfort
- ✓ Angina pectoris, arrhythmia, palpitations
- ✓ Embolism, hypertension, hypotension, flushing
- ✓ Pulmonary embolism, pleural effusion, rhinorrhea, dysphonia, oropharyngeal pain, epistaxis, cough
- ✓ Haemorrhagic enterocolitis, gastrointestinal haemorrhage, acute pancreatitis, ascites, ileus, subileus, colitis, gastritis, gastric reflux, esophagitis, gastric emptying disturbances, abdominal distension, anal inflammation, mouth ulceration, dyspepsia, reflux, gastroesophageal, proctalgia, oral polyp, gingival bleeding, glossitis, diseases, periodontal disease, dental problems, "Gagging", flatulence, bad breath
- ✓ Hepatotoxicity, biliary dilation
- ✓ Skin exfoliation, urticaria, photosensitivity reactions, erythema, acne, hyperhidrosis, blisters, onychopathies
- ✓ Joint swelling, arthralgia, bone pain, myalgia, pain, musculoskeletal, muscle weakness, muscle spasms, pain in extremity
- ✓ Renal failure, non-infectious cystitis, voiding disturbances, hematuria, leukocyturia
- ✓ Menstrual disturbances
- ✓ Altered general condition, pain, sensation of change in body temperature, xerosis, discomfort
- ✓ Blood creatinine increased, ECG QT prolongation, INR increased, increased activated partial thromboplastin time (aPTT), increased uremia, increase in blood lactate dehydrogenase, decrease in total protein, increase C reactive protein, decrease in hematocrit

9.1.5 Identified and potential risks of bevacizumab

The safety profile of bevacizumab is based on data collected during clinical studies conducted in more than 5.700 patients with different types of malignancies who were treated, for the most part, with bevacizumab combined with chemotherapy

The most serious side effects were:
• Gastrointestinal perforations
• Haemorrhage, including pulmonary haemorrhage/hemoptysis, more common in patients with non-small cell lung cancer
• Arterial thromboembolism
• Proteinuria
• Neutropenia
• Hypertension

- **<u>Very common (≥ 1/10)</u>**

  - ✓ Febrile neutropenia,
  - ✓ Leukopenia,

- ✓ Neutropenia,
- ✓ Thrombocytopenia
- ✓ Anorexia,
- ✓ Hypomagnesemia
- ✓ Hyponatremia
- ✓ Peripheral sensory neuropathy
- ✓ Dysarthria
- ✓ Headaches
- ✓ eye disease,
- ✓ Increased lacrimation
- ✓ Hypertension
- ✓ Thromboembolism
- ✓ Dyspnea, Rhinitis,
- ✓ Epistaxis,
- ✓ Cough
- ✓ Rectal bleeding,
- ✓ Stomatitis,
- ✓ Constipation,
- ✓ Diarrhea,
- ✓ Nausea,
- ✓ Vomiting,
- ✓ Abdominal pain
- ✓ Dysgeusia
- ✓ Complications of wound healing
- ✓ Exfoliative Dermatitis,
- ✓ Skin Dryness,
- ✓ Skin discoloration
- ✓ Arthralgia
- ✓ Myalgia
- ✓ Proteinuria
- ✓ Ovarian failure
- ✓ Asthenia
- ✓ Fatigue
- ✓ Fever
- ✓ Pain
- ✓ Inflammation of the mucous membranes
- ✓ Weightloss

- **<u>Frequent (≥ 1/100, < 1/10)</u>**

- ✓ Sepsis
- ✓ Abscess
- ✓ Cellulite
- ✓ Infection
- ✓ Urinary tract infection
- ✓ Anemia
- ✓ Lymphopenia
- ✓ Hypersensitivity, infusion-related reactions
- ✓ Dehydration
- ✓ Stroke
- ✓ Syncope,
- ✓ Drowsiness
- ✓ Congestive heart failure
- ✓ Supraventricular tachycardia

- ✓ Thromboembolism
- ✓ Hemorrhage
- ✓ Deep vein thrombosis
- ✓ Pulmonary hemorrhage
- ✓ Hemoptysis
- ✓ Pulmonary embolism
- ✓ Hypoxia
- ✓ Dysphonia
- ✓ Gastrointestinal perforation
- ✓ Intestinal perforation
- ✓ Ileus
- ✓ Bowel obstruction
- ✓ Rectovaginal fistulas
- ✓ Gastrointestinal disorders
- ✓ Proctalgia
- ✓ Palmar-plantar erythrodysesthesia syndrome
- ✓ Fistula
- ✓ Muscular weakness
- ✓ Back pain
- ✓ Pelvic pain
- ✓ Lethargy

- **Rare (≥1 / 1,0000, <1/1000)**

- ✓ Necrotizing fasciitis
- ✓ Anaphylactic shock
- ✓ Posterior reversible encephalopathy syndrome

- **Very Rare (<1/10000)**

- ✓ Hypertensive encephalopathy

- **Not known**
- ✓ Renal thrombotic microangiopathy
- ✓ Aneurysms and arterial dissections
- ✓ Pulmonary hypertension
- ✓ Perforation of the nasal septum
- ✓ Gastrointestinal ulcer
- ✓ Gallbladder perforation
- ✓ Osteonecrosis of the jaw
- ✓ Non-mandibular osteonecrosis
- ✓ Fetal abnormalities

9.2 Serious adverse events

9.2.1 General definition

A serious adverse event is an AE occurring during any study phase (i.e., screening, run-in, treatment, wash-out, follow-up), at any dose of the study drugs that fulfils one or more of the following criteria:

- ✓ Results in death
- ✓ Is immediately life-threatening
- ✓ Requires in-patient hospitalization or prolongation of existing hospitalization
- ✓ Results in persistent or significant disability or incapacity
- ✓ Is a congenital abnormality or birth defect in offspring of the patient
- ✓ Is an important medical event that may jeopardize the patient or may require medical intervention to prevent one of the outcomes listed above:

Theses characteristics/consequences have to be considered at the time of the event. For example, regarding a life-threatening event, this refers to an event in which the subject was at risk of death at the time of the event ; it does not refer to an event which hypothetically might have caused death if it was more severe.

The terms disability and incapacity correspond to any clinically relevant physical or psychic handicap, transient orpermanent, with impacts on the physical condition/activity and/or the quality of life of patient.

Medical and scientific judgment should be exercised in deciding whether other situations should be considered serious, such as important medical events that might not be immediately life-thratening or result in death or hospitalization, but might jeopardize the patient or might require intervention to prevent one of the other outcomes listed in the definition above (for exemple: overdose, second cancer,…).

The assessment of whether there is reasonable causal relationship is made by the investigator. If the sponsor disagrees with the investigator’s causality assessment, the opinion of both the investigator and the sponsor should be provided with the report.

Serious adverse events will be recorded from time of signature of informed consent, throughout the treatment period including the follow-up period (30 days after last dose of XB2001).

During the course of the study all AEs and SAEs should be proactively followed up for each patient. Every effort should be made to obtain a resolution for all events, even if the events continue after discontinuation/study completion.

The investigator is responsible for following all SAEs until resolution, until the patient returns to baseline status, or until the condition has stabilized with the expectation that i twill remain chronic, even if this extends beyond study participation.

The following events are considered as SAEs but should not be managed according to section 9.2.6. The events do not require immediate reporting and should only be reported in the CRF.

- Hospitalization less than 24 hours,
- An hospitalization programmed before the beginning of the trial and / or planned by the protocol (biopsy, chemotherapy, etc.),
- Hospitalization for exploration not related to a change in the patient's condition (eg routine colonoscopy)
- Hospitalization occuring in the context of tumor progression of the disease under trial,
- A circumstance of life that has no impact on the state of health and does not require any medical / surgical intervention (for example, prolongation of hospitalization while waiting for a place in a reeducation establishment, loss of home, hospitalization for cause unavailable caregivers, ...)
- A pathology present before inclusion or discovery at inclusion (for example, diabetes discovered at baseline and requiring hospitalization in endocrinology, etc.)
- Hospitalization for reasons of comfort or social reasons,
- Elective hospitalization not associated with a worsening of the clinical state and unrelated to the objective of the clinical study and occurring during the clinical study.
- Any hospitalization and death related to cancer progression,
- Any hospitalization and death that occurs beyond the 30-day notification period excepted if related to IP,
- Any event that falls within the standard practice (treatment, evolution of the disease ....)

9.2.2 Other events requiring reporting

✓ <u>Deaths</u>

All deaths that occur during the study or within the protocol defined 30-day post last dose of trifluridine/tipiracil+/-XB2001/placebo safety follow-up period must be reported to PV R&D UNICANCER as follows:

- Death that is clearly the result of disease progression should be documented but should not be reported as an SAE
- Where death is not due (or not clearly due) to progression of the disease under study, the AE causing the death must be reported to pharmacovigilance and R&D unit of UNICANCER as a SAE immediatly. The report

should contain a comment regarding the co-invovlvement of progression of disease, if appropriate and should assign main contributory cause of death.

- Death with an unknown cause should always be reported as a SAE.

✓ Overdose

Use of XB2001/placebo in excess of the specified in the protocol is considered to be an overdose. There is currently no specific treatment in the event of overdose of XB2001/placebo and possible symptoms of overdose are not established. No serious consequences in the event of accidental overdose is excepted. However:

- An overdose with associated AEs will be recorded as the AE diagnosis or symptoms in the relevant AE modules of the CRF
- An overdose without associated symptoms will only be reported in the compliance information of the eCRF

If an overdose is observed during the study with XB2001/placebo, the investigator or the site personnel will inform the pharmacovigilance and R&D unit of UNICANCER immediatly.
The designated UNICANCER representative will work with the investigator to ensure that all relevant information is provided to the sponsor.
For overdose associated with an SAE, the standard reporting timelines apply. For other overdoses, reporting must occur within 30 days.

✓ Pregnancy

All pregnancies and outcomes of pregnancy should be reported to pharmacovigilance and R&D unit of UNICANCER except for:

- Pregnancy discovered before the study patient received and study drugs
- Pregnancy of a female partner of male patient, providing there is no restriction of male patient fathering a child

✓ Any Grade ≥ 3 adverse event due to XB2001 including injection site reaction

Those events must be reported within 24 hours of learning of the event.

9.2.3 Maternal exposure

If a patient becomes pregnant during the course of the study, the IPs should be discontinued immediately.

Pregnancy itself is not regarded as an AE unless there is a suspicion that the IP under study may have interfered with the effectiveness of a contraceptive medication. Congenital abnormalities or birth defects and spontaneous miscarriages should be reported and handled as SAEs. Elective abortions without complications should not be handled as AEs. The outcome of all pregnancies (spontaneous miscarriage, elective termination, ectopic pregnancy, normal birth, or congenital abnormality) should be followed up and documented even if the patient was discontinued from the study.

If any pregnancy occurs in the course of the study, then the Investigator or other site personnel should inform the the pharmacovigilance and R&D unit of UNICANCER representatives within 1 day, i.e., immediately, but no later than 24 hours of when he or she becomes aware of it.

The designated Unicancer representative will work with the Investigator to ensure that all relevant information is provided to the AstraZeneca Patient Safety data entry site within 1 to 5 calendar days for SAEs and within 30 days for all other pregnancies.

The same timelines apply when outcome information is available.

9.2.4 Paternal exposure

Male patients should refrain from fathering a child or donating sperm during the study and for 30 days following the last dose of trifluridine/tipiracil + XB2001/placebo.

Pregnancy of the patient's partner is not considered to be an AE. However, the outcome of all pregnancies (spontaneous miscarriage, elective termination, ectopic pregnancy, normal birth, or congenital abnormality)

occurring from the date of the first dose until 30 days after the last dose of trifluridine/tipiracil + XB2001/placebo should be reported to the sponsor.

Where a report of pregnancy is received, prior to obtaining information about the pregnancy, the Investigator must obtain the consent of the patient's partner.

9.2.5 Suspected unexpected serious adverse reaction (SUSAR)

A SUSAR is defined as any serious adverse reaction, for which nature, severity or outcome is not consistent with the applicable drug information (e.g Investigator's Brochure for an unapproved investigational product package insert/summary of product characteristics for an approval product).
The safety reference documents for this study to assess whether an AE is expected or unexpected are:

- XB2001 most recent version of investigator brochure
- Trifluridine/tipiracil: most recent Summary of Product Characteristics (SmPC)

9.2.6 Measures to be taken in case of a serious adverse event

All SAEs will be reported, whether or not considered causally related to the investigational product, or to the study procedure(s). All SAEs will be recorded in the eCRF.

The reporting period for SAEs is the period immediately following the time that written informed consent is obtained until 30 days after the last dose of trifluridine/tipiracil + XB2001/placebo or until the initiation of alternative anticancer therapy.

The investigator ensures that adequate medical care is provided to the patient.

The investigator must **immediately** following knowledge of the event, notifies the R&D UNICANCER pharmacovigilance unit of any SAE or any new event defined here above, whether related or not related to the research, which occurs during the "study reporting period". This reporting period :

- starts at the date of the signature of informed consent form
- Covers the entire period during which the patient is receiving the investigational treatment or is subject to specific procedures related to the study
- Covers a period up until 30 days after the last administration of trifluridine/tipiracil +/ XB2001/placebo study drug or until the initiation of alternative anticancer therapy.

Any later SAE occurring after the study reporting period defined above, and which is considered to be related to the experimental treatment(s) or to the research (other treatment used, diagnostic procedures and examinations carried out during the research) must be reported without any limitation in terms of deadline.

Notification must be carried out immediately by fax or email to R&D UNICANCER pharmacovigilance unit by sending the form "notification of SAE", located in the investigator files, completed as precisely as possible, dated and signed by the physician-investigator:

R&D UNICANCER – Pharmaco vigilance
Phone : 01 44 23 04 16 / FAX : 01 44 23 55 70
Email : pv-rd@unicancer.fr

Abnormal laboratory results should be reported as SAE if they possibly put at risk the patient or they require medical intervention to prevent an outcome corresponding to one of severity criteria.

Second cancer should be reported as a SAE whether or not related to the research must be reported to R&D UNICANCER pharmacovigilance unit without any limitation in terms of deadline.

The investigator shall send additional information to the R&D UNICANCER pharmacovigilance unit using a SAE declaration form by ticking the "Follow-up box" to specify that it is a Follow-Up and not an initial report, as soon as he is aware of the event. He shall also submit the last follow-up at the resolution or stabilization of the SAE.

The investigator is responsible for appropriate medical follow-up of patients until resolution or stabilization of the event or until death of the patient. This can sometimes mean that follow-up continues after the patient has left the study.

The investigator must keep the documents concerning the suspected SAE in order to supplement the information previously submitted if necessary.

Requests for clarification and/or additional information may be sent to the investigator by the R&D UNICANCER pharmacovigilance unit or CRA sponsor of the trial to document and treat the case.

All documents and information sent to the R&D UNICANCER pharmacovigilance unit or CRA sponsor should be pseudonymised.

10. BLOOD AND TUMOR SAMPLES ANALYSIS

Pharmacokinetics, pharmacodynamics and ancillary studies will offer an unbiased and unique opportunity to learn more about the molecular activity and mechanisms of action of these compounds and to identify predictive biomarkers.

10.1 Peripheral immunomonitoring (blood samples – mandatory for patients from phase I and phase II)

At each time point (i) before intiation of treatment (ii) 8 weeks after treatment initiation (iii) 6 months after treatment initiation (iv) at progression; blood samples will be collected to isolate PBMCs and plasma for cytokine/chimiokine analysis and plasma for circulating tDNA analysis.

- PBMCs

The profile of effector and suppressor immune cells will be determined from frozen PBMC by multicolor flow cytometry.

- For MDSC identification, we will use lineage cocktail (CD3, 56, 19, and 20), CD33, CD15, CD14, and HLA-DR antibody.

- For Treg analysis, we will use CD4, CD45RA, CD25, and Foxp3 antibodies and for other Thelper subsets, we will use CD4, CD45RA, CD25, CCR6, CXCR3 and CRTH2 antibodies. For T CD8 subsets, we will use CD3, CD8, CD45RA, CD44, CCR7, CD62L, PD1, TIM3, LAG3 and TIGIT antibodies.

- To analyse the functionality of CD4 and CD8 T cells, we will perform PMA/Iono activation on PBMCs and then carry out an intracellular staining xith the following antibodies: CD3, CD4, CD8, CD25, Foxp3, IFNγ, TNFα, IL-2, IL-4, IL-17A.

- Assessment of NY-ESO1 and telomerase specific T-cell responses: We select these two shared antigens (NY-ESO1 and telomerase), frequently overexpressed in colon cancer. Presence of specific T cells, which recognize these 2 antigens are frequently observed in colorectal cancer patients. Peripheral blood mononuclear cells (PBMC) will be isolated by density centrifugation on Ficoll-Hyperpaque gradients (Sigma-Aldrich). PBMC will be frozen and stored until the end of the trial. Analysis will be performed when all samples will be collected. Telomerase and NY-ESO1-specific T-cell responses will be assessed by standardized IFN-γ ELISPOT assay (Diaclone, France) as previously reported (40). Briefly PBMCs from cancer patients will be cultured with 5µmol/L of pool of Telomerase of NY-ESO1 peptide in a 96-well ELISPOT plate (2.105 cells per well) in triplicates, with or without UCP peptides in X-vivo 15 medium. After 24 hours of culture, the presence of UCP-specific T cells will be assessed according to the manufacturer's instructions. The specific IFN-γ-secreting cells will be counted using the C.T.L. Immunospot reader system.

- Plasma (obtained from the heparin tube)

Plasma will be collected to perform multiplex cytokine analysis. A 20 plex assay will be performed (sE-Selectin; GM-CSF; ICAM-1/CD54; IFN alpha; IFN gamma; IL-1α; IL-1β; IL-4; IL-6; IL-8; IL-10; IL-12p70; IL-13; IL-17A/CTLA-8 ; IP-10/CXCL10; MCP-1/CCL2; MIP-1α/CCL3; MIP-1β/CCL4; sP-Selectin; TNFα). In addition, IL1RA will be tested. CEA tumor marker and circulating DNA will be collected at the same time points. The samples will be centrally sent to the plateforme de transfert en biologie du cancer (PTBC) from the CGFL site at the end of the study to performe analysis.

- Serum (obtained from dry tube)

Serum will be collected to perform Inflammtory markers analysis (e.g., IL1RA, IL6, IL33). The serum samples will be sent to XBiotech in Austin Texas to perform those analysis at the end of the study.

- Plasma (obtained from one of the EDTA tubes)

Circulating cell-free DNA will be extracted from plasma by using the Maxwell ccfDNA Plasma Kit (Promega, France). DNA will be quantified with the Qubit and sequenced using a capture (Truesight cancer) kit on a Nexseq 500 (Illumina) following the manufacturer's recommendations. The FASTQs sequencing data will be aligned to the human genome (hg19). Samples will be analyzed after BAM recalibration using a specific algorithm developed to detect allelic ratios <2%, the BPER method[44]. The samples will be centrally sent to the plateforme de transfert en biologie du cancer (PTBC) from the CGFL site at the end of the study to perform analysis.

10.2 Pharmacokinetics analysis (blood sample and handling – mandatory for patients from phase I only)

- Plasma (obtained frome one EDTA tube)

For Phase I part: XB2001 Pharmacokinetics (PK) one EDTA tube of 6 ml will be collected before XB2001 administration and at 6 time points post administration:

1- day 1 before infusion
2- day 1 ≈ 30 minutes post infusion (+/-10 minutes)
3- day 2
4- day 5
5- day 8
6- day 15
7– day 29

Plasma will be isolated at each sampling time and frozen. PK measurement will be carried out during the phase I by quantitative indirect ELISA for XB2001 (Anti IL-1α) Concentration Measurement in Plasma. PD measurements will be performed by quantitative indirect ELISA of Anti-IL6. Performed by the "plateforme de transfert en biologie du cancer" (PTBC) from the CGFL of Dijon.

10.3 Dosing of trifluridine/tipiracil (mandatory for patients from phase II only)

For Phase II part: Plasma will be collected to measure trifluridine concentration on day 12 of the first cycle. A 4 mL heparin tube should be drawn at any time on D12. The blood sample should be centrifuged (1500 rpm, 10 min) and plasma should be stored immediately at at least -15°C. Plasma is stable for a year at -15°C.
Exact date and time of last trifluridine/tipiracil intake and blood sample should be collected. Medication history intake should also be recorded. The samples will be centrally sent to Besançon at the end of the study to perform analysis.

10.4 Immunohistology procedure (tumor samples – initial mandatory and second optional for patients from phase I and phase II)

Two tumor samples will be analyzed. Tumor samples collected before tratement initiation (mandatory) and tumor samples after 8 weeks of treatment (optional).

4 µm thin tissue sections will be cut from formalin fixed paraffin embedded specimen. All IHC procedures will be performed using Benchmark apparatus (Ventana) and diaminobenzidine (DAB) as chromogenic revelator. Once stained and permanently mounted, slides will be numerized with Nanozoomer HT2.0 (Hammamatsu) at x20 magnification. Number of positive cells per area (μm^2) will be obtained using the recently released QuPath free software. Briefly, pathologist will handily select representative areas (3 zones) of approximately $1mm^2$ on digitalized slides. Then, within this area, positive cells will be determined with a DAB threshold using automated Qupath algorithms. The same algorithms (nuclei and DAB detections) will be used for every slide to have homogenized results. CD8, PD-L1, CD4, CD3, CD163 DC-Lamp, PNAD, CD20 will be assessed.

10.5 Methodology for statistical analysis of ancillary studies:

Patients, disease, immunological and genomic characteristics will be examined using the Chi-2 test or Fisher's exact test for qualitative variables and the Mann-Whitney test or Kruskal-Wallis test for continuous variables, as

appropriate. Correlations will be estimated using the Spearman's rank correlation coefficient and associated p-values. Survival probabilities will be estimated using Cox proportional hazards regression models (hazard ratios and 95% confidence intervals, Wald test). The prognostic role of the different variables will be evaluated using a Wald test.

To select the most pertinent variables among all the tested variables in a multivariate model, we will use models adapted to high dimensional data. More precisely, we will use the Lasso model (least absolute shrinkage and selection operator) which is a regression analysis method that performs both variable selection and prognostic value estimation in the high dimensional setting.

Control arm will be used to determine the predictive versus prognostic role of generated biomarkers.

11. STATISTICAL ANALYSIS PLAN

11.1 Required number of patients

Phase I portion

A 3+3 design will be used in order to establish MTD. 5 dosing levels will be explored:

- Level 1: XB2001 250 mg alone – only 2 administrations
- Level 2: XB2001 250 mg + trifluridine/tipiracil
- Level 3: XB2001 500mg + trifluridine/tipiracil
- Level 4: XB2001 1000mg + trifluridine/tipiracil
- Level 5 XB2001 500mg or 1000 mg+ trifluridine/tipiracil + bevacizumab

Toxicities will be described by grade, by toxicity categories and by cycle, then for all cycles for each dose level.

Patients evaluable for MTD should have received at least 80% of the dose of trifluridine/tipiracil and the 2 doses of XB2001 or have experienced a DLT.

Patients who cannot be evaluated for MTD for reasons other than treatment-related toxicity will be replaced.

The phase I will included a minimum of **15 patients and a maximum of 30 patients** (plus any pathients who cannot be evaluated for MTD). 6 patients should be treated at the RP2D.

Phase II

This is a multicenter, randomized (1:1 ratio), non-comparative, double-blind, phase II study with an experimental arm (Arm B) which will receive the combination of trifluridine/tipiracil + XB2001 and a control arm (Arm A) treated with the standard of care trifluridine/tipiracil +/- bevacizumab + placebo.

We anticipate a 7.1 month median survival for the control group (5) and a hazard ratio=0.60 which corresponds to a 6-month OS rate of 55% in the control arm. We are projecting a 6-month OS of 70% in the experimental arm. Using a 2-stage Simon design, with 1-sided alpha risk=5%, power=85% and a 5% of patients lost to follow-up or consent withdrawal, **80 patients will be required in each arm (160 patients in total)**. The interim analysis will be performed on 26 evaluable patients according to the ITT population definition.

11.2 Definition of study population analysis

The three following populations will be defined for the statistical analysis:

- **Modified intention-to-treat** (mITT) population: all the patients included in the study whatever the inclusion/non-inclusion criteria are, who received at least one dose of XB2001 and with known vital status at 6 months. Patients will be analysed in their randomization arm. Patients will be analysed in their randomization arm.

Per protocole population (PP): the PP population is a subpopulation of the ITT population. It includes all patients without major protocol deviation (regarding inclusion and non-inclusion criteria as well as treatment received) with known vital status at 6 months and treated until progression (at least 80% of the cycles completed till progression, with the 2 chemotherapies, dose adaptation being allowed for trifluridine/tipiracil). Patients will be analysed in their randomization arm.

- **Safety population**: patients who received at least one dose of XB2001. Patients will be analyzed according to the treatment received irrespective of their randomization arm.

11.3 Statistical analysis of endpoints

A final statistical plan will be written before database is frozen. Quantitative data will be described using usual statistics: means, standard deviations, medians and ranges. These variables could also be categorized using clinical cut-offs, as reported in the literature. Qualitative variables will be described with percentages. The number of missing data will be reported. Comparison between arms will be performed in an exploratory manner.

- **Primary endpoint (6-month OS rate)** will be analyzed on the mITT population. The final analysis will be performed on the first 76 patients matching with the mITT definition. If the total number alive at 6 months is less than or equal to 48 in ethe experimental, the drug is rejected.

Overall and progression free survival will be estimated using the Kaplan-Meier method on the mITT population. Patients lost of follow up will be censored at the date of last follow up. Median times and rates will be presented at different time-points with their 95% confidence intervals. Median follow-up will be determined using the reverse Kaplan Meier method. In an exploratory manner, comparison between arms will be performed using the stratified log rank test. Power for these comparison wil be a posteriori determined. Conclusions would be drawing only in case of power higher than 80%.

- **Toxicities and DLTs** will be described by grade for each cycle and in total and categorized in body systems using MEDDRA coding. NCI-NCI-CTC version 5.0 will be used for grading.
- **Health-related quality of life** analysis will be conducted on the mITT population (all patients included whatever the inclusion/non-inclusion criteria, who received at least one dose of treatment) and with at least one health-related quality of life score available. Quality of life (QoL) scores will be described and compared at each clinical surveillance follow-up time: description using means, standard deviations, medians and ranges; comparison using Student T-test or Wilcoxon test. Impact of arm, time and interaction between arm and time will be determined using Generalized Linear Mixed Models. Analysis will be performed on complete data.

Analysis will be performed when the last subject included in the study will have at least 6 month of follow-up following last dose of treatment administration.

11.4 Managing the missing and non-valid data

For time-to-event endpoints patient not experiencing the event of interest and lost to follow-up before the last evaluation will be considered as censored at the time of last evaluation and taken into account in the statistical analysis.

Analysis of the other endpoints will be perfomed on complete data. No imputation of missing data are scheduled.

11.5 Interim analysis

11.5.1 Safety/Tolerance interim analysis

A safety analysis carried out by the Independent Data Monitoring Committe will be held within 1 month after 10 patients have been included in the experimental ARM and 10 patients in the control ARM. Enrollement will continue during analysis for safety. The primary endpoint for safety is the number of grade ≥ 4 drug-related adverse events. If 2 or more than 2 patients experienced grade≥4 adverse events (that are deemed probably related to study drug) then the trial will stop. This stopping rule uses a Fleming 1 step design with P0=0.05 (expected grade≥4 adverse events rate in the control arm), P1=0.25 (maximal grade≥4 adverse events rate in the experimental arm), unilateral alpha risk=0.10 and beta=0.25 (0.75 of power).

This interim analysis, as it doesn't concern the primary endpoint has no impact on the sample size.

11.5.2 Efficacy interim analysis

An interim analysis of the primary endpoint is planned when 26 patients included in each arm match with the mITT population definition. According to the Simon's design, if 15 or less patients are alive at 6 months in the experimental arm, the trial will end. This efficacy analysis has been taken into accound in the sample size calculation. The 6-month OS rate in the control arm will be used to ensure that the hypothesis used for the sample

size determination was appropriate. If not (more than 10 points of difference between observed and expected OS), boundaries and sample size could be adapted. Inclusion will not be stopped during this interim analysis.

11.6 Method of randomization

A randomization using minimization method will be used. Minimization variables will be the performance status (0 versus 1),the RAS mutational status, the MSS/MSI status and the addition of bevacizumab .

11.7 Conversion into Phase III trial

In case the trial is positive for the primary endpoint after the inclusion of 160 patients it can be converted into a phase III trial and additional patients would be included. The primary endpoint would be median OS. On the whole, 122 events are needed to have an 80% power to detect a hazard ratio of 0.60 for the experimental arm when the median OS in the control arm is 7.1 month, alpha risk =5% and the drop out rate 10%. With an accrual period of 24 months and a follow up of 6 months for each patient (obtained in the phase II study), 122 events will be reached with 190 patients.

12. OVERSIGHT COMMITTEES

12.1 Independant data monitoring committee

An inependant Data Monitoring Committee (IDMC), without direct involvment in the conduct of the study, will be set up specifically for the analysis of interim analysis results.

The IDMC will be composed of at least 3 expert members (2 clinicians and 1 statistician).

The IDMC will meet when 10 patients in the experimental arm and 10 patients in the control arm have been included in phase II to evaluated safety.

Depending on the recommendations of the IDMC following the first meeting, a second IDMC may meet after the inclusion of 26 patients in each arm.

The IDMC has a consultative role; it will inform the Sponsor and Steering Committee of their recommandations who will decided whether these should be implemented.

Data presented to the IDMC are strictly confidential.

12.2 Steering Committee

The steering executive committee (SC) will meet physically or by teleconference as required and in case of major protocol modification. The steering committe is composed by:

- The coordinating investigator
- The biostatistician
- The head of project of the sponsor
- Investigators
- Biologists
- Other participants if required

The role of the SC is to provide overall supervision of the study and ensure that the study is conducted in the rigorous standards set out in the Good Clinical practice. In particular, the SC should concentrate on progress of the trial, adherence to the protocol and patient safety. The SC will as well consider new information relevant to the study (e.g report from IDMC, results of other studies…) and should recommended appropriate action such as changes in the study protocol, additional patient information, stopping or extending the study.

The trial translational research program is under the supervision of SC which role is to consider hom the planned projects should be modified in their objectives or in their techniques or even if they are still relevant in regards to the current state of scientific knowledge. New proposed translationnal projects will also be evaluated by the SC for implementation.

The steering committee also determines to whom outcome results should be released prior to reporting of study results at the time specidfied in the protocol.

13. QUALITY ASSURANCE

13.1 Data collection

The study database will be hosted by the Unité de méthodologie, biostatistique et data management (UMBD) from the Centre georges-François Leclerc, 1 rue du Pr Marion, 21079 Dijon - France. Database management will be provided by an electronic Case Report Form (eCRF) developed using the Ennov Clinical® software (CLINSIGHT). The conditions for data transfer of all or part of the study database will be decided by the study sponsor in accordance with the written consent provided by each patient.

All data necessary to the research must be entered in a timely manner into the study eCRFs. CRFs will be completed by the investigator and any other designated members of their staff.

The following data will be collected:

- Demographic information.
- Disease and medical history.
- Clinical examination results, weight, height, vital signs, ECG.
- Blood and biological test results.
- Tumour evaluation results and copies of radiologic images for central review.
- Adverse events and serious adverse events occurring at each cycle.
- IP administration.

During the study, data clarification forms (DCFs) or queries may be sent for data consistency validation, by the UMBD. Corrections addressed by these queries should be made by persons authorized to complete the eCRF.

When using the eCRF, the traceability of access and changes made are traced by the software (audit trail).

13.2 Study monitoring

To ensure the authenticity and credibility of data in accordance with the “Décision portant sur les Bonnes Pratiques Cliniques, 24 November 2006”, the sponsor will establish a quality assurance system that will include:

- The management and the monitoring of the study according to the CGFL procedures;
- The quality control of data at the investigational centres by the monitor/clinical research associate (CRA) including:
  - ✓ verifying that the protocol, the current ICH-GCP guidelines, and the national regulatory requirements are adhered to,
  - ✓ verifying the informed consent and the eligibility of each patient participating in the study,
  - ✓ verifying that the CRF data is consistent and in agreement with the source documents,
  - ✓ verifying the notification of each SAE,
  - ✓ verifying the drug traceability (dispatching, storage, and accountability),
  - ✓ verifying that patients have or are not participating in another clinical study making them ineligible for this study.
- The auditing of participating investigational centres when deemed necessary;

The monitors/CRAs in charge of study monitoring will be mandated by the sponsor. Monitors/CRAs must have access to all patient data required for them to perform their duties/responsibilities in accordance with the national regulatory requirements. The monitors/CRAs are bound by professional secrecy as per national regulations. Written reports must be issued to ensure the traceability of monitoring visits.

In order to ensure the optimal research quality control, the investigator commits to provide the monitor/CRA direct access to all patient files.

13.3 Audits and Inspection

As part of its audit program, the sponsor, may audit some investigational centres. The centre and the investigator agree that audits can be performed by the Sponsor or any person duly authorized for a period during the study and for at least fifteen years after the end of the study (the last patient last visit).

A Competent Authority (CA) may also wish to conduct an inspection (during the study or after its completion). If a Competent Authority requests an inspection, the investigator must inform the sponsor immediately of this request. The investigator must provide the representatives of the Competent Authority direct access to source documents.

More generally, the investigator’s centre and the investigator undertake to devote the necessary time required for the audit/inspection procedures, control, and for provision of additional information requested by the sponsor or by a Competent Authority.

14. ETHICAL AND REGULATORY CONSIDERATIONS

14.1 General requirements

The study must be conducted in accordance with the French national regulatory requirements:

- Loi n°2012-300 du 5 mars 2012 (dite loi "Jardé") relative aux recherches impliquant la personne humaine
- Regulation (EU) 2016/679 on the protection of natural persons with regard to the processing of personal data and on the free movement of such data (General Data Protection Regulation),
- Loi Informatique et Libertés n° 78-17 du 6 janvier 1978 modifiée, relative à la protection des personnes physiques à l'égard des traitements de données à caractère personnel,
- Loi n° 2004-800 du 6 août 2004 modifiée, relative à la bioéthique,
- Décision du 24 novembre 2006 fixant les règles de bonnes pratiques cliniques pour les recherches biomédicales portant sur des médicaments à usage humain
- Bonnes Pratiques de Fabrication

14.2 Clinical Study Authorisation

Prior to the start of the study, the sponsor will submit the study protocol, patient information sheet(s), informed consent form(s), and other study documents as required by French national regulatory requirements, to the ANSM for authorisation and to the ethics committee (EC) for their written approval.

The sponsor will inform the ANSM and the EC, according to French national regulatory requirements, of any protocol amendments including any substantial modification requiring an ethical or regulatory reconsideration/re-evaluation of the study protocol.

Data recorded during this study are subject to computerised treatment by the Data Centre located in the Institut du Cancer de Montpellier, Montpellier, France in compliance with the “Loi Informatique et Libertés n° 78-17, 6 January 1978 modified” and “Délibération n° 2016-262 du 21 juillet 2016 portant modification de la méthodologie de référence pour les traitements de données personnelles opérés dans le cadre des recherches biomédicales (MR-001)”.

After the study, and in the case of storage, the storage of the collection of biological samples will be notified to the Minister of Research (and submitted to the EC to verify if any change in the purpose of the research has occurred).

14.3 Patient identification

All patients will receive a unique patient identification number when signing the informed consent form by the patient and before any study procedure is performed. This number will be used to identify the patient throughout the study and must be used on all study documents related to this patient. The patient identification number must remain constant throughout the study.

14.4 Patient information and consent

Prior to the participation of a patient in the study, the patient will be informed both verbally and in writing about the objectives of the study, its methods, anticipated benefits and potential risks, and the discomfort to which they may be exposed. All items must be explained by the investigator in a language and in terms that are easy to

understand by the patient. Patients will also be informed that their participation is voluntary and that they have the right to withdraw from the study at any time without giving the reason(s) and without any negative consequences for their subsequent care/treatment.

The patients must be given enough time to consider and decide whether to participate in the study.

Patients will confirm their consent in writing prior to starting the study and prior to any study-related procedures. The informed consent form must be personally dated and signed by the patient. An original will be filed in the Investigator Site File (ISF). Patients must be given with a copy of the patient information sheet and the signed informed consent form. An original could be provided to the patient if two originals are signed.

The patient is not obliged to give reason(s) for withdrawing from the study. However, the investigator should make a reasonable effort to ascertain the reason(s) while fully respecting the patient's rights. In case of any changes in the written patient information or informed consent form (ICF), the investigator will ensure that all patients, impacted by the changes that are still participating in the study receive the updated patient information in a timely manner and provide written consent for these changes by signing and dating the updated ICF.

In conformance with the data protection regulation, the patient may use their right to access to rectify or oppose the use of their personal data in the research. In these situations, the investigator shall inform the sponsor without delay in order to take the appropriate steps.

14.5 Changes to the study protocol

The study will be conducted in strict compliance with this protocol. Subsequent changes will be included in an amended version of the study protocol. The list and tracking of modifications and rationale will be provided in the amended version of the study protocol. Amended study protocols with substantial modifications will be submitted to the ANSM and to the EC by the sponsor, according to the French regulation. Amendments with substantial modifications to the protocol should be implemented only after approval by the ANSM and the CPP.

14.6 Sponsor responsabilities

The centre Georges-François Leclerc, the study sponsor who has initiated this study is accountable for the study management and for verifying that the financing schedule covers the anticipated expenses.

The sponsor's main responsibilities are:

- The writing of the protocol and subsequent amendments, as well as other study documents;
- The subscription of a civil-responsibility insurance;
- The obtaining of an European Drug Regulatory Authorities Clinical Trials (EudraCT) identification number;
- The request according to the French regulation the opinion of the EC, and authorisation from the ANSM for the initial protocol and subsequent amendments (if applicable);
- The notification of any suspected unexpected serious adverse reactions (SUSAR), according to the French regulation, to the ANSM, and the notification of this information to the EC and the physician-investigators of the study;
- Transfer of the information concerning the study according to local regulatory requirements to the following persons at the investigational centres: directors, pharmacists, and physician-investigators;
- Transfer of the information concerning any new event and the measures taken to the ANSM, as well as simultaneous notification of the EC, according to local requirements;
- The recording of the study in database research accessible to the public
- The recording of the study in the international database ClinicalTrials.gov and the INCa's "Register of French cancer clinical studies", before the enrolment of the first patient;
- The permanent assessment of the ongoing safety of the investigational drugs/therapy and the safety of patients;
- The transmission of the Development Safety Update Reports (DSUR) to the ANSM and EC according to French regulations;
- The notification of the beginning and the end of the study to the ANSM and EC, according to French regulations;

- The preparation of the final Clinical Study Report and its communication to the ANSM, according to French regulations;
- The transfer of study's results to the ANSM and EC, according to French regulation. The study results may also be communicated to the research participant, at their request, by the physician/investigator;
- The archiving of the study's essential documents for a minimal duration of 15 years after the end of the study.

14.7 Insurance compensation

The sponsor of the study certifies that it has taken out a civil liability insurance policy covering its civil liability for this clinical study. This insurance policy is in accordance with local laws and requirements. The insurance of the sponsor does not exempt the investigator and its team from maintaining their own liability insurance policy.

14.8 Investigator responsabilities

The principal investigator is responsible for the following:

- Providing the sponsor with their curriculum vitae (CV) and those of their collaborators, and provide evidence that the centre has the necessary resources to conduct the study. The CV must be recent (less than 12 months old), dated, and signed;
- Identify the members of their team who will participate in the study and to define each members responsibilities;
- Start recruiting patients only after approval by the sponsor;
- Be available for monitoring visits, audits, inspections, and investigator meetings (if applicable).

The principal investigator and all staff members participating in the study are responsible for the following:

- Ensuring the confidentiality of all data recorded during the study;
- Obtain the informed consent form dated and signed personally by each individual research participant before any study-specific selection procedure;
- Regularly complete the eCRF for each of the patients included in the study and allow the CRA mandated by the sponsor direct access to the source documents in order to validated the data collected in the eCRF;
- Declare to the sponsor as soon as being made aware of any serious adverse event occurring during the study according to guidelines provided in this protocol (see Section 8.2);
- Accept regular visits by the CRA(s), and possibly those of auditors as mandated by the sponsor or the inspectors of the regulatory authorities;

14.9 Human biological samples collections

Biological studies are necessary to improve the knowledge of diseases as they allow the development of new and more effective treatments. These studies use human biological samples (blood, tissue tumours) than can be collected from patients either while they receive medical care (examination, surgery) or specifically for the research purpose.

As for research aimed at studying the genetic characteristics of the patients, a consent form must be signed by each participating patient after he has been informed on the research undertaken, irrespective of the type of sample collected (already existing or specifically collected).

Additionally, it must be noted that the results of biological studies may be published under the condition that all patients' data are anonymised.

14.9.1 Strorage and use of disease assessment samples (blood, biopsy, tumour specimen)

During medical care or surgical interventions performed, biological samples of tissues and/or cells (blood, tumour tissue) have been collected for medical purposes. A part of these samples may be stored and used for scientific researches. For this study, tumour samples from a secondary or a primary site, as well as blood samples are required.

The patient will be informed via a patient information sheet and, in the absence of opposition of his part, biological samples for research will be prepared, stored, and used for this research.

The preparation, storage, and use of the biological samples will not modify the diagnosis, care, and treatment of the patient.

14.9.2 Collecting additional biological samples for research purpose

For this study, we will collect the following sample for the purpose of the ancillary studies:

- Tumour samples: biopsy from a secondary or a primary site will be obtained at baseline and after 8 weeks of treatment if patient consent to the ancillary study. The formalin-fixed and paraffin-embedded (FFPE) block should be sent or 15-20 white slides for inclusion and 10 slides for optional biopsy.
- Blood samples: samples collected in EDTA and heparin tubes at baseline, 12 days after treatment, 8 weeks, 4 months and at disease progression will be collected.

These additional biological samples will require written consent from the patient. A specific consent form, in addition to the consent form for participation in the clinical study, is revocable at any time. Similarly, at any time of the research, the patient has the opportunity to request the destruction of these samples.

15. DATA PROCESSING AND CONSERVATION OF DOCUMENTS AND DATA OF THE RESEARCH

15.1 Data processing

15.1.1 Under the responsability of the sponsor

The statistical data analysis will be transferred to the biostatistical unit of the UMBD under the responsibility of Dr Aurélie BERTAUT. All study data remain the property of CGFL, the research sponsor.

The software Clinsight® will be used for data entry, management and archiving of data. The statistical analysis will be performed using the SAS® 9.3 (Statistical Analysis System) software.

15.1.2 In the investigational site, when computerised medical record are used

If a computerised patient records are used in a participating centre to process or store data related to the study, the centre must:

- Verify and document that the computer system used to process the data is in conformity with the requirements in terms of data completeness, accuracy, and reliability with respect to the expected performances (quality validation);
- Define and follow the standardised procedures related to these systems;
- Ensure that these systems allow modifications of the collected data, that each modification is automatically authentified, and that the data cannot be removed (i.e. any change or modification of the data must be traceable);
- Set up and maintain a security control to prevent unauthorised access to the data;
- Establish and regularly update the list of persons authorised to have access and modify the data;
- Carry out appropriate backups of the data;
- Ensure confidentiality, whenever applicable (e.g. during data input);
- Ensure that the individual patient computerised data are processed in accordance with the “Loi Informatique et Libertés n° 78-17, 6 January 1978 modified”. If data are transformed while being processed, it should always be possible to compare them with the original observations/records. The computerised system used to identify the patients participating in the study must not be ambiguous and should allow the identification of all data collected for each patient while preserving their confidentiality in accordance with the “Loi Informatique et Libertés n° 78-17, 6 January 1978 modified”. The software Clinsight® will be used for data entry, management and archiving of data. The statistical analysis will be performed using the SAS® 9.3 (Statistical Analysis System) software.

15.2 Retention of documents by investigator sites

The investigator must maintain source documents for each study patient.

All information entered into the case report forms must be traceable and consistent with source documents, generally present in the patient's medical file. The source documents should contain all demographic and medical information, laboratory data, radiology, electrocardiograms, etc.…, including signed informed consent form.

The investigator must retain essential documents as listed below. The investigator agrees to adhere to the document retention procedures by signing the protocol. Essential documents include:

- Approvals from the CPP for the study protocol and all relevant amendments;
- Authorisations from the ANSM for the study protocol and all relevant amendments;
- All source documents and laboratory records;
- CRF copies;
- Patients' informed consent forms;
- Investigator master file (IMF) and Investigator master file-pharmacy (IMF-P);
- Any other pertinent study document.

All study documents must be kept in a locked and secured place and are considered as confidential.

Data will be archived under the responsibility of the principal investigator of each participating centre according to the "Décision portant sur les Bonnes Pratiques Cliniques, 24 November 2006". The archives will be kept as well as a list of patient identifications for a minimum period of 15 years after the end of the trial. CGFL will inform the investigational centres when the trial-related records are no longer required.

The investigational centre may destroy the data only after the sponsor's written authorisation.

16. DATA OWNERSHIP AND CONFIDENTIALITY

By signing the protocol, the investigator agrees to keep all information provided by the CGFL in strict confidence and to request similar confidentiality from his/her staff. This obligation does not include information provided to the patients or information already publically available.

Study documents provided by the CGFL (protocols, investigators brochures, CRFs and other material) will be stored appropriately to ensure their confidentiality. The information provided by the CGFL to the physician-investigator may not be disclosed to others without direct written authorisation from the CGFL.

The physician-investigator agrees to not publish, divulge or use in any manner, directly or indirectly, the scientific and technical information, and results related to the study.

17. PUBLICATION RULES

All information resulting from this study is considered as confidential, at least until appropriate analysis and checking has been completed by the sponsor, the principal investigator, and the statistician of the study.

Any publication, abstract, or oral presentations including results of the study must be submitted to the sponsor (CGFL) for approval.

Additionally, all communications, manuscripts, or oral presentations must include a section mentioning the CGFL as well as any institution, physician-investigator, cooperating or collaborative research group, scientific society that has contributed to the study, including organisations that have provided financial support.

The first author and writer of the main publication will be the principal investigator. She/he may however designate another person to (co-) write the publication.

As for the main publication authors are quoted in the following order:

- the study coordinator (first or last author);
- the other investigators will appear in the list of co-authors in decreasing order, according to the number of recruited patients regardless of the importance of the cooperating group they belong to;

- a person representing each cooperating group among the investigational centres not quoted among the sites that have the highest rates of recruitment;
- the statistician (The statistician's position is among the first three authors, the last or the penultimate author of the publication);
- a sponsor's representative.

Similarly, publication of the sub-studies (e.g. biological/ancillary studies) will include persons who have carried out the sub-studies as well as the names of all individuals who have contributed to these sub-studies and a sponsor representative.

It is desirable to include the contributors from weakly recruiting centres and who have not been mentioned in the first article in the later publications.

Any conflict regarding publication authorship will initially be submitted to the study IDMC and then to the CSR (Comité Stratégique Recherche [Strategic Research Committee]) for resolution in case of major disagreement.

The CGFL will arbitrate and rule any dispute that may arise.

18. REFERENCES

1. Ferlay J, et al. Cancer incidence and mortality worldwide: Sources, methods and major patterns in GLOBOCAN 2012: Globocan 2012. Int J Cancer. 2015 Mar 1;136(5):E359–86.

2. Van Cutsem E, et al. ESMO consensus guidelines for the management of patients with metastatic colorectal cancer. Ann Oncol Off J Eur Soc Med Oncol. 2016;27(8):1386–422.

3. Grothey A, et al. Regorafenib monotherapy for previously treated metastatic colorectal cancer (CORRECT): an international, multicentre, randomised, placebo-controlled, phase 3 trial. Lancet. 2013 Jan;381(9863):303–12.

4. Lenz H-J, Stintzing S, Loupakis F. TAS-102, a novel antitumor agent: A review of the mechanism of action. Cancer Treat Rev. 2015 Nov;41(9):777–83.

5. Mayer RJ, et al. Randomized Trial of TAS-102 for Refractory Metastatic Colorectal Cancer. N Engl J Med. 2015 May 14;372(20):1909–19.

6. Overman MJ, et al. Durable Clinical Benefit With Nivolumab Plus Ipilimumab in DNA Mismatch Repair–Deficient/Microsatellite Instability–High Metastatic Colorectal Cancer. J Clin Oncol. 2018 Mar 10;36(8):773–9.

7. Le DT, et al. PD-1 Blockade in Tumors with Mismatch-Repair Deficiency. N Engl J Med. 2015 Jun 25;372(26):2509–20.

8. Gutting T, Burgermeister E, Härtel N, Ebert MP. Checkpoints and beyond – Immunotherapy in colorectal cancer. Semin Cancer Biol. 2019 Apr;55:78–89.

9. Tauriello DVF, et al. TGFβ drives immune evasion in genetically reconstituted colon cancer metastasis. Nature. 2018 22;554(7693):538–43.

10. Van den Eynde M, et al. The Link between the Multiverse of Immune Microenvironments in Metastases and the Survival of Colorectal Cancer Patients. Cancer Cell. 2018 Dec;34(6):1012-1026.e3.

11. Pagès F, et al. International validation of the consensus Immunoscore for the classification of colon cancer: a prognostic and accuracy study. The Lancet. 2018 May;391(10135):2128–39.

12. Grivennikov SI, Greten FR, Karin M. Immunity, Inflammation, and Cancer. Cell. 2010 Mar;140(6):883–99.

13. Lurje G, et al. Polymorphisms in interleukin 1 beta and interleukin 1 receptor antagonist associated with tumor recurrence in stage II colon cancer: Pharmacogenet Genomics. 2009 Feb;19(2):95–102.

14. Hagemann T, Balkwill F, Lawrence T. Inflammation and Cancer: A Double-Edged Sword. Cancer Cell. 2007 Oct;12(4):300–1.

15. Apte RN, et al. The involvement of IL-1 in tumorigenesis, tumor invasiveness, metastasis and tumor-host interactions. Cancer Metastasis Rev. 2006 Dec 6;25(3):387–408.

16. Voronov E, et al. IL-1 is required for tumor invasiveness and angiogenesis. Proc Natl Acad Sci. 2003 Mar 4;100(5):2645–50.

17. Chalmin F, et al. Stat3 and Gfi-1 Transcription Factors Control Th17 Cell Immunosuppressive Activity via the Regulation of Ectonucleotidase Expression. Immunity. 2012 Mar;36(3):362–73.

18. Hurtado CG, Wan F, Housseau F, Sears CL. Roles for Interleukin 17 and Adaptive Immunity in Pathogenesis of Colorectal Cancer. Gastroenterology. 2018 Dec;155(6):1706–15.

19. Dmitrieva-Posocco O, et al. Cell-Type-Specific Responses to Interleukin-1 Control Microbial Invasion and Tumor-Elicited Inflammation in Colorectal Cancer. Immunity. 2019 Jan;50(1):166-180.e7.

20. Kim B, Lee Y, Kim E, Kwak A, Ryoo S, Bae SH, Azam T, Kim S, Dinarello CA. The interleukin-1alpha precursor is biologically active and is likely a key alarmin in the IL-1 family of cytokines. Front Immunol. 2013;4:391. doi:10.3389/fimmu.2013.00391

21. Ricote M, García-Tuñón I, Bethencourt FR, Fraile B, Paniagua R, Royuela M. Interleukin-1 (IL-1alpha and IL-1beta) and its receptors (IL-1RI, IL-1RII, and IL-1Ra) in prostate carcinoma. Cancer.2004;100:1388–1396. doi:10.1002/cncr.20142.

22. Singer CF, Kronsteiner N, Hudelist G, Marton E, Walter I, Kubista M, Czerwenka K, Schreiber M, Seifert M, Kubista E. Interleukin 1 system and sex steroid receptor expression in human breast cancer: interleukin 1alpha protein secretion is correlated with malignant phenotype. Clin Cancer Res. 2003;15 (9):4877–4883.

23. Hickish T, et al. MABp1 as a novel antibody treatment for advanced colorectal cancer: a randomised, double-blind, placebo-controlled, phase 3 study. Lancet Oncol. 2017 Feb;18(2):192–201.

24. Kurzrock R, et al. Interleukin-1 receptor antagonist levels predict favorable outcome after bermekimab, a first-in-class true human interleukin-1α antibody, in a phase III randomized study of advanced colorectal cancer. OncoImmunology. 2019 Mar 4;8(3):1551651.

25. Bruchard M, et al. Chemotherapy-triggered cathepsin B release in myeloid-derived suppressor cells activates the Nlrp3 inflammasome and promotes tumor growth. Nat Med. 2013 Jan;19(1):57–64.

26 Isambert N, et al. Fluorouracil and bevacizumab plus anakinra for patients with metastatic colorectal cancer refractory to standard therapies (IRAFU): a single-arm phase 2 study. OncoImmunology. 2018 Sep 2;7(9):e1474319.

27. Coleman KM, Gudjonsson JE, Stecher M. Open-Label Trial of MABp1, a True Human Monoclonal Antibody Targeting Interleukin 1α, for the Treatment of Psoriasis. JAMA Dermatol. 2015 May;151(5):555-6. doi: 10.1001/jamadermatol.2014.5391.

28. Carrasco D, Stecher M, Lefebvre GC, Logan AC, Moy R. An Open Label, Phase 2 Study of MABp1 Monotherapy for the Treatment of Acne Vulgaris and Psychiatric Comorbidity. J Drugs Dermatol. 2015 Jun;14(6):560-4.

29. Kanni T et al. MABp1 Targeting Interleukin-1Alpha for Moderate to Severe Hidradenitis Suppurativa not Eligible
for Adalimumab: A Randomized Study. J Invest Dermatol. 2017 Nov 9.

30. Gottlieb A. et al. A Phase II Open-Label Study of Bermekimab in Patients with Hidradenitis Suppurativa Shows Resolution of Inflammatory Lesions and Pain. J Invest Dermatol. 2020 Aug;140(8):1538-1545.e2. doi: 10.1016/j.jid.2019.10.024. Epub 2020 Jan 29.

31. Hong DS. et al. MABp1, a first-in-class true human antibody targeting interleukin-1α in refractory cancers: an open-label, phase 1 dose-escalation and expansion study. Lancet Oncol. 2014 May;15(6):656-66. doi: 10.1016/S1470-2045(14)70155-X. Epub 2014 Apr 17.

32. Hickish T. et al. MABp1 as a novel antibody treatment for advanced colorectal cancer: a randomised, double-blind,
placebo-controlled, phase 3 study. Lancet Oncol. 2017 Feb;18(2):192-201. doi: 10.1016/S1470-2045(17)30006-2. Epub 2017 Jan 14.

33. Hong DS. et al. Xilonix, a novel true human antibody targeting the inflammatory cytokine interleukin-1 alpha, in non-small cell lung cancer. Invest New Drugs. 2015 Jun;33(3):621-31. doi: 10.1007/s10637-015-0226-6. Epub 2015 Mar 31.

34. El Sayed H. et al. A randomized Phase II study of Xilonix, a targeted therapy against interleukin 1α, for the prevention of superficial femoral artery restenosis after percutaneous revascularization. J Vasc Surg. 2016

Jan;63(1):133-41.e1. doi: 10.1016/j.jvs.2015.08.069. Epub 2015 Oct 1.

35. Timper K. et al. Safety, pharmacokinetics, and preliminary efficacy of a specific anti-IL-1alpha therapeutic antibody (MABp1) in patients with type 2 diabetes mellitus. J Diabetes Complications. Sep-Oct 2015;29(7):955-60.doi: 10.1016/j.jdiacomp.2015.05.019. Epub 2015 Jun 3.

36 Carrato, A., Falcone, A., Ducreux, M. et al. A Systematic Review of the Burden of Pancreatic Cancer in Europe: Real-World Impact on Survival, Quality of Life and Costs. J Gastrointest Canc 46, 201–211 (2015). https://doi.org/10.1007/s12029-015-9724-1.

37. Jang et al. Mass spectrometric quantification of neutral and sialylated N-glycans from a recombinant therapeutic
glycoprotein produced in Chinese hamster ovary cell lines. Anal. Biochem. 386 (2009) 228-236.

38. Miossec. Anti-interleukin 1α autoantibodies. Ann Rheum Dis 2002; 61: 577-579.

39. Gallay et al. Characterization and detection of naturally occurring antibodies against IL-1α and IL-1β in normal human plasma. Eur Cytokine Network 1991; 2: 329-338.

40. Hong D. et al. MABp1, a first-in-class true human antibody targeting interleukin-1α in refractory cancers: an open-label, phase 1 dose-escalation and expansion study. Lancet Oncol. 2014 May;15(6):656-66. doi: 10.1016/S1470-2045(14)70155-X. Epub 2014 Apr 17.

41. Hickish T. et al. MABp1 as a novel antibody treatment for advanced colorectal cancer: a randomised, double-blind, placebo-controlled, phase 3 study. Lancet Oncol. 2017 Feb;18(2):192-201. doi: 10.1016/S1470-2045(17)30006-2. Epub 2017 Jan 14.

42. Hendifar, A. E., Kim, S., Tighiouart, M., Hautamaki, E., Kim, H., Ng, C., . . . Gong, J. (2020). A phase I study of nanoliposomal irinotecan and 5-fluorouracil/folinic acid in combination with interleukin-1-alpha antagonist for advanced pancreatic cancer patients with cachexia (OnFX). Journal of Clinical Oncology, 38(15_suppl), 4634-4634. doi:10.1200/jco.2020.38.15_suppl.4634.

43 . Laheurte C, et al. Immunoprevalence and magnitude of HLA-DP4 versus HLA-DR-restricted spontaneous CD4 + Th1 responses against telomerase in cancer patients. OncoImmunology. 2016 May 3;5(5):e1137416.

44 Pécuchet N, et al. Analysis of Base-Position Error Rate of Next-Generation Sequencing to Detect Tumor Mutations in Circulating DNA. Clin Chem. 2016 Nov;62(11):1492–503.

46. Hendifar AE, et al. A phase I study of nanoliposomal irinotecan and 5-fluorouracil/folinic acid in combination with interleukin-1-alpha antagonist for advanced pancreatic cancer patients with cachexia (OnFX). JCO. 2020 38:15_suppl, 4634-4634

47. Josep Tabernero, Julien Taieb, Gerald W Prager, Fortunato Ciardiello, Marwan Fakih, Catherine Leger, Ronan Fougeray, Nadia Amellal , Eric van Cutsem .Trifluridine/tipiracil plus bevacizumab for third-line management of metastatic colorectal cancer: SUNLIGHT study design.

48. Pfeiffer, Mette Yilmaz, Sören Möller, Daniela Zitnjak, Merete Krogh, Lone Nørgård Petersen, Laurids Østergaard Poulsen, Stine Braendegaard Winther, Karina Gravgaard Thomsen, Camilla Qvortrup.TAS-102 with or without bevacizumab in patients with chemorefractory metastatic colorectal cancer: an investigator-initiated, open-label, randomised, phase 2 trial. The Lancet Oncology Volume 21, Issue 3, March 2020, Pages 412-420

49. Thomas Walter , Astrid Lievre, Romain Coriat, David Malka, Farid Elhajbi, Fréderic Di Fiore , Olivia Hentic, Denis Smith, Vincent Hautefeuille, Guillaume Roquin, Marine Perrier, Laetitia Dahan, Victoire Granger, Iradj Sobhani, Laurent Mineur, Patricia Niccoli, Eric Assenat, Jean-Yves Scoazec, Karine Le Malicot, Côme Lepage, Catherine Lombard-Bohas. Bevacizumab plus FOLFIRI after failure of platinum-etoposide first-line chemotherapy in patients with advanced neuroendocrine carcinoma (PRODIGE 41-BEVANEC): a randomised, multicentre, non-comparative, open-label, phase 2 trial. Lancet Oncol 2023 Mar;24(3):297-306
